# Using Mitochondrial and Nuclear Sequence Data for Disentangling Population Structure in Complex Pest Species: A Case Study with *Dermanyssus gallinae*

**DOI:** 10.1371/journal.pone.0022305

**Published:** 2011-07-25

**Authors:** Lise Roy, Thierry Buronfosse

**Affiliations:** 1 Anses Lyon, Unité Résistance aux produits phytosanitaires, Lyon, France; 2 VetAgro Sup, Campus Vétérinaire de Lyon, Marcy-l'Etoile, France; Martin-Luther-Universität Halle, Germany

## Abstract

Among global changes induced by human activities, association of breakdown of geographical barriers and impoverishered biodiversity of agroecosystems may have a strong evolutionary impact on pest species. As a consequence of trade networks' expansion, secondary contacts between incipient species, if hybrid incompatibility is not yet reached, may result in hybrid swarms, even more when empty niches are available as usual in crop fields and farms. By providing important sources of genetic novelty for organisms to adapt in changing environments, hybridization may be strongly involved in the emergence of invasive populations.

Because national and international trade networks offered multiple hybridization opportunities during the previous and current centuries, population structure of many pest species is expected to be the most intricate and its inference often blurred when using fast-evolving markers. Here we show that mito-nuclear sequence datasets may be the most helpful in disentangling successive layers of admixture in the composition of pest populations. As a model we used *D. gallinae* s. l., a mesostigmatid mite complex of two species primarily parasitizing birds, namely *D. gallinae* L1 and *D. gallinae* s. str. The latter is a pest species, considered invading layer farms in Brazil. The structure of the pest as represented by isolates from both wild and domestic birds, from European (with a focus on France), Australian and Brazilian farms, revealed past hybridization events and very recent contact between deeply divergent lineages. The role of wild birds in the dissemination of mites appears to be null in European and Australian farms, but not in Brazilian ones. In French farms, some recent secondary contact is obviously consecutive to trade flows. Scenarios of populations' history were established, showing five different combinations of more or less dramatic bottlenecks and founder events, nearly interspecific hybridizations and recent population mixing within *D. gallinae* s. str.

## Introduction

Some global changes induced by human activities impact the evolution of pest species and may have strong incidence on their population genetic structure. Not only human transport and commerce keep introducing unprecedented alterations in the distribution of the earth's biota, but also cultivation and husbandry apparently facilitate many biotic invasions [1]. Multiple breakdowns of geographical barriers consecutive to expanding export/import markets and exponentially increasing air/truck/train travels make new migrations for pest populations possible [2]. Once entered in a new area, fundamentally impoverished native species richness of agroecosystems such as crop fields and farms are very likely to facilitate the establishment of non-native species or populations [3]. As a consequence, decreasing diversification in production systems and extension of crop and animal trade networks provide growing numbers of opportunities for long separated pest populations to get in contact within anthropized areas and is susceptible to suddenly and repeatedly generate introgressive hybridization. This may involve deeply diverging lineages if intrinsic zygotic incompatibility was not yet reached. The overlap between pest species and invasive species is likely to be even more important because hybridization provides important sources of genetic novelty for organisms to adapt in changing environments [4], [5].

Such population mixing, followed in some cases by nearly interspecific hybridizations (see below lexical note) makes population structure within a given pest species very difficult to be disentangled. Many studies explore the genetic structure of hybrids in the framework of conservative biology [6], [7], as in some case hybridization may finally result in the decline of one or both of the hybridized entities, either due to sterile or partially sterile hybrids (no introgression), or due to hybrid swarms leading to genomic extinction (introgression present) [8]. Such studies are only possible if the different entities involved in hybridization are still available as such. In contrast, when introgressive hybridization has occurred since a time long enough to reach hybrid swarm and/or new speciation events, the resulting tight reticulation between deeply divergent lineages is likely to blur the analysis. This is especially expected in invasive species, of which many have been shown to result from multiple introduction events and hybridization [4]. Moreover, because of the recurrence of human-induced breakdown of geographical barriers in anthropized areas, some pest isolates are likely to contain not only hybrid individuals, but also, in case of very recent secondary contact or of true cryptic species, unadmixed individuals from every previously separated population (motley populations).

At least in parasites, whereas cytoplasmic data are DNA sequences in most population genetics studies, the most commonly used nuclear markers are fast-evolving ones such as microsatellites (see [9] for review). However, because of their slower evolution rate, nuclear sequence data may still bring useful information to the study of such life histories. For instance, in Hoban et al. [6], the use of microsatellite loci for studying hybridized populations generated only rough estimates. In contrast, heterozygous indels have proven to be reasonably informative in some hybridized fish species [10]. Despite the potential of nuclear sequences, population geneticists remain hesitant in applying them for many reasons. These include sequencing costs and related problems with linkage disequilibrium (LD) between pairs of sites in a given DNA fragment [11], and heterozygosity (e.g. Brisson et al. [12]).

In this work, we argue that exploration through Bayesian hierarchical clustering and F-statistics on mitochondrial and nuclear sequence datasets can help disentangle successive layers of admixture in the composition of pest populations, provided that the effects of LD have been checked. Successive secondary contact events following long isolations is expected to generate a tangle of immigration histories, implying both motley populations mainly composed of unadmixed individuals of divergent populations and hybrid swarms that is populations containing nothing other than hybrid individuals, not to mention intermediate states. A number of population genetic features are expected when analyzing sexual species which have undergone such histories: (a) In the case where hybridization between incipient species (see lexical note below) occur, followed by speciation reversal as described by Seehausen et al. [5], several levels of population structure should be detected (hybridization between species, then differentiation of new populations…) by the mean of hierarchical clustering analyses. (b) A given gene might reveal isolation of any sibling species at the same level as of more than one lineage representing the targeted species. (c) Nuclear and mitochondrial sequence data should be able to report different steps of the admixture. Indeed, due to theoretically smaller effective populations size of mitochondrial vs nuclear DNA (a quarter in diplodiploid organisms [13]; a third in present haplodiploid organism [14], [15]), one might expect obtaining an older view of population structuration in nuclear analyses than in mitochondrial analyses, i.e. some monophylies may be reached in the last ones, not yet in the first ones. All the more, heterozygozity involving deeply divergent nuclear lineages is expected to be recurrent. (d) The ratio of the nucleotidic mitochondrial diversity π compared to the nuclear π should be significantly increased in some isolates, due to recent – if not just contemporary – contact between newly isolated populations.

*Dermanyssus gallinae* (De Geer, 1778) s. str. is an important pest in layer industries [16] and can also be found *in natura* and as such represents an adequate model for such analyses. This system provides highly variable mitochondrial and nuclear DNA sequences as compared with closely related species within the genus *Dermanyssus* [17], [18]. It is a micropredatory mite species and, like bedbugs, it is obligatorily hematophagous, but has rapid blood meal and does not stay on host [19]. Most of time is spent off the host, egg laying and exuviating in or around the nest's walls. *D. gallinae* s. l. is a complex of species, as it contains at least two cryptic species, *D. gallinae* s. str. and *D. gallinae* L1 [17], [20]. *D. gallinae* s. str. (all but L1 populations) is the only generalist species at least among the six *Dermanyssus* species commonly found in France, and is the only *Dermanyssus* species of economic importance in Europe [20], [21]. It is assumed to be an invasive organism in Brazilian layer farms, where it entered around the middle of 20^th^ century and had superseded autochthonous *Ornithonyssus bursa* (Berlese, 1888) ca. 50 years later [22].

Symmetrical mito-nuclear paraphyly within genus *Dermanyssus* and the conspicuously shorter length of the branch basal to the *D. gallinae* complex as compared to sibling species *D. apodis* Roy, Dowling, Chauve and Buronfosse, 2009 were interpreted as possible clues to some hybridizations by Roy et al. [17].

In an attempt to establish variable mitochondrial and nuclear sequence data as useful markers to study intricate histories of populations, including hybridization between long isolated lineages, we took advantage of established ecological (wide host spectrum and marked preference for domestic birds in *D. gallinae* s. str. unlike *D. gallinae* L1 [20], [21]) and genetic (high DNA variability in cytochrome oxidase I and in Tropomyosin intron n in *D. gallinae* s. str. unlike *D. gallinae* L1 [17]) characteristics of this species complex to investigate several layers of its history. Especially, we aimed at establishing:

detectable imprint left by probable hybridization(s) through such a human-influenced history
demographic scenarios which are distinguishable using mito-nuclear sequence data within *D. gallinae* complex
in a more specific point of view, the most probable causes for *D. gallinae* s. str. to have turned out to be an invasive species in Brazilian farms (is it a lineage that resulted from hybridization events in Brazilian farms or that was just transported to South America recently due to European settling events ?).

In order to check whether results were not biased by within fragment linkage disequilibrium (LD) between pairs of sites, sequence data were split into two groups, whole datasets and datasets excluding pairs of site with a r_LD_>0.5, and both batches of results compared, as recommended for Bayesian assignment and clustering [11]. Regarding a rather ancient time scale, the inclusion of L1 in hierarchical clustering analyses is expected to serve as a level reference in order to identify nearly interspecific hybridization events (see lexical note below). At a much more recent time scale, possible recent contact between isolated populations due to current expansion of commercial networks will be searched for by assuming two main possible doors for pest dissemination: wild fauna and trade flow. The expected relative difference in coalescence time between mitochondrial and nuclear sequences will help discriminating between both time scales.

## Materials and Methods

### Lexical note

Because hybridization implies new genetic admixture between more or less long separated populations and because speciation is a gradual process, such events by definition lie at the very boundary between species and intraspecific entities. As a result, any study aiming at investigating them raises the problem of the definition of species. Therefore, we need to beforehand set some lexical definitions.

#### Species

We consider here species as defined following the internodal concept consisting of a fragment of the Global Genealogical Network (GGN) branch segment between two nodes of the tree, with each node being either a branching point (speciation) or a dead-end (extinction) [23]. However, it is important to clearly distinguish between the concept itself and its practical implications in species delimitation, keeping in mind that available species delineations are necessarily fallible hypotheses, (1) because drawn from dramatically incomplete sets of data, (2) because a species is a historical entity [23]. Concerning the latter, since extant species at a given time are most likely to represent only distal tips of the GGN (leaves of the outlined tree), the required absolute irreversibility of reproductive isolation is not allowed to be confirmed in studies dealing with contemporaneous generations only (not yet node or dead-end distally), as most if not all studies on pest species. As a result, species delineations remain more of an anticipation of the future history of life or of a bet laid on it, although they can be strongly supported by the congruence of several lines of evidence.

#### Incipient species

Because speciation is a gradual process and because human-induced breakdown of geographical barriers allows naturally improbable admixture, notably at a latest time within the critical window in speciation during which intrinsic postzygotic isolation is absent or incomplete, we decided to take into account some isolation even through available data show it does have subsequently been broken down. If genetic divergence from lineage split to secondary contact is revealed considerable and comparable to divergence with a sibling species and in case of evidence for hybridization between so deeply diverging entities, the term incipient species will designate family lineage partitions [23] from the first generations following the lineage split to the last generations just before interbreeding.

#### Speciation

Since fertile hybridization between two alleged species is by definition genuine evidence for them belonging to the same species [23], [24], the derived word speciation should be accepted only in instances where no interbreeding has ever occurred. However, for the same reasons as above, we decided that strictly avoiding the use of speciation in case of secondary geographical contact followed by introgressive hybridization would render discussion very uneasy and propose to use the word speciation for designating isolation at a time comparable to isolation of a close currently alleged species. We consequently adopt the phrase “reversal of speciation” as used by Seehausen et al. [5] for designating emergence of hybrid swarm in such contexts.

#### Nearly interspecific hybridization

For the same reasons, because the internodal species definition implies that so-called “interspecific hybridization” is nonsensical, we propose to name “nearly interspecific hybridization” the result of admixture between lineages almost as differentiated from each other as from a sibling species.

#### Lineage

A lineage designates the descendants of a common ancestor, which form a fragment of the GGN, not necessarily a whole segment, but are, at some point, reproductively isolated from other organisms. “Lineage” is used instead of “species” in cases where the probability that reproductive isolation is irreversible has never been questioned or is not sufficiently supported. In some instances, it corresponds to family lineages *sensu* Samadi and Barberousse [24].

#### Entity

The word “entity” is used as a generic term for “lineage” and “species”.

#### Population and isolate

A population is a group of mite individuals belonging to a single species and occupying an individual nest or a group of nests in a bird colony (wild avifauna) or a single building (farms), or, in some case, a single point within a building. An isolate is a random sample of individuals which are representative of a given population.

### Collection and DNA extraction

The location, host species, mite species, accession numbers associated with mite individuals under test are listed in Table S1. Mite isolates have been sampled from wild bird nests or from farms as described in [21]. Nests were analyzed using De Lillo's method, which involves immersion of the nest followed by filtering, except that no sodium hypochlorite was added to the water solution to wash the stack of sieves and that the sieves had a somewhat different mesh width (top to bottom: 2,500 µm, 1,400 µm, 180 µm, 100 µm).

From each population, 11–24 individuals have been separately sequenced (isolates^+^). In some cases, a reduced number of individuals have been added (isolates^−^; some L1 isolates, 2 isolates from farm CON, see Table S1 and Figure S1).

### Reference *D. gallinae* s. str. isolates

Among *D. gallinae s. str.* isolates under test, three are considered as references. Two wild isolates IL and ROL are considered as references for the “natural” condition. These two isolates are expected to provide the natural distribution of haplotype and nucleotidic diversity in *D. gallinae* s. str. Note that only two wild isolates^+^ have been sequenced because *D. gallinae*, despite its wide host range, is rather sparsely encountered in the wild avifauna. However, three additional isolates^−^ had been involved in the study of Roy et al. [17], labelled JBO, Woodp, LB18, which provided Tpm sequences closest to the present wild isolates'. The two present wild isolates^+^ are labelled IL and ROL and have respectively been sampled in nests of Common Starling (in the Netherlands) and in nests of European Roller (in Southern France). The three previously analyzed isolates^−^ JBO, Woodp and LB18 had been respectively sampled in Great Tit (in Southern France), Great Spotted Woodpecker (in Central Eastern France) and Common House Martin (in the Center of France). The strain SK, which has been kindly provided by O. Kilpinen and has been reared at lab since 1997, is considered here as a reference strain from the following points of view, thanks to its known history: smallest approximate size of founder population at the entrance into laboratory (several dozens of individuals maximum), around 52 (28.2–112.7) generations between 1997 and sampling in 2010, i.e. much reduced if compared to wild avifauna and farms, because mites were fed, on average, every 3 (2–4) weeks (O. Kilpinen, pers. comm.).

### Molecular markers

DNA was extracted from single adult females and sequenced following [21], [25].

A 543 bp fragment of the cytochrome oxidase I (COI) coding gene and a 663–698 bp fragment of the Tropomyosin (Tpm) gene including a large intron, flanked by small exon parts at both ends have been PCR amplified and sequenced following [17]. As in this study, when the diploid fragment of Tpm was heterozygous, it almost systematically included heterozygous indels at one or more of the series previously identified [17], two additional internal sequencing per individual using two different primers designed on one of the indel region (the one containing the indel fragment, the other without it) allowed successful sequencing of both alleles separately. As a result, obtained Tpm sequences are phased on the site point of view in present study (not on the gametic point of view). This allowed testing individual Tpm genotypes using F-statistics.

### Scenarios to be tested

Mitochondrial (“Lmt-”) and nuclear lineages (“Ln-“), as well as some precise Tpm (“Tro-“) and COI (“Co-”) haplotypes will be referred to in present paper following the nomenclature established by [17].

To investigate the congruence of obtained data, we have explored the most probable causes for revealed patterns by testing different scenarios for nearly interspecific hybridization, gene flows and demographic history. For such a purpose, we have confronted results obtained for different groups of isolates to each another in populations genetics analyses, in relation with available information. Detailed information on the hypotheses under test and used methods for analyses can be found in Methods S1.

## Results

To test scenarios for nearly interspecific hybridization, gene flows and demographic history, we genotyped 469 adult females at the two fragments of COI and Tpm described in [17]. Ninety sites of 543 were polymorphic in COI (number of haplotypes h = 40, haplotype diversity Hd = 0.894). One hundred and nine of 729 aligned sites (663–698 per individual) in Tpm were polymorphic with gaps unconsidered (h = 32, Hd = 0.752) and 185 with gaps as the fifth state (h = 39, Hd = 0.754). Additionally, we genotyped 16 adult females from a North-Western French farm (farm BAU) at the COI fragment to complement haplotype distribution in the North-Western France. All of these individuals provided a single haplotype (Co_1), which was often encountered in French farms. Accession numbers of obtained sequences are provided in Table S1 and form two alignments available as Popsets online, representing Tpm and COI whole datasets.

Most F_IS_ per isolate gave non significant values, suggesting that Tpm alleles are in Hardy-Weinberg equilibrium. This is at the exception of BREa and BREb, which show some HWD (positive F_IS_ values, with P>0.05). As a result, we did not consider Tpm alleles to be independent for testing the significance of population differentiation, and rather used the genotype as the randomization unit instead of the allele in permutation tests of F_ST_ estimates.

**Figure 1:**
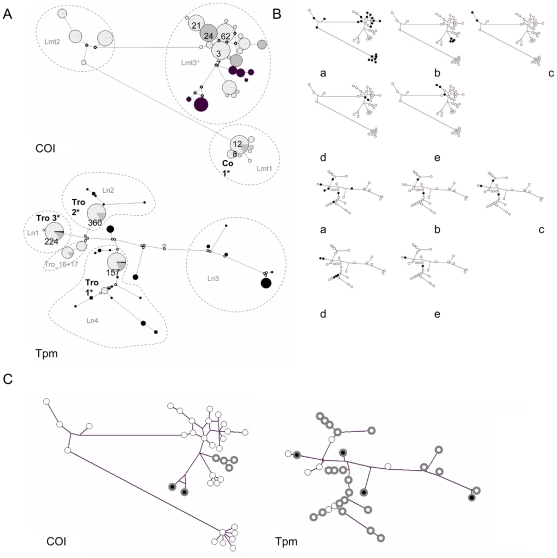
Haplotype MJN networks with post-processing parsimony analysis. A. Bird type information (black areas, wild birds; grey areas, chicken; squared areas, layer). *Top*, COI. *Bottom*, Tpm exon n, intron n, exon n+1. The size of the circles is proportional to the haplotype frequency, except that an upper size limit has been set to 20 for COI topologie and 40 for Tpm topology (keep in mind that Tpm gene is diploid). Numbers inside or close to circles appear in cases where the limit is exceeded and represent the observed number of occurrence. The length of links between haplotypes is proportional to the number of mutated positions. The median vectors that represent hypothetical intermediates or unsampled haplotypes are shown in small open circles. The most important haplotype (most frequent) are labelled (label followed by *) following the designation used in [17]. In COI (*top*), dotted lines delineate lineages Lmt1, Lmt2 and Lmt3 as defined in [17]. Lmt3^+^ represents lineage Lmt3 plus basal haplotypes not included in any lineages in [17]. In Tpm (*bottom*), dotted lines delineate lineages Ln1, Ln2, Ln3, Ln4 and the clade Tro_16+Tro_17 as defined in [17]. B. Geographical origin of farm individuals in which haplotypes have been isolated (a, France, b, Denmark, c, Poland, d, Brazil, e, Australia). *Top*, COI. *Bottom*, Tpm. Haplotypes found in each country are represented by solid circles, whereas open circles represent haplotypes which have not been found in the considered country. C. Haplotype networks highlighting haplotypes encountered in the two wild isolates (gray circles): black center, ROL, white center, IL.

The assumption that mutation rates are different between cytoplasmic and nuclear genomes in *D. gallinae*, resulting in more monophylies achieved in the former than in the latter due to theoretically 3-fold differences of effective population size (4-fold in diplodiploid organisms [13], but *D. gallinae* is a haplodiploid organism, [14], [15]) is confirmed by both haplotype networks and F-statistics. Indeed, the basal branch of the clade L1 is much longer in COI than in Tpm in haplotype networks (not shown, see phylogenetic topologies [17]). Furthermore, isolate differentiation is much more important based on mitochondrial COI than nuclear Tpm (cf. Fig. 1-C and below results on AMOVAs and pairwise F_ST_). Indeed, 1.4- to 32-fold higher percentages of variation are explained by among group differences in AMOVAs (Table S1), and ranges of pairwise F_ST_ are on average 2.77 times higher based on COI than on Tpm, which is expected between mitochondrial and nuclear data if migration rates are equal in males and females [8].

### Population structure: network-based haplotype genealogies

Nuclear and mitochondrial lineages defined by [17] are highlighted in Tpm and COI haplotype networks from isolates of *D. gallinae* s. str. (Fig. 1-A). In the Tpm network, a common point between the two present isolates from wild avifauna IL and ROL is the important divergence between Tpm haplotypes they contain (see Fig. 1-A). However, ROL contains only four haplotypes, whereas IL contains 22 different Tpm haplotypes, all distributed around the network (Fig. 1-C). As for COI haplotypes, ROL and IL respectively provided 2 and 4 haplotypes which are respectively monophyletic, and group together with each other and with the farm-lab isolate SK, as in the phylogenetic reconstruction published by [17].

Isolates from wild birds do not share any COI haplotype with any domestic bird. A single COI haplotype is shared between two types of domestic birds (layer and Bresse AOC chicken – see Methods S1 –, named Co_1 by [17]). In contrast, 3 of 33 non L1 haplotypes of Tpm are found to be shared between wild and domestic birds. Moreover, Tpm haplotypes found in wild avifauna are much more numerous (26) than in fowl farms (10), of which three are conspicuously dominant (named Tro_1, Tro_2, Tro_3 in Roy et al. 2010b, Fig. 1-A) and present all around the world (cf. Fig. 1-B). Three others are present only in Brazilian farms, but are likely not specific to Brazil, since one is shared with a wild bird colony sampled in Europe (The Netherlands).

Overall, the network obtained from COI haplotypes isolated in *D. gallinae* s. str. forms three different clusters, which appear to be separated by 14–22 mutated positions from each other (cf. Fig. 1-A) and respectively correspond to Lmt1, Lmt2 and Lmt3 + some haplotypes basal to Lmt3 as defined by Roy et al. [17]. Here this last lineage will be referred to as Lmt3^+^. The integration of all COI haplotypes obtained in [17] (i.e. also including some isolates^−^), as well as some haplotypes from Norway published in Genbank by Øines et al. to the network finely compliments it (less numerous median vectors) and allows finding shared haplotypes between isolates sampled in Rhone-Alpes French region (Bresse) and non French isolates (not shown). In this network, haplotype Co_17 (3 individuals sampled in a Bressan layer farm) of Roy et al. [17] turns out to be the center of a star-like phylogeny (1–2 mutations between center and branches). Co_17 is shared by most of Brasilian individuals.

### Population structure: Bayesian clustering and assignment

#### Negligible effect of LD on population structure estimates

Linkage disequilibrium was detected with a significant r value>0.5 (significance assessed following two-tailed Fisher's exact test with P>0.05) between pairs of sites involving 71 different sites in COI and 76 (both substitutions and indels considered) in Tpm (see list of sites in legend of Figure S2). Analyses resulting from datasets excluding these sites generated clustering topologies very similar to those obtained using whole datasets. The only differences are a slightly reduced number of hierarchical levels according to ΔK values in COI analyses, while no decrease in the total number of clusters and no difference in the individual assignment to clusters have been revealed (Figure S2).

**Figure 2:**
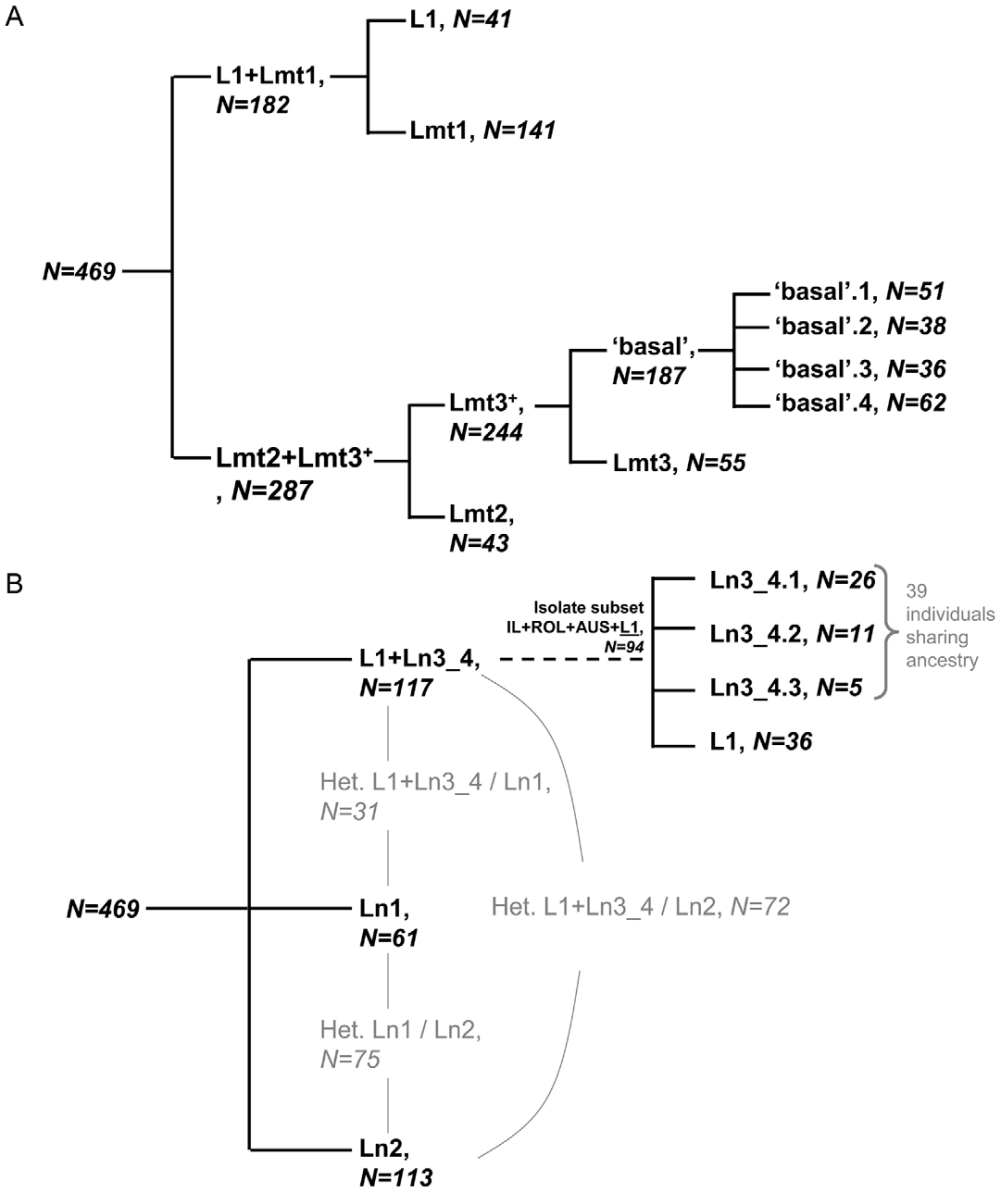
Summary of hierarchical results. A. COI. Ln assignment value threshold >0.8. Only two of the 469 individuals under test kept unassigned (<0.6, max inferred ancestry 0.54>x>0.58) (both from CON and located basally to Lmt3 in haplotype networks) B. Tpm. Black: Ln assignment value threshold >0.6 (91% individual inferred ancestry >0.9). Grey : intercluster heterozygous (mean 0.450/0.550 [0.405–0.595]).

The clustering approach used by Structure to determine the number of genetic groups within the two genes under test identified two hierarchical levels of subdivision in Tpm whole dataset as well as in Tpm dataset including only pairs of sites with a r_LD_<0.5 and four in COI whole dataset. The results of the different rounds of the process are summarized in Fig. 2.

Sorting of the obtained Tpm results by the COI lineage membership individual information as defined by [17] and conversely shows that admixture between lineages is omnipresent, at the exception of L1 (Figure S3), as expected within species. Interestingly, none of the first level subdivisions in any of the two genes present non L1 lineages as a sister to L1, although L1 appears in both cases strictly composed of a single cluster. Isolation of L1 appears at the second hierarchical level in both nuclear and mitochondrial analyses. Exclusion of pairs of sites with a r_LD_>0.5 makes it appear isolated at first level in COI analyses, with value of *K* associated with Δ*K*_max_ = 3 and the following topology: (Lmt2+Lmt3; Lmt1; L1) (see Figure S2). Note that a few admixture of L1 with wild populations at the individual level seems to have occurred in Tpm analysis when pairs of sites with a r_LD_>0.5 are excluded (see Figure S2).

#### COI: lowest within-individual admixture, 2 first-level clusters present in some farm isolates

**Figure 3:**
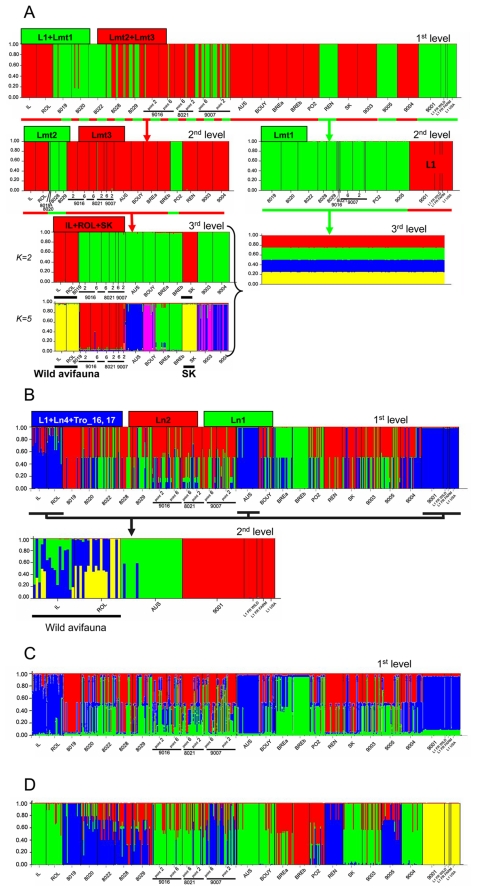
Structure Q plots. Hierarchical Structure analyses performed to determine the number of genetic groups (K) present in *D. gallinae* s. l. isolates under test using whole sequence datasets. See Table S1 for details on each isolate. A. COI whole sequence dataset. The Q plot shown for each analysis was generated using the value of K associated with ΔK_m_. Each individual included in a given analysis is represented by a vertical bar showing degree of admixture. Arrows indicate subsequent hierarchical analyses. Remark on third level *(left)*: 2 different bar plots with respectively K = 2 and K = 5 are displayed, because ΔK values calculated following Evanno et al. (2005) generated 2 sharp peaks at these two values, instead of a single one. B. Tpm whole sequence datasets, nucleotidic substitutions. C. Tpm, 8 indels, first-level analysis only. D. Combined COI and Tpm whole datasets.

**Figure 4:**
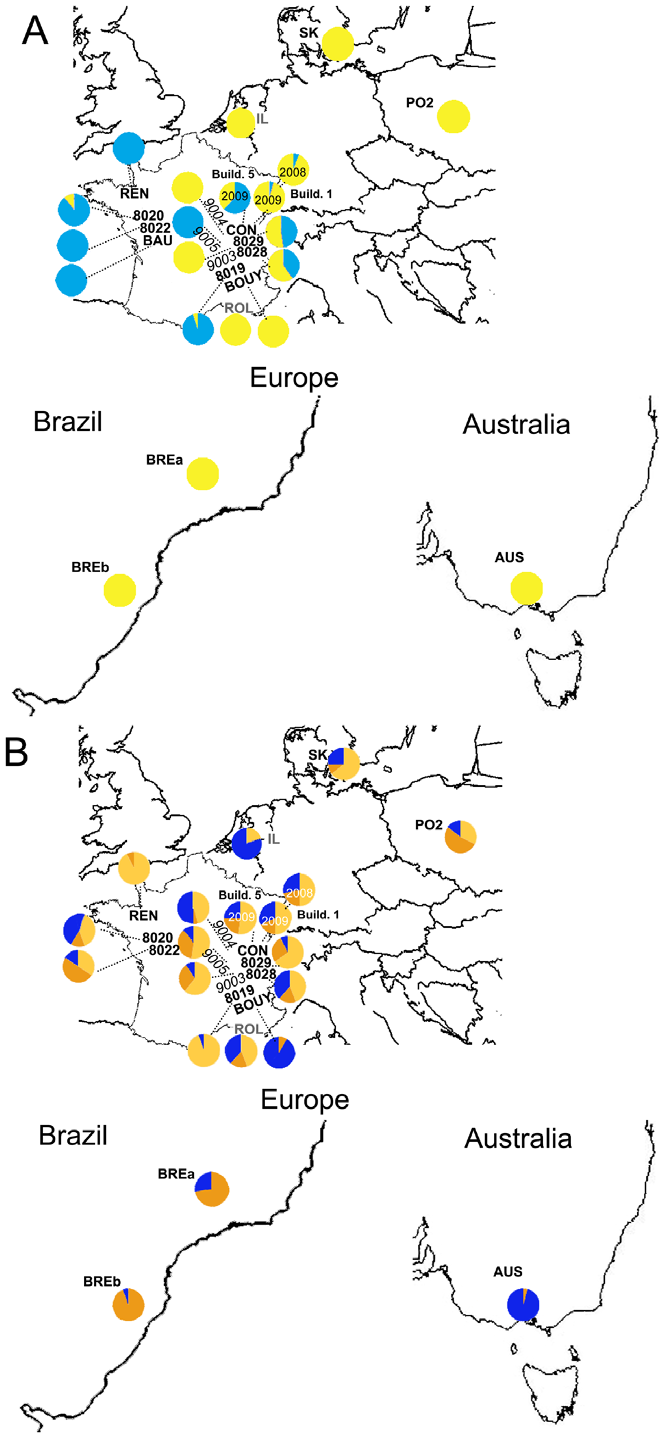
Comparison of patterns of mitochondrial and nuclear genetic variation in *D. gallinae* s. str. Clusters retained from first-level Structure results with whole sequence datasets as well as with datasets excluding pairs of sites with a significant (P>0.005) r_LD_ value >0.5 (see Figure S2). The maps depict the Europe (*top*), an eastern portion of Brazil (*bottom*, *left*) and the eastern part of Australia which includes Victoria (*bottom*, *right*). Farms CON, 8028, 8029, 9003, 9004 and 9005 are located in the French region named “Bresse”. Coloured circles represent isolates and are labeled following Table S1. Farm CON is the focused farm within which several different buildings/points/years have been sampled and correspond to isolates 8021 and 9016 (build. 1, sampling years 2008 and 2009 resp.) and 9007 (build. 5, sampling year 2009). Isolate from farm Bau has been genotyped on COI only. Labels are coloured according to the type of sampled bird: **black bold**, isolates from layer farms, *black italic*, isolates from “Poulets de Bresse” AOC broilers, **grey bold**, isolates from the wild avifauna. A. COI. Isolate circles are coloured based on the proportion of individuals assigned to either of the two mtDNA first-level clusters: Lmt1 (light blue), Lmt2–3 (yellow). Individuals assigned to Lmt1 have in most cases the mitochondrial haplotype Co_1 and in some cases have one haplotype differing from Co_1 by one or two mutated positions. B. Tpm. Isolate circles are coloured based on the proportion of individuals assigned to either of the three nDNA first-level clusters: Ln1 (dark orange), Ln2 (light orange), Ln3–4 (blue).

Assignment analyses of COI whole dataset using the admixture model revealed strongly motley populations at the isolate level, but low admixture at the individual level (values of Dirichlet parameter for the admixture proportions α≈0.02) (Fig. 3). At the three first COI hierarchical levels in analyses using the whole sequence dataset, inferred ancestry of individuals were >0.8 in almost all cases. At the fourth level only, small portions of shared ancestral genome were inferred for a few individuals. When considering mitochondrial lineages defined in [17] by phylogenetic inference, the uppermost detected structure level in COI data is composed of 2 clusters (Fig. 3), respectively corresponding to L1+Lmt1, Lmt2+Lmt3^+^ (see Fig. 1-A). Note that there is almost no individual shared ancestry between the two clusters. And yet, isolates 8019, 8020, 8022, 8028, 8029, points 2 and 6 of 9016, of 8021 and of 9007, which represent most of French layer farms distributed in both Brittany and Rhône-Alpes and including the Bresse region (see Table S1), show important within-isolate heterogeneity, with individuals assigned to either of the two first-level clusters melted together in these isolates (Fig. 4). We consider that such motley populations contain isolated lineages having recently undergone a secondary contact because almost no admixed individuals were detected, because diversity between these two clusters is similar to interspecific values (π , cf. [18]) and because COI is expected to represent a more recent picture of population structure than does Tpm. As a result, the subsequent lower levels analyses were performed on subsets strictly corresponding to individual assignment (Fig. 3-A). L1 is then completely isolated from the two other clusters. As a result, we consider that the few individual admixture observed in Tpm analysis when pairs of loci with a r_LD_>0.5 are excluded is an artifact resulting from a lack of information.

#### Tpm: 3 first-level clusters, majority inter-cluster heterozygous, different degrees of within-individual admixture wild vs farms

Tpm analyses revealed important proportion of inter-cluster heterozygous at first level of population structure, with most of shared ancestry values ≈1 (homozygous) and ≈0.5 (heterozygous) and almost no individual sharing ancestry between the three admixted lineages. Indeed, 177 inter-cluster heterozygous individuals of 469 (37.7%) were recorded. Very little admixture seem to have occurred at the individual level (α≈0.08), since almost no individual shares ancestry from the three retained clusters according to ΔK_max_ values (a single one received partitioned inferred ancestry: 0.408/0.423/0.169), even with very small ratios, as is shown by distribution of inferred ancestry values (3 groups of values sharply separated: ≈0, ≈1, ≈0.5). And yet, of the 292 individuals which do not show inter-cluster heterozygosity at the first Tpm hierarchical level, 265 were assigned with inferred ancestry >0.9, 26 were assigned with inferred ancestry comprised between 0.6 and 0.89 and a single individual partitioned (cf. above). When considering nuclear lineages defined in [17] by phylogenetic inference, the 3 retained clusters consist respectively of L1+Ln3+Ln4, Ln1, Ln2 (Fig. 1-A, 2). Of the 177 inter-cluster heterozygous individuals, 71 include haplotypes from L1+Ln3+Ln4 and Ln2, 31 from L1+Ln3+Ln4 and Ln1, 75 from Ln1 and Ln2. It is obvious that individuals from wild birds (IL and ROL) show inferred ancestry ratios far from 0.5/0.5, as opposed to farm isolates. Due to the large dominance of ½ admixed individuals at the first level in farm isolates, in order to estimate the subsequent lower structure levels, subsets were not grouped using inferred ancestry >0.6 threshold, but rather by grouping the most homogenate isolates according to bar plot observation, as in Rollins et al. [26] (Fig. 3-B). Only isolates IL, ROL, AUS, and all L1 (representative of L1+Ln3+Ln4), as well as BREa and BREb (representative of Ln1), provided enough homogenous inferred ancestries at the isolate level to be tested at the second hierarchical level. Only the first group was tested since it contained a significant number of individuals (94) distributed in different isolates, and since it included some reference isolates: *D. gallinae* L1 isolates, wild *D. gallinae* s.str. isolates IL and ROL. At this level, distribution of inferred ancestry values shows much less sharp groups than in the first level analysis (Fig. 3-B). It is noticeable that the Tpm matrix considering substitutions only (109 polymorphic loci) and the Tpm matrix composed of 8 encoded indel loci result in very similar assignments (Fig. 3-C). This confirms that both these types of DNA evolution events have evolved under similar rules (neutrality/selection).

#### Combined mito-nuclear DNA: assignments consistent with single gene analyses

Using the combined dataset, the uppermost detected structure level is composed of 4 clusters (Fig. 3-D), of which one is composed of all L1 isolates and which did not contradict single gene analyses. Results obtained based on the combined matrix will be referred to as follows: the 3 non L1 clusters will be hereafter named k2, k3 and k4, respectively. Cluster k2 includes, roughly, all individuals with Tpm haplotype in Ln1 clade in [17], k4 groups individuals with COI haplotypes in the Lmt1 clade and k3 groups the remaining non L1 individuals, including wild isolates IL and ROL. Subsequent lower structure analyses were performed on subsets defined by a threshold assignment of 0.7. They resulted in the exclusion of 207 individuals (44% genotyped individuals). Analysis of k2 (42 individuals) and k4 (63 individuals) 0.7 assigned individuals did not reveal any substructure. As for k3 (157 individuals), a 3-cluster substructure was revealed (not shown). In this substructure, one cluster appears proper to the two isolates from wild birds. The two other clusters appear admixed within most of individuals in each isolates except the Australian farm one AUS (a single cluster).

Since it has been shown that the presence of LD between sites might generate spurious clusters [11], structure analyses on combined matrix have been performed using both the admixture model and the linkage model. Thus, the latter allows taking into account potential LD by considering map distances between sites within a single sequence locus. Linkage model analyses retained r values ranging from 0.0001 to 0.0003 (with K = 4). However, the Ln variance was excessively high, making the estimation of the best K impossible, and assignment to clusters arduous (0.6<α<0.9). Additional runs were then processed with increased burn-in periods (500000 rep.) and consecutive MCMC replicates (800000), with identical results. Such difficulties are common and the linkage model does not appear to be much adequate in present case, since there is very little chance for recombination to occur in such small gene fragments of a eukaryote organism. Despite such a high Ln variance, clustering obtained with the linkage model met the ones obtained with admixture model (Figure S4) (four clusters, with L1 as an isolated entity).

### Demography: diverse histories among isolates including among the wild isolates

In the wild avifauna, mismatch distributions of COI and Tpm haplotypes are very different between IL and ROL. It significantly matched the model of sudden expansion in IL (typical unimodal distribution in both genes, Tajima's D close to zero, Figure S5). In contrast, number of COI haplotypes is strongly reduced in ROL (two close COI haplotypes of similar frequency, linked to each other by a median vector one mutation apart in MJN network; Fig. 1-C), leading to a bimodal mismatch distribution. ROL's Tpm haplotypes also show a roughly bimodal distribution of zero and high pairwise differences values.

Of 24 COI sequences isolated in SK individuals, when considering the most represented haplotype as the ancestral one, observed number of mutations in the 543-bp COI fragment in SK across ca. 52 generations (28.2–112.7) is three (distributed on three different haplotypes of which one has been recorded twice and two once) (Table 1). Among farm isolates, only AUS, REN and 8019 possess more than 1 mutations in their majority group of haplotypes, as SK (3, 3, 2 resp.). In other farm isolates, either diversity of mismatch values was lacking (one or two haplotypes, Table 1) or mismatch distribution was bimodal due to haplotypes assigned to two different COI clusters in first-level Structure analyses.

**Table 1:** Summary of DNA diversity indices.

										COI - model of sudden expansion					Tpm - model of sudden expansion				
GP	Context	π_COI_/π_Tpm_	π_COI_	π_Tpm_	K_T1_	D	Isolate/*group of isolates	S_maj_	Majority COI	COI m. d.	SSD	P(ssd)	r	P(r)	Tpm m. d.	SSD	P(ssd)	r	P(r)
1	Wild avifauna	<0.5	<0.005	>0.0150	2	Diverse D values	IL	2	Lmt2–3	uni	0.02	0.22	0.18	0.13	uni	0.01	0.84	0.01	0.14
							ROL	3	Lmt2–3	2h	0.06	0.01	0.71	0.60	bi	0.21	0.08	0.36	0.07
2	Danish, Polish, French layer/broiler farms	<0.5	<0.005	>0.0075 <0.0120	3	Diverse D values	8022	0	Lmt1	1h	0.00	0.00	0.00	0.00	bi	0.25	0.05	0.47	0.07
							9005	0	Lmt1	1h	0.00	0.00	0.00	0.00	bi	0.25	0.07	0.49	0.09
							BOUY	4	Lmt2–3	uni	0.16	0.10	0.62	0.04	bi	0.24	0.00	0.46	0.00
							PO2	2	Lmt2–3	2h	0.20	0.12	0.77	0.03	bi	0.25	0.06	0.47	0.10
							SK	3	Lmt2–3	uni	0.00	0.46	0.20	0.38	bi	0.41	0.00	0.50	0.94
3	Australian,Brazilian, French layer farms	<0.5	<0.005	>0.0015 <0.0120	2	D_COI_ and D_Tpm_<0	AUS	3	Lmt2–3	bi	0.02	0.19	0.50	0.56	2h	0.01	0.07	0.86	0.81
							BREa	0	Lmt2–3	1h	0.00	0.00	0.00	0.00	bi	0.15	0.06	0.48	0.43
							BREb	1	Lmt2–3	2h	0.00	0.36	0.62	0.76	bi	0.02	0.14	0.64	0.70
							REN	3	Lmt1	uni	0.00	0.48	0.19	0.38	2h	0.03	0.06	0.78	0.75
4	French layer farm	>1.0	0.005	0.0016	2	D_COI_ and D_Tpm_<0	8019	2	Lmt1	bi	0.01	0.22	0.16	0.52	2h	0.01	0.04	0.83	0.80
5	French layer/broiler farms	>0.5 <1.0	>0.005 <0.020	>0.0075 <0.0120	3	Diverse D_COI_; D_Tpm_>0	8020	1	Lmt1	bi	0.04	0.08	0.19	0.42	bi	0.25	0.00	0.47	0.00
							8021	0	Lmt2–3	bi	0.03	0.08	0.71	0.66	bi	0.14	0.01	0.25	0.00
							8028	-	-	bi	0.49	0.00	0.52	0.94	bi	0.40	0.00	0.52	0.94
							8029	-	-	bi	0.35	0.00	0.70	0.00	bi	0.24	0.00	0.39	0.00
							9003	-	-	2h	0.49	0.00	0.75	0.92	bi	0.47	0.00	0.52	0.96
							9004	-	-	2h	0.26	0.01	0.70	0.23	bi	0.53	0.00	0.64	0.94
							9007	0	Lmt1	bi	0.28	0.00	0.61	0.17	bi	0.18	0.01	0.31	0.00
							9016	1	Lmt2–3	bi	0.02	0.16	0.62	0.65	bi	0.14	0.00	0.27	0.00
6	Pigeon breeding facility	NA	0.003	0.0000	1	D_COI_<0	9001	0	L1	2h	0.07	0.02	0.70	0.67	1h	0.00	0.00	0.00	0.00
var.	Pigeons	-	-	-	-	-	* All L1	-	-		0.14	0.04	0.28	0.01		0.06	0.18	0.25	0.18
	Wild avifauna	-	-	-	-	-	* All wild avifauna	-	-		0.08	0.06	0.09	0.16		0.02	0.00	0.03	0.00
	French layer/broiler farms	-	-	-	-	-	* All French layer/broiler farms	-	-		0.11	0.00	0.16	0.00		0.20	0.00	0.34	0.00
	Layer/broiler farms	-	-	-	-	-	* Cluster k2	-	-		0.12	0.12	0.27	0.16		0.00	0.12	0.91	0.91
	Layer/broiler farms+wild avifauna	-	-	-	-	-	* Cluster k3	-	-		0.01	0.26	0.03	0.26		0.15	0.00	0.25	0.00
	Layer/broiler farms	-	-	-	-	-	* Cluster k4	-	-		0.00	0.56	0.26	0.56		0.01	0.10	0.81	0.78

Genetic profiles (GP), as delineated from observation of COI and Tpm diversities and grouping together different isolates (left part, top), draw outlines of five different scenarios in the demographic history of *D. gallinae* s. str and one of *D. gallinae* L1. Majority and 1–2 steps derived COI haplotypes represent a group of haplotypes whose frequency within a given isolate is >60% and which forms a less than 3-steps star-like genealogy. Results of tests for demographic expansion based on comparison of the observed distribution of pairwise nucleotide differences among haplotypes (mismatch distribution) to the distribution expected from a sudden-expansion model (Arlequin 3.11) are provided per isolate and per some groups of individuals (right part and bottom). S_maj_ refers the number of segregating sites detected in the majority and 1–2 steps derived COI haplotypes of the isolate under consideration, K_T1_ to the number of 1st-level Tpm Structure clusters, D to Tajima's D value, Majority COI to mitochondrial lineage which the majority COI haplotype belongs to, COI m.d. or Tpm m.d. to the profile of mismatch distribution in COI or Tpm dataset (notation: uni = unimodal; bi = bimodal; 2h = 2 haplotypes present only; 1h = a single haplotype present only), SSD to the sum of squared deviations (P_[simulated SSD≥observed SSD]_), r to the Harpending's raggedness index.

As for Tpm mismatch distribution, it was bimodal in SK as in ROL and in most farm isolates (Table 1), with the first peak of the bimodal mismatch distribution characterized by zero values caused by recurrent haplotypes, while the second peak was dominated by divergence between distant haplotypes.

Despite numerous Tpm bimodal profiles, mismatch distributions of COI and Tpm fit the model of sudden expansion in seven and two of the 13 French farm isolates respectively. In contrast, the demographic imprint from mismatch distributions shows old stationary populations of farm isolates at France level (all French farm isolates pooled together, SSD 0.108 in COI and 0.204 in Tpm). Lastly, mismatch distributions of COI and Tpm haplotypes in cluster k4 (combined Structure analysis) are unimodal and fit well the model of sudden expansion (COI: SSD 0.002, raggedness index 0.258 with p = 0.564, Tpm: SSD 0.011, raggedness index 0.808 with p = 0.780).

### Population differentiation

#### AMOVAs: no structure except wild vs domestic and geographical location at the world level; less within individual differences with assignment threshold >0.7

In hierarchical Tpm AMOVAs, F_IS_ values were not significant in any analysis except with the geographical and habitat criteria (0.067, P<0.05). F_IS_ calculated per inferred Structure cluster on individuals assigned by >0.7 in Structure, provided insignificant values for k2 and k4, whereas k3 and L1 had significantly positive values (0.39 and 0.89 respectively, P<0.05). The Tpm AMOVAs indicated that a large fraction of the total variation in every analysis could be explained by differences within individuals (mean 55.20%), with the maximum percentage values found in analyses involving French fowl only (86.28–86.76%). In contrast, minimum percentage values explaining variations by differences within individuals were obtained in analyses involving groups defined by clustering assignment with a threshold >0.7 (first level of combined analysis with Structure, 30.34% for *D. gallinae* s. str., 27.47% for *D. gallinae* s. l.).

AMOVAs using bird type as the group criterion for farm isolates (layer/Bresse AOC broiler) detect by far larger amounts of variation which could be explained by differences within individuals (Tpm 71.89–86.28%) and among isolates within groups (Tpm 12.46–29.73%, COI 72.03–86.31%) than among groups (negative values in both Tpm and COI analyses). When using habitat as the group criterion (wild/domestic), 80.15% to 94.46% of the total Tpm variation in each analysis is more or less equally split into variations explained by within individual differences and among groups differences, whereas dominant COI variation (49.48–62.62%) could be explained by among isolates differences. When considering the geographical location as a group criterion in farm isolates, among groups differences could explain 49.54% of the total Tpm variation and 96.42% of the total COI variation at the world level (excluding France), as opposed to 0% of the total Tpm variation and only 30.13% of the total COI variation within France. When considering clusters inferred by Structure analyses as a group criterion, the proportions of variation explained by both within-individual and among-groups differences are somewhat different than with above group criteria. Indeed, higher values are attributed to the latter (54.48–58.49% of the total variation) and reduced values for the former (27.35–37.71%).

#### Pairwise F_ST_: high discordance geographical location vs genetic differentiation, higher differentiation between buildings than between successive flocks

Tpm pairwise F_ST_ are much lower than COI pairwise F_ST_ (average 0.31 vs 0.69). Between L1 and non L1 isolates, Tpm pairwise F_ST_ values are >0.42 except against the wild isolate IL (0.27–0.45) and COI pairwise F_ST_ values are all >0.80 (P>0.00001 for all values). Wild isolates ROL and IL provide rather high Tpm F_ST_ values when compared to farm isolates (>0.41 and >0.28 respectively, P>0.00001), except for IL vs 9004 (0.184) and IL vs BOUY (0.178) (P>0.00001 for both values). ROL's and IL's COI pairwise F_ST_ are all >0.53 (average 0.83). When comparing ROL and IL to each other, Tpm F_ST_ value is much lower (0.19, P>0.00001) and COI higher (0.90, P>0.00001). When comparing Australian and Brazilian farm isolates with European ones, Tpm and COI pairwise F_ST_ values are rather high (>0.5 and >0.75 respectively, P<0.00001) except against some isolates from the Bresse region (Rhône-Alpes, France) (>0.10 and >0.37 respectively, P<0.00001) and except Tpm pairwise F_ST_ alone between French isolate 8022 (Brittany) and Polish isolate PO2 (0.11 with P<0.05, 0.10 with P<0.00001 respectively). Tpm pairwise F_ST_ of the lab isolate SK are comprised between 0.02 and 0.19 when compared with European farm isolates and between 0.26 and 0.48 when compared with wild isolates (P>0.00001 for all values). All COI values for pairs involving SK are >0.59 (P>0.00001).

Overall, pairwise F_ST_ among French farm isolates are highly diverse, with Tpm ranging from 0 to 0.43 and COI from 0 to 0.98. In regard to correlation of geographic distance with genetic differentiation, high discordance is apparent in these results. Indeed, some Tpm values are close to zero (some negative) between isolates from a single farm (CON, isolates 8021 and 9016), but also between farms located at various distances apart (<10 to >700 km) (9003 and CON, −0.02–0.01, 15 km; 8022 and 8029, −0.02, 583 km, REN and 8019, 0.017, 835 km…). In contrast, some higher F_ST_ values are found between very close farms, within a single fowl industry (e.g. 9004 and 9005, 0.24, <5 km, Bresse broilers, P<0.00001).

Some very low COI F_ST_ values are found in some cases between distant farms within a given country (<0.008, e.g. France: 8020 and 8019, 0, 693 km; REN and 8019, 0.007, 693 km; Brazil: BREa and BREb, 0.006, 1157 km by road). As for COI F_ST_ between isolates from the single farm CON, they are particularly informative: the different samples from two points within a single building (8021, 9016, 9007, points 2 and 6 in each) as well as samples from a single building on two successive flocks (8021 and 9016) provide F_ST_ value close to 0 (−0.06–0.05), whereas samples from the two different buildings (build. 1: 9016, build. 5: 9007; distant by 78 m from each other, 150 m by feet for humans) under test provided high F_ST_ values (0.42–0.69, P<0.05) despite staff's comings and goings.

#### High ratio COI π/Tpm π in 7 French farm isolates

The ratio of COI π and Tpm π in French farm isolates 8019, 8020, 8028, 8029, 9016, 8021, 9007, 9003 and 9004 is amazingly higher (>0.5) than in other isolates (Figure S6).

## Discussion

Our results have helped conclude that: (1) isolated *D. gallinae* L1 and several mutually interadmixed lineages have radiated at the same moment, before hybridization, (2) extensive gene flows have recently occurred between distant farms, including at the intercontinental level, (3) gene flows are very reduced, if not null, between wild and domestic birds in Europe (and likely in Australia), (4) mite populations sampled in Brazilian layer farms seem to result from multiple introduction events, including both wild and domestic origins and (5) mite populations from French farms result from very recent secondary contact between isolated populations, through a NW-SE axis.

This is important because it shows that populations belonging to *D. gallinae* s. str. are particularly prone to strong genetic differentiation without reaching any intrinsic hybrid incompatibility, in contrast to some other arthropods (e.g. *Drosophila pseudoobscura* [27]), and that those that are infesting fowl farms have recurrently hybridized. This means that *D. gallinae* s. str. is expected to possess nowadays a diversity of genetic architectures, which may be involved in its capacity to rapidly adapt to a diversity of selective pressures and colonize fowl farms. Moreover, trade, but not birds, seems to be the routes that is the most often used by mites to disseminate, clearing avifauna of participation to farm infestations at least in Europe and bringing useful information for potential prevention.

On a methodological point of view, inclusion/exclusion of pairs of sites with a r_LD_<0.5 resulted in negligible changes (a little bit less resolved 3^rd^-level in Tpm, one hierarchical level less in COI when excluded), which did not modify the overall clustering, nor the individual assignment to clusters. Therefore, results obtained in the present Structure analyses are deemed reliable.

### *D. gallinae* s. l.: a history of radiation followed by hybridization of all resulting lineages but one (L1)

The two following topologies may be inferred from the first two levels of structure analyses using both single genes: nuclear ((L1,Ln3_4),(Ln1,Ln2)) and mitochondrial ((L1,Lmt1),(Lmt3^+^,Lmt2)) (Fig. 2). Inter-cluster recurrent heterozygosities in Tpm structure analyses represent important clues in favour of nearly interspecific hybridization. Long-term asexual reproduction would be an alternative cause for increased heterozygosity over time, the two alleles at a locus evolving independently and accumulating different mutations, a phenomenon known as the Meselson effect [28]. Nevertheless, [14] and [15] not only have shown that *D. gallinae* was haplodiploid, that is parthenogenesis is strictly arrhenotokous, but also they have proven that mating was necessary to induce egg development, including unfertilized male eggs. Therefore, it appears very unlikely that any asexual lineage might have developed in *D. gallinae* complex. As a result, the high level of heterozygosity and the important divergence between Tpm alleles is not attributable to asexual reproduction. Along with inter-cluster recurrent heterozygosities in Tpm structure analyses, the fact that separation of L1 only appears either at the second hierarchical level (all analyses using Tpm sequences, COI whole sequence dataset) or at the same time as two different clusters of *D. gallinae* s. str. (COI dataset excluding pairs of sites with a r_LD_>0.5) strongly suggests that non L1 populations are resulting from hybridization between long isolated lineages with same level of structuration as L1. The reduced percentage explaining variations by differences within individuals in AMOVAs involving groups defined by clustering assignment suggests that an important fraction of inter-cluster heterozygous individuals have been excluded by removal of individuals receiving <0.7 inferred ancestry in combined analyses and is one more clue in favor of nearly interspecific hybridization. Since L1 is the only currently isolated lineage [20], one may assume that Ln3_4 and Lmt1 could have represented formerly an incipient species which has undergone a speciation reversal as a result of nearly interspecific hybridizations as may happen in case of habitat defragmentation according to Seehausen et al. [5]. The analyses with datasets excluding pairs of sites with a r_LD_>0.5 do not contradict such a scenario, since the first level is omitted in COI only and results in divergent Lmt1 clade: (L1,Lmt1,Lmt3^+^+Lmt2). Neither do the results obtained *via* the combined two-level analysis. Overall, entities which nuclear lineages Ln3_4, Ln1 and Ln2 and mitochondrial lineages Lmt1, Lmt2 and Lmt3^+^ belong to seem to have hybridized between each other, whereas L1 remained isolated. It is difficult to conclude without any doubt whether Ln1 and Ln2 did represent two different lineages or whether they already resulted from incomplete lineage sorting. Nevertheless, strong supports for two different clusters in the first level analysis of the Tpm alone matrix tend to confirm their isolation at the moment just before hybridization. As for the reason why L1 kept isolated, we assume that it may have developed any intrinsic incompatibility (male choice, incompatible interactions between loci, sperm precedence, …) unlike other co-incipient species or the ecology of its bird host (pigeons) associated with the strict need of nest/roost sharing to get able to switch from one host to another already noticed in *Dermanyssus* species [21] preserved it from switching to any other host.

In short, evidence for admixture between all lineages but *D. gallinae* L1 and separation of *D. gallinae* L1 and three different lineages at the same point strongly suggests a radiation has generated at least four incipient species, then three of them have undergone hybridization, leading to a reversal of their respective speciation.

### Multiple and diversely intense bottlenecks, depending on their causes

In the wild avifauna, the marked difference of demographic characteristics between IL and ROL isolates may be explained by the ecology of their respective hosts (Common Starlings and European Rollers respectively): Common Starlings are known to be either resident or partly migrating with some populations staying the whole year some others going westward to winter. In all cases, some wintering colonies stay in the premises, which might have allowed mites to continue developing through the year and have preserved it from drastic seasonal bottlenecks. European Rollers are migrating to South Sahara from autumn to spring, which results in the absence of host on the premises for micropredator mites during several months per year, and is likely to be responsible of cyclically repeated bottlenecks.

In farms, the diversity of demographic imprints reported in this study was expectable due to the various known causes for bottlenecks in farms and the known ability of *D. gallinae* s. str. to rapidly reach high densities within short time frames (Methods S1). As a consequence of the repeated and non synchronous nature of such demographic events in this species in farms, fluctuations in the time since bottleneck as well as in the size of founders are consistent with the diversity of mismatch distributions, the variable haplotype diversity and the various observed Tajima D patterns. Among them, the contrasted mito-nuclear Tajima D pattern found in 8020, 8022, 8021, 9016 and SK matches the discordant pattern witnessing of different imprints between genes of different effective population sizes (mitochondrial DNA: D- ; nuclear DNA: D+) noticed by Fay and Wu [29] when considering mt and nDNA between generations no 1500 and 4000 following a bottleneck in human populations undergoing a founder event.

Not only cases for bottlenecks are diverse, but also their effect on the founding population size may strongly vary. Indeed, pairwise F_ST_ between farm CON isolates 8021 and 9016 (same building, two different successive flocks) indicate that both isolates belong to a panmictic population, which highlights that cleaning/disinfection actions may have almost no impact on population's genetic diversity. This was expected, as numerous appropriate hiding-places are available for mites in most farm buildings (wall cracks and crevices, link-up point between joint parts, hollow parts, etc…) and may protect more or less numerous and large mite aggregates from any pesticide spray despite careful application and even, in some cases, partial dismantling. As a result, pesticide applications during flocks may have various effects, depending on the farming structure and on the mean of application.

### Commercial exchanges are widely involved in the spread of the Poultry Red Mite in farms

#### Wild and farm populations are very distant from each other in Europe and Australia, closer in Brazil

Populations sampled in the wild avifauna (ROL and IL) appear to be genetically isolated from farm populations in Europe. First, nuclear haplotypes they possess differ from farm mites' (higher nucleotid diversity, only two from three first-level clusters present). This might result from either an increased genetic drift post-hybridization, or the occurrence of two different events of hybridization, of which only one would have occurred in wild populations under test. In the latter case, a bottleneck event in farm populations would explain the lower Tpm diversity, compared to wild populations. The effective population size of populations developing in the wild avifauna is very likely reduced as compared to farm populations (see Methods S1). This might allow genetic drift to go almost as fast as in farm populations (see Methods S1-C online). However, given the much higher number of generations in farms (ca. 50 vs ca. 10, see Methods S1-C), it is very unlikely that it goes faster in the wild avifauna, and certainly not faster enough to lead to homogenization of so different Tpm haplotypes. As a result, the former hypothesis is not supported. The latter hypothesis is consistent with the 1^st^-level Tpm structure.

Second, rather high pairwise F_ST_ in both genes and absence of shared COI haplotypes with regard to farm populations, as well as AMOVAs results based on the habitat criterion, show that populations represented by isolates ROL and IL, although genetically close to each other despite geographical distance, are individually isolated from other populations anywhere in countries under test, except in Brazil.

Concerning exchanges between wild and domestic birds, Brazilian farms provided a strikingly different pattern than European and Australian ones. Brazilian isolates BREa and BREb were the only ones which possessed some uncommon Tpm haplotypes, rather distant from typical farm haplotypes and closer to wild haplotypes. They were also the only ones generating significantly positive F_IS_ values in the present study, which highlights a deficiency of heterozygotes and may witness of the Wahlund effect. This suggests that several different points of introduction are at the origin of the dramatic colonization in Brazilian layer farms during the 20^th^ century [22], of which one might have originated rather recently from the wild avifauna. In French farms, only birds used to nest in the ceiling of buildings have been observed during our sampling activities (tits, swallows, wrens) and no *D. gallinae* s. str. have ever been found in wild bird nests in such conditions in [20] despite actual infestation by *D. gallinae* s. str. of fowl located just under. It is not impossible that some Brazilian wild birds hosting *D. gallinae* s. str. are able to nest within hens' litter, and have this way allowed a host switch. Whatever is the cause of mite switch from wild to domestic birds in Brazilian farms, such an introduction may have allowed hybridizations between long isolated lineages and generated some particular genetic architecture, a phenomenon which has been noted in invasive species [4].

#### In France, farm populations take advantage of fowl transportation to disseminate

Among French farms, the transfer of spent hens through France seems to be linked to secondary contact between divergent lineages. Indeed, nine isolates sampled in Brittany or Rhône-Alpes gather individuals which are assigned to different first-level Structure clusters with assignment values >0.8 together and show highest ratios COI π/Tpm π (Figure S5), which is strongly suggestive of recent immigration. The demographic imprint from mismatch distributions corroborates this, as it shows old stationary populations of farm isolates at France level (Table 1), while it fits well the model of sudden expansion in the Lmt1 only cluster k4 obtained through combined COI and Tpm analysis. In the France level analysis, significant raggedness indexes (0.160 in COI and 0.343 in Tpm) are consistent with the reduced gene flows between some demes [30], as has been stated in present study based on pairwise F_ST_. This highlights that sudden expansions have occurred independently in individual farms, not at the country level. Because the frequency of Lmt1 is highest in Brittany, because it is present in French farms only and was found in the presence of the other first-level COI cluster in several isolates located in Rhône-Alpes, the demographic expansion inferred from k4 DNA sequences is consistent with a history of recent range expansion from Brittany to Rhône-Alpes. This strongly supports a scenario of farm networks initially regionally fragmented in France, then defragmented consecutively to the known extension of the slaughter network through the whole country (see Methods S1 online).

#### Living vectors on shortest distances (hens), inanimate vectors on longer distances (cages)

Since no structure may be explained by the two different fowl industries under test (cluster assignment and AMOVAs) and since, in some cases, same trucks may be used for the transportation of these different strains (a farmer, pers. comm.), *D. gallinae* mite populations appear to commonly use human vehicles as a spreading mean at high geographical scales (dozens to hundreds kilometers). At lower scales, the striking difference between within-building and among-building F_ST_ values shows that mite's intrinsic mobility is not sufficient for it to circulate over several dozens meters. Within-building panmixy (COI pairwise F_ST_ within farm CON) is consistent with short time duration of mite blood meal and with the inability of mites to stay on host which should be deduced from some published data [31]. It leads to establish hens as vectors on short distances (mite climbs its host on one point, rapidly feeds on blood, then quickly goes down, most probably at any different point due to hen movement). Between-building high population differentiation shows that, outside meals, mites need to use some other vector, different from man (which regularly comes and goes from one building to another one) and likely non living as do other nidicolous micropredators such as bedbugs [32]. Because, at least in France, cages commonly run in and out of the slaughter processing plants and layer farms, our results suggest that trucks are long distance vehicles and cages are specific vectors for mites during fowl transportation. This is consistent with the need of nest sharing for switching from one host to another noted by Roy et al. [21]. Cages dedicated to fowl transportation play the role of nests and are mobile.

#### Intercontinental mite exchanges in farms originate from Europe

At the inter-continent scale, insights for exchanges also arise from present data: Lmt2 and Lmt3 are almost absent from Brittany, but omnipresent in isolates sampled in countries other than France, including Australia and Brazil. And yet, in farms, a Tpm haplotype different than Tro_1, Tro_2, and Tro_3 is present only in some Brazilian and French farms located in Rhone-Alpes (Bresse region, Fig. 1-B). This shared rare Tpm allele along with the occurrence of a shared COI haplotype between Rhone-Alpine and Australian farms suggest that there might have been some gene flows between these distant places, which is not contradictory with known trade flows between Europe and Brazil [33], [34] and between Europe and Australia [35]. However, no specific analysis has explored at which exact moment it may have happened or which vector(s) could have been responsible for such exchanges, so that they remain to be identified. Nonetheless, the fact that cleaning/disinfection actions may have almost no effect on mite populations along with observed gene flows through hen transfer leads to assume that even drastic importation programs, including quarantine and other sanitary actions as described by Scott et al. [35] in Australia for instance are unable to stop mites from swarming. At best, they may decrease the number of founding individuals allowed to enter a new farm and possibly a new country, which might explain the much reduced diversity of COI and Tpm haplotypes encountered in isolate AUS.

### Summarized demographic scenarios inferred in present study

From present DNA sequence data analyses, available ecological and trade information, six different profiles of population may be distinguished among populations of *D. gallinae* (Table 1). Profile 6 (L1) represents the original condition of hybridized lineages in *D. gallinae* s. str. as it was before hybridization, i.e. weakest sequence diversity in both mt and nDNA, similar to other *Dermanyssus* species. Profiles 1–5 concern *D. gallinae s. str.* and are consistent with 5 different histories. While profile 1 represents the wild condition, likely resulting from two originally hybridized lineages, profiles 2–5 are found in farms, and likely all result from hybridization of three different lineages. On the sequence diversity point of view, profiles 2 and 3 are the closest to profile 1 although found in farms: their COI sequence diversity is comparable to that of the wild, with 1–2 steps starlike genealogies. This suggests an expansion is in process in each, following one of the very frequently experienced bottlenecks in both wild and farm populations of *D. gallinae* s. str. Profiles 4 and 5 show substantially high COI π without any sharp increase in Tpm π which is an insight of recent secondary contact between long separated populations. Moreover, profiles 3 and 4 have much reduced Tpm diversities, which should result from strongest bottleneck. Given that three of the five concerned isolates were sampled far from Europe, this may be the consequence of long distance transportation as a very reduced number of founder individuals allowed to be imported through fowl trade.

### Conclusions and perspectives

In conclusion, the role of trade flows in mite dispersal appears striking, while wild avifauna does not seem to play any role in Europe. Cages which are carried by trucks during transfer of spent hens from farm to slaughterhouse are clearly involved as vectors in mite dispersal within France. At the international level, vectors and vehicles for mite exchanges remain to be identified. Moreover, as opposed to European and Australian patterns, some multiple introduction events from both wild and domestic birds seem to have occurred in Brazil. This may explain the rapid colonization of layer farms by *D. gallinae* in this country during the 20^th^ century.

Although we are aware that a study involving a higher number of both mitochondrial and nuclear sequences would probably help getting an even clearer picture, we are confident that not only these results will help improving prophylactic actions in the layer industry, but also that it will constitute a case in point for investigation of pest population structure using mito-nuclear sequence data. Furthermore, combination of present data with faster evolving markers such as microsatellites would be a highly interesting perspective in order to obtain a comprehensive overview of the evolutionary history of *D. gallinae* s. str. In addition, some experiments aiming at testing the presence or not of intrinsic incompatibility between *D. gallinae* s. str. and *D. gallinae* L1 would bring valuable information.

## Supporting Information

Figure S1 **Focused farm CON: location of the samples points.** Farm CON, Confrançon (Ain, France). Eight buildings containing on-ground layers. 5000 hens per building. Mite samples: 6 points have been sampled per building in buildings n°1 and 5 as explained at the bottom. Mites from points n°2 and 6 were sequenced at both COI and Tpm.
(TIF)

Figure S2 **Structure analyses excluding pairs of sites with a r_LD_ value>0.5, r_LD_ value.** Analyses have been performed based on alignments excluding following pairs of sites, which show polymorphism non-randomly associated with polymorphism of one or more other sites. A pair of sites is considered non-randomly associated to each another when its r_LD_ is significant according to the two-tailed Fisher's exact test (with P>0.005) and is above 0.5 (DnaSP 5.0). List of retained sites (the numbering of each site is based on DNA alignments available from Genbank accession numbers (Table S1) as a popset): COI: 9, 16, 18, 19, 21, 24, 30, 33, 52, 54, 57, 63, 66, 78, 81, 93, 108, 117, 123, 128, 130, 132, 144, 147, 151, 156, 171, 172, 186, 195, 210, 223, 231, 261, 267, 273, 286, 288, 291, 327, 342, 348, 351, 363, 366, 384, 393, 407, 414, 417, 420, 422, 426, 432, 453, 445, 462, 465, 468, 474, 483, 487, 501, 504, 507, 519, 522, 525, 528, 540, 543. Tpm: 9, 28, 30, 40, 106, 111, 115, 125, 141, 142, 144, 146, 152, 169, 186, 218, 228, 235, 236, 251, 254, 257, 259, 271, 274, 279, 280, 281, 289, 303, 308, 311, 316, 319, 330, 335, 343, 347, 357, 361, 363, 364, 366, 372, 377, 385, 388, 418, 421, 441, 442, 463, 464, 490, 497, 500, 506, 533, 536, 550, 581, 582, 586, 591, 592, 595, 596, 617, 622, 623, 637, 650, 667, 686, 703, 722. A. COI partial sequence dataset, excluding all pairs of sites with a r_LD_>0.5 (21 included loci). First level. *D. gallinae* s. l. K = 3. Green = L1; blue = Lmt1 (Co_1 among others) ; red, Lmt2+Lmt3^+^. Second level, left. Idem with red 2nd level subset, K = 4. Green, Lmt2 ; other, Lmt3^+^. Right, Idem with blue subset. B. Tpm partial sequence dataset, excluding all pairs of sites with a r_LD_>0.5 (113 included loci, substitutions and indels), Top and center. *D. gallinae* s. l. K = 4 and K = 3 (ΔK : two similar peaks). Red/green = L1+Ln4 (Tro_1 among others) and Ln3 (wild only) ; green/red = Ln2 (Tro_2 among others) ; yellow/blue = Ln1 (Tro_3 among others). Bottom. Idem on a subset roughly corresponding to individuals mainly assigned to the blue cluster with K = 4.
(TIF)

Figure S3 **Structure Q plots results sorted out by mitochondrial/nuclear lineage membership.** A. Tpm Q plots sorted out by the COI lineage membership individual information B. COI Q plots sorted out by the Tpm lineage membership individual information.
(TIF)

Figure S4 **Structure Q plots representing COI and Tpm first-level analyses as inferred using the linkage model.** Combined COI and Tpm whole sequence datasets, K = 4. Linkage has been evaluated based on mapped distances between sites.
(TIF)

Figure S5 **Histogram representing obtained Tajima D values (Arlequin).** Black and grey stars indicate significant D values (P<0.05).
(TIF)

Figure S6 **Nucleotidic diversities.** Values of π in each isolate as calculated using DnaSP following Nei (1987; equation 10.5). A. Absolute values. B. Ratio π COI/π Tpm.
(TIF)

Table S1 **Information on mite isolates including Genbank accession numbers.**
(DOC)

Methods S1 **Hypotheses under test and population genetics analyses.**
(DOC)
