# Supplementary figures and images for: Using Mitochondrial and Nuclear Sequence Data for Disentangling Population Structure in Complex Pest Species: A Case Study with Dermanyssus gallinae
Source: PLoS One. 2011 Jul 25;6(7):e22305. doi: 10.1371/journal.pone.0022305 (PMC3143145; doi:10.1371/journal.pone.0022305)

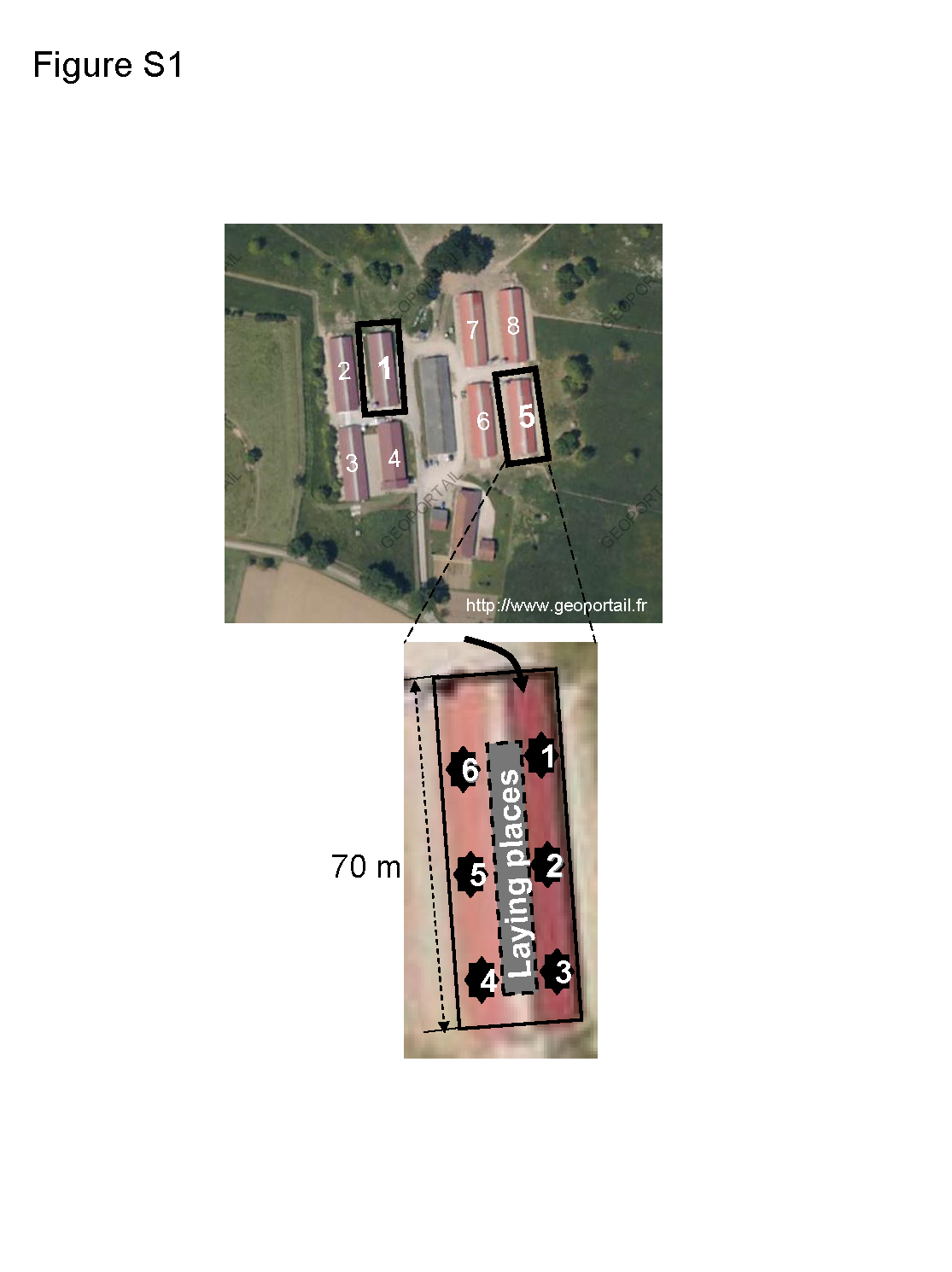

Supplement: Figure S1 — Focused farm CON: location of the samples points. Farm CON, Confrançon (Ain, France). Eight buildings containing on-ground layers. 5000 hens per building. Mite samples: 6 points have been sampled per building in buildings n°1 and 5 as explained at the bottom. Mites from points n°2 and 6 were sequenced at both COI and Tpm. (TIF) [file pone.0022305.s001.tif]

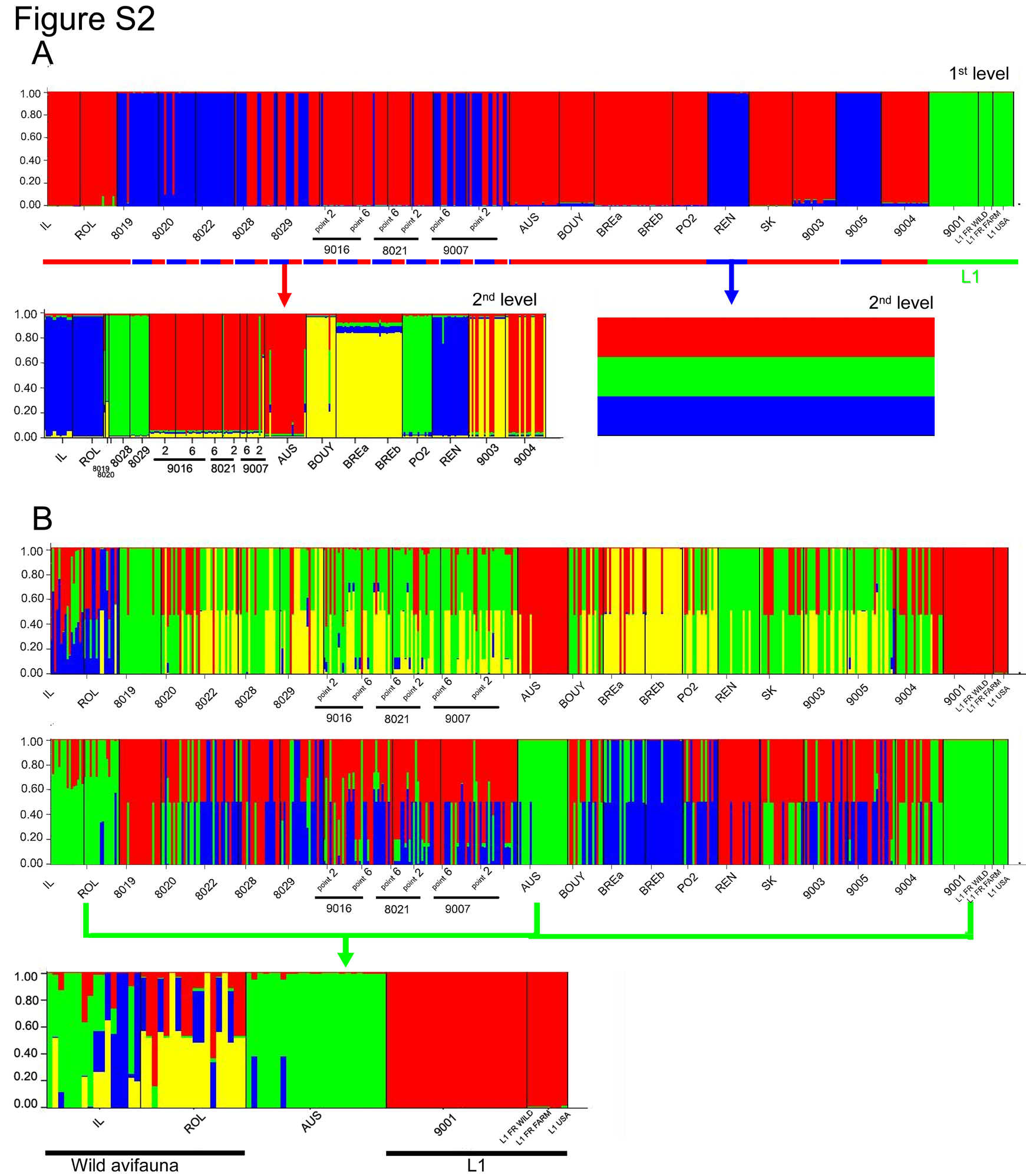

Supplement: Figure S2 — Structure analyses excluding pairs of sites with a rLD value>0.5, rLD value. Analyses have been performed based on alignments excluding following pairs of sites, which show polymorphism non-randomly associated with polymorphism of one or more other sites. A pair of sites is considered non-randomly associated to each another when its rLD is significant according to the two-tailed Fisher's exact test (with P>0.005) and is above 0.5 (DnaSP 5.0). List of retained sites (the numbering of each site is based on DNA alignments available from Genbank accession numbers (Table S1) as a popset): COI: 9, 16, 18, 19, 21, 24, 30, 33, 52, 54, 57, 63, 66, 78, 81, 93, 108, 117, 123, 128, 130, 132, 144, 147, 151, 156, 171, 172, 186, 195, 210, 223, 231, 261, 267, 273, 286, 288, 291, 327, 342, 348, 351, 363, 366, 384, 393, 407, 414, 417, 420, 422, 426, 432, 453, 445, 462, 465, 468, 474, 483, 487, 501, 504, 507, 519, 522, 525, 528, 540, 543. Tpm: 9, 28, 30, 40, 106, 111, 115, 125, 141, 142, 144, 146, 152, 169, 186, 218, 228, 235, 236, 251, 254, 257, 259, 271, 274, 279, 280, 281, 289, 303, 308, 311, 316, 319, 330, 335, 343, 347, 357, 361, 363, 364, 366, 372, 377, 385, 388, 418, 421, 441, 442, 463, 464, 490, 497, 500, 506, 533, 536, 550, 581, 582, 586, 591, 592, 595, 596, 617, 622, 623, 637, 650, 667, 686, 703, 722. A. COI partial sequence dataset, excluding all pairs of sites with a rLD>0.5 (21 included loci). First level. D. gallinae s. l. K = 3. Green = L1; blue = Lmt1 (Co_1 among others) ; red, Lmt2+Lmt3+. Second level, left. Idem with red 2nd level subset, K = 4. Green, Lmt2 ; other, Lmt3+. Right, Idem with blue subset. B. Tpm partial sequence dataset, excluding all pairs of sites with a rLD>0.5 (113 included loci, substitutions and indels), Top and center. D. gallinae s. l. K = 4 and K = 3 (ΔK : two similar peaks). Red/green = L1+Ln4 (Tro_1 among others) and Ln3 (wild only) ; green/red = Ln2 (Tro_2 among others) ; yellow/blue = Ln1 (Tro_3 among others). Bottom. Idem on a subset rough [file pone.0022305.s002.tif]

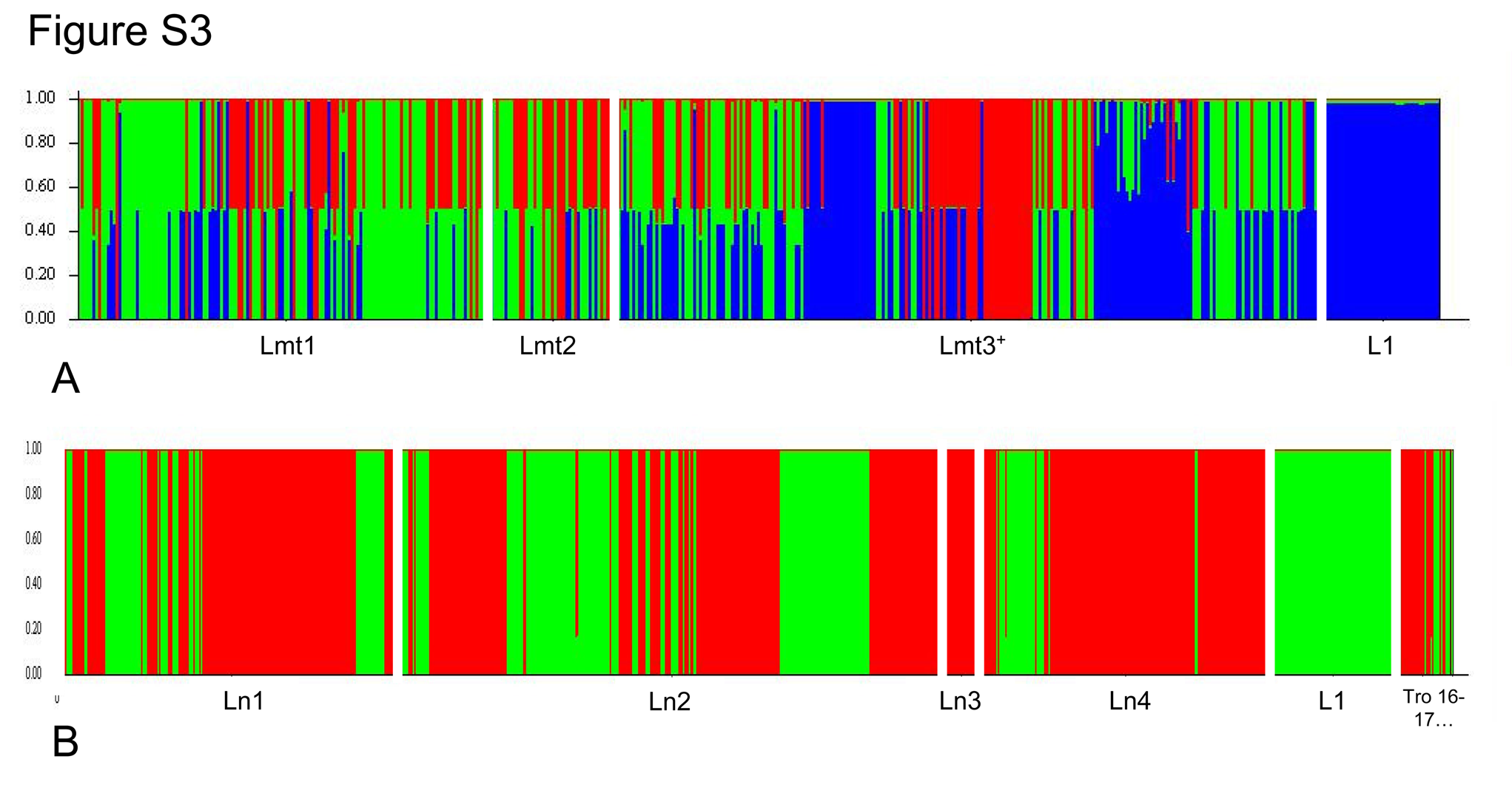

Supplement: Figure S3 — Structure Q plots results sorted out by mitochondrial/nuclear lineage membership. A. Tpm Q plots sorted out by the COI lineage membership individual information B. COI Q plots sorted out by the Tpm lineage membership individual information. (TIF) [file pone.0022305.s003.tif]

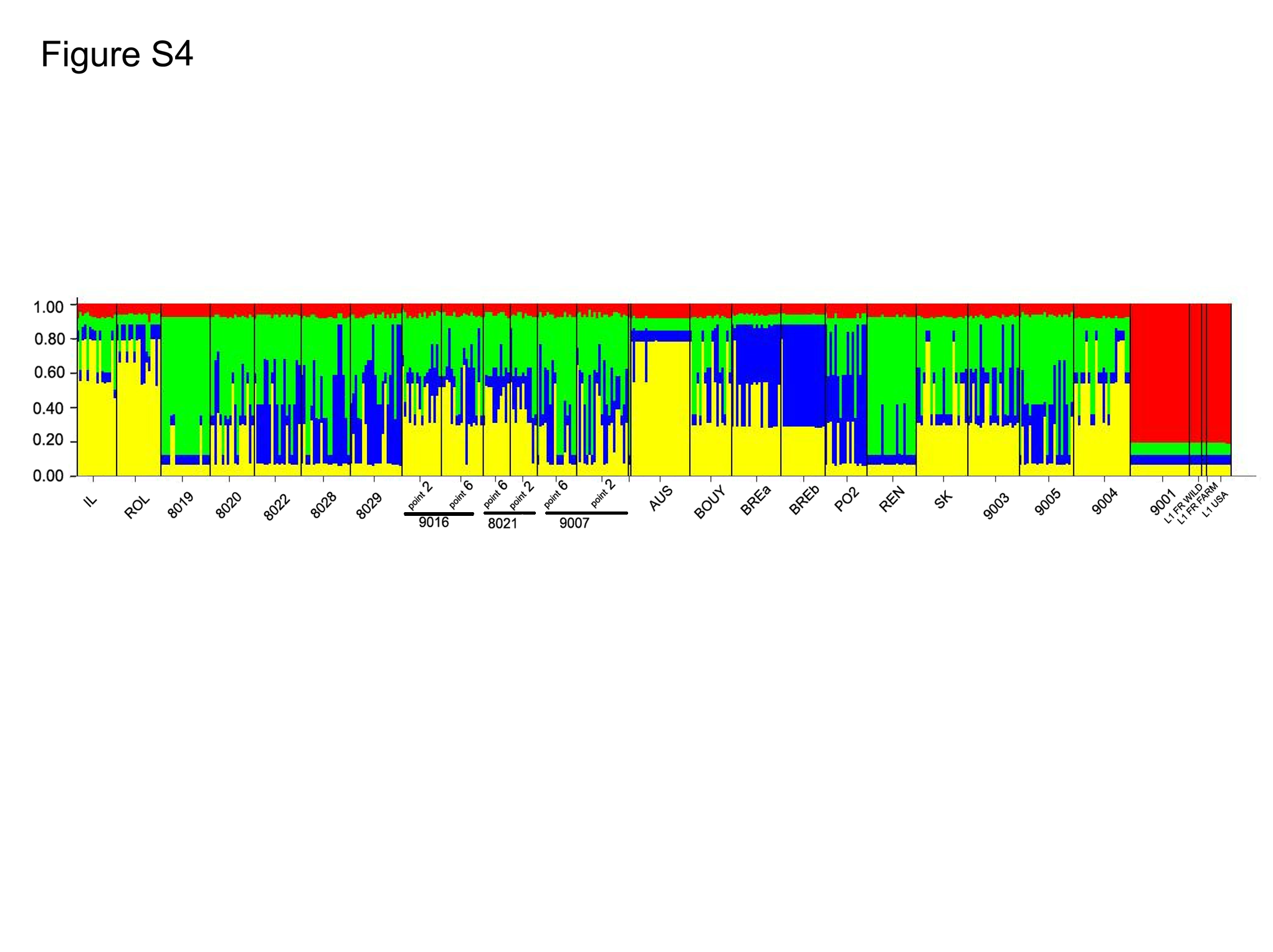

Supplement: Figure S4 — Structure Q plots representing COI and Tpm first-level analyses as inferred using the linkage model. Combined COI and Tpm whole sequence datasets, K = 4. Linkage has been evaluated based on mapped distances between sites. (TIF) [file pone.0022305.s004.tif]

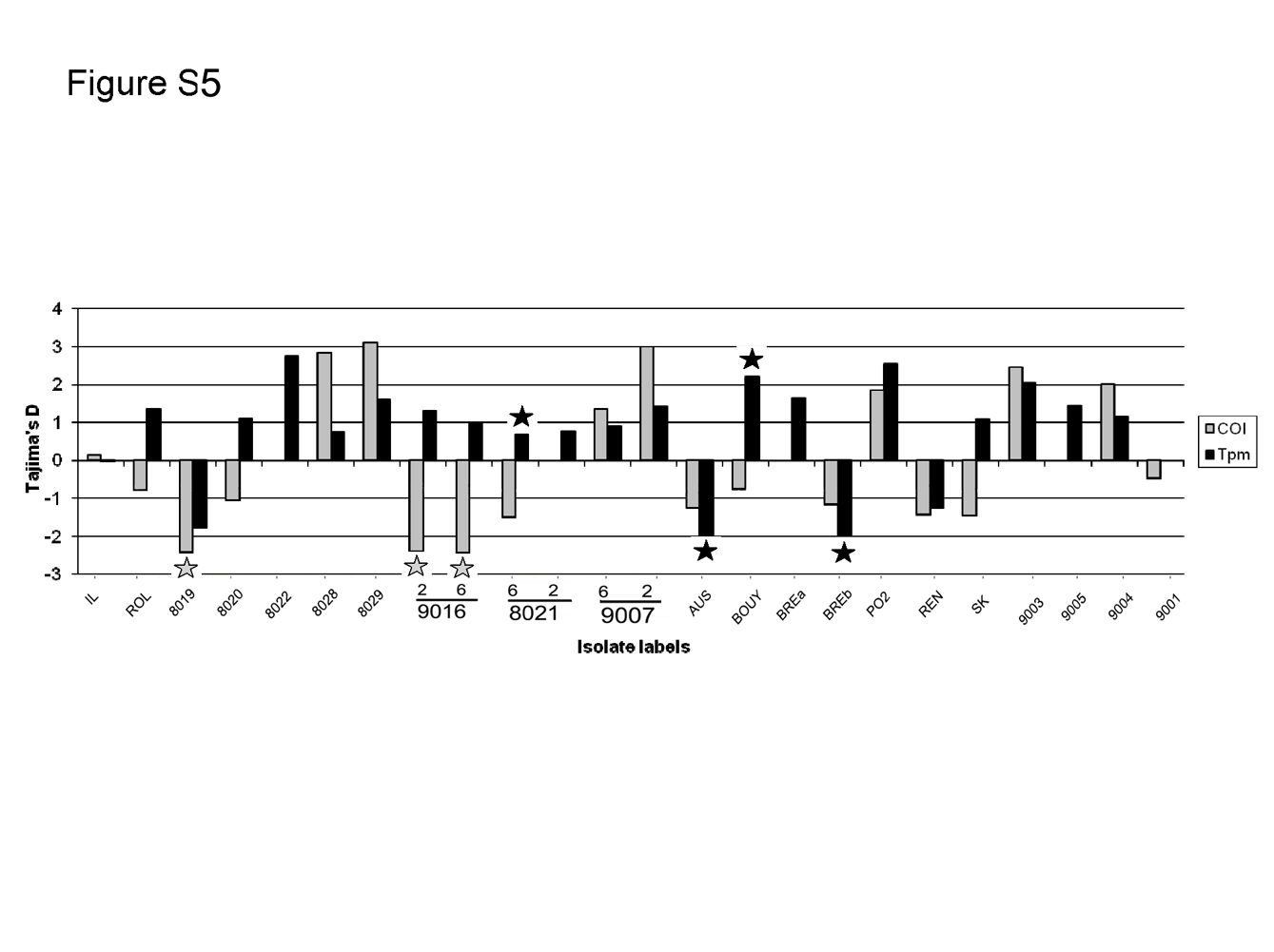

Supplement: Figure S5 — Histogram representing obtained Tajima D values (Arlequin). Black and grey stars indicate significant D values (P<0.05). (TIF) [file pone.0022305.s005.tif]

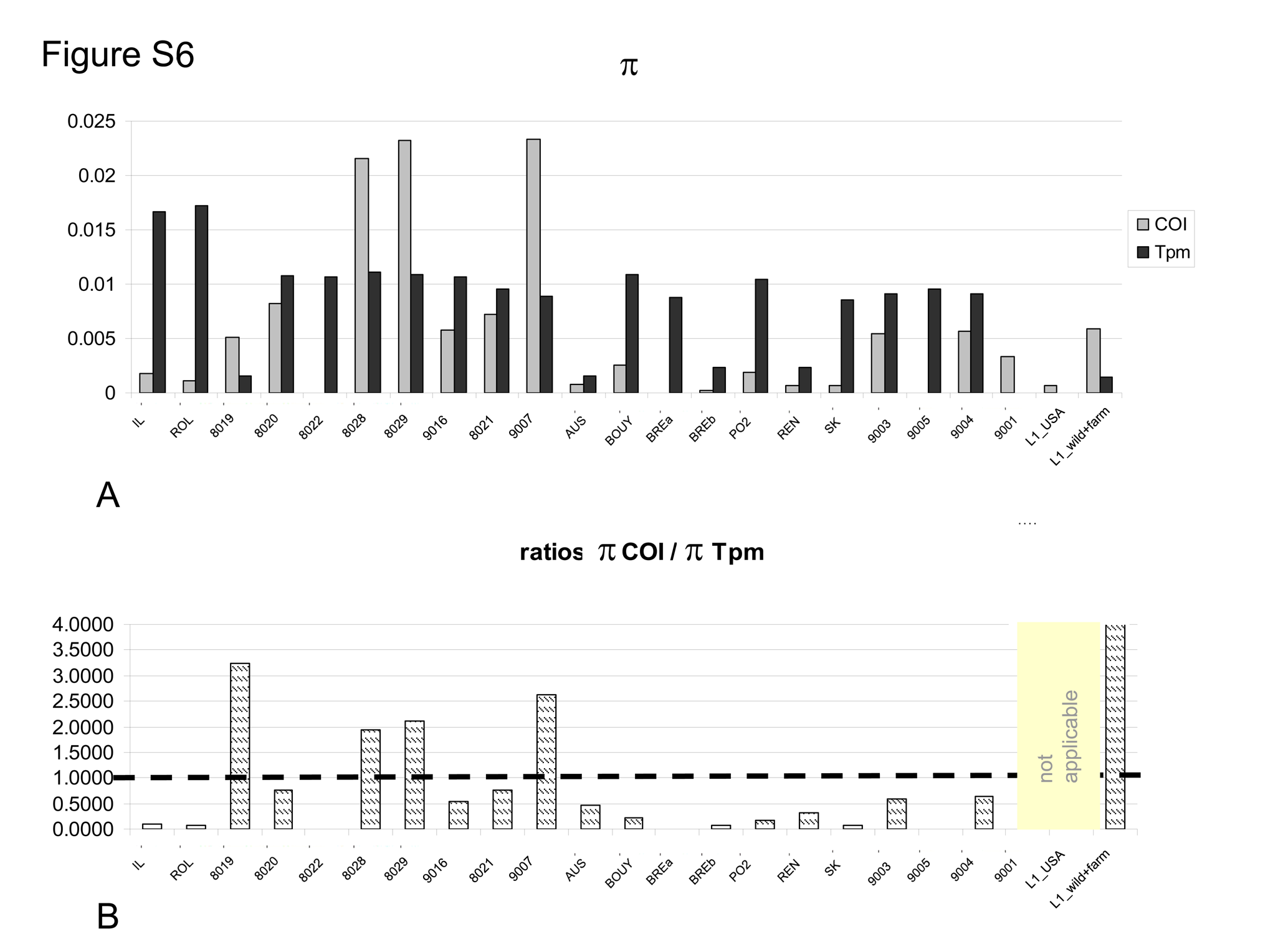

Supplement: Figure S6 — Nucleotidic diversities. Values of π in each isolate as calculated using DnaSP following Nei (1987; equation 10.5). A. Absolute values. B. Ratio π COI/π Tpm. (TIF) [file pone.0022305.s006.tif]
