# Supplementary material for: Using Mitochondrial and Nuclear Sequence Data for Disentangling Population Structure in Complex Pest Species: A Case Study with Dermanyssus gallinae
Source: PLoS One. 2011 Jul 25;6(7):e22305. doi: 10.1371/journal.pone.0022305 (PMC3143145; doi:10.1371/journal.pone.0022305)
Supplement: Table S1 — Information on mite isolates including Genbank accession numbers. (DOC) [file pone.0022305.s007.doc]

Table S1-A. Per isolate information.

| Isolate label (some are common to Roy [21] and Roy *et al*. [19,20,22,23,27]) | Number of genotyped individuals at both COI and Tpm | Accession numbers COI | Accession numbers Tpm | Sampling year | Sampling place | Context | Remarks |
| --- | --- | --- | --- | --- | --- | --- | --- |
| IL | 16 | HQ842440- HQ842455 | HQ841727- HQ841758 | 2007 | Groningen, The Netherlands | Nests of Common Starlings |  |
| ROL | 18 | HQ842515- HQ842532 | HQ841919- HQ841954 | 2008 | Bouches du Rhône, France | Tree holes occupied by European Rollers |  |
| 8019 | 20 | HQ842218- HQ842237 | HQ841189- HQ841228 | 2008 | Ardèche, France | Layer farm, organic |  |
| 8020 | 18 | HQ842238- HQ842255 | HQ841277- HQ841312 | 2008 | Maine-et-Loire, France | Layer farm, conventional |  |
| 8022 | 19 | HQ842256- HQ842274 | HQ841313- HQ841350 | 2008 | Maine-et-Loire, France | Layer farm, conventional |  |
| 8028 | 20 | HQ842275- HQ842294 | HQ841351- HQ841390 | 2008 | Ain, France | Layer farm | Bresse region. |
| 8029 | 21 | HQ842295- HQ842315 | HQ841793- HQ841834 | 2008 | Ain, France | Layer farm | Bresse region. |
| 9016  (points 2, 6) | 16, 17 | HQ842162- HQ842194 | HQ841545- HQ841610 | 2009 | Ain, France | Layer farm | Bresse region.  Isolates 9016, 8021 and 9007 have been collected in the focused farm CON, within which 2 different building (1 and 5) and 2 different points within building were sampled (see Appendix 2). Building 1 has been sampled twice, in 2008 (8021), then in 2009 (9016), on two different successive flocks. |
| 8021  (points 6, 2) | 11, 11 | HQ842195- HQ842216 | HQ841501- HQ841544 | 2008 | Ain, France | Layer farm |
| 9007  (points 6, 2) | 16, 21 | HQ842125- HQ842161 | HQ841611- HQ841684 | 2009 | Ain, France | Layer farm |
| AUS | 24 | HQ842340- HQ842363 | HQ841229- HQ841276 | 2009 | Victoria, Australia | Layer farm, conventional |  |
| BOUY | 17 | HQ842364- HQ842380 | HQ841391- HQ841424 | 2009 | Drôme, France | Amateur layers |  |
| BREa | 20 | HQ842381- HQ842400 | HQ841425- HQ841464 | 2009 | Bastos-SP, Brazil | Layer farm |  |
| BREb | 18 | HQ842401- HQ842418 | HQ841465- HQ841500 | 2009 | Salvador do Sul, Brazil | Layer farm |  |
| PO2 | 17 | HQ842456- HQ842472 | HQ841759- HQ841792 | 2007 | Poland | Layer farm |  |
| REN | 20 | HQ842473- HQ842492 | HQ841835- HQ841874 | 2009 | Ille-et-Vilaine, France | Layer farm, conventional |  |
| SK | 21 | HQ842533- HQ842553 | HQ842003- HQ842044 | 2008 | Denmark | Layer farm | Reared at lab since 1997 |
| 9003 | 21 | HQ842419- HQ842439 | HQ841685- HQ841726 | 2009 | Ain, France | Broiler “Poulet de Bresse AOC” | AOC = controlled designation of origin. All these isolates are located in the Bresse region. |
| 9005 | 22 | HQ842493- HQ842514 | HQ841875- HQ841918 | 2009 | Sâone-et-Loire, France | Broiler “Poulet de Bresse AOC” |
| 9004 | 23 | HQ842571- HQ842593 | HQ842045- HQ842090 | 2009 | Sâone-et-Loire, France | Broiler “Poulet de Bresse AOC” |
| 9001 | 24 | HQ842316- HQ842339 | HQ841955- HQ842002 | 2009 | Rhône, France | Pigeon breeding facility |  |
| L1FR | 5 | HQ842564- HQ842570 | HQ842091- HQ842104 | 2007-2008 | Drôme, Rhône, Bouches du Rhône, FR | Nests and environment of pigeons, pigeon breeding facilities |  |
| L1US | 10 | HQ842554- HQ842563 | HQ842105- HQ842124 | 2009 | Utah, USA | Nests and environment of pigeons |  |
| BAU | 16 |  | - | 2010 | Deux-Sèvres | Layer farm | Only COI was sequenced in this isolate. |

Table S1-B. Per individual information

| Isolate |  | Individual (= COI seq label) | Individual (= Tpm seq label) | Acc. no. Tpm | Acc.no.COI |
| --- | --- | --- | --- | --- | --- |
| 8006 |  | 8006j | 8006_111c2 |  | HQ842217 |
| 8006 |  | 8006j | 8006_112c2 |  | - |
| 8019 |  | 8019dprim | 8019_113c2 | HQ841189 | HQ842218 |
| 8019 |  | 8019dprim | 8019_114c2 | HQ841190 | - |
| 8019 |  | 8019eprim | 8019_115c2 | HQ841191 | HQ842219 |
| 8019 |  | 8019eprim | 8019_116c2 | HQ841192 | - |
| 8019 |  | 8019f | 8019_117c2 | HQ841193 | HQ842220 |
| 8019 |  | 8019f | 8019_118c2 | HQ841194 | - |
| 8019 |  | 8019g | 8019_119c2 | HQ841195 | HQ842221 |
| 8019 |  | 8019g | 8019_120c2 | HQ841196 | - |
| 8019 |  | 8019h | 8019_121c2 | HQ841197 | HQ842222 |
| 8019 |  | 8019h | 8019_122c2 | HQ841198 | - |
| 8019 |  | 8019j | 8019_123c3 | HQ841199 | HQ842223 |
| 8019 |  | 8019j | 8019_124c3 | HQ841200 | - |
| 8019 |  | 8019k | 8019_125c2 | HQ841201 | HQ842224 |
| 8019 |  | 8019k | 8019_126c2 | HQ841202 | - |
| 8019 |  | 8019l | 8019_127c2 | HQ841203 | HQ842225 |
| 8019 |  | 8019l | 8019_128c2 | HQ841204 | - |
| 8019 |  | 8019m | 8019_129c2 | HQ841205 | HQ842226 |
| 8019 |  | 8019m | 8019_130c2 | HQ841206 | - |
| 8019 |  | 8019n | 8019_131c2 | HQ841207 | HQ842227 |
| 8019 |  | 8019n | 8019_132c2 | HQ841208 | - |
| 8019 |  | 8019o | 8019_133c2 | HQ841209 | HQ842228 |
| 8019 |  | 8019o | 8019_134c2 | HQ841210 | - |
| 8019 |  | 8019p | 8019_135c2 | HQ841211 | HQ842229 |
| 8019 |  | 8019p | 8019_136c2 | HQ841212 | - |
| 8019 |  | 8019q | 8019_137c2 | HQ841213 | HQ842230 |
| 8019 |  | 8019q | 8019_138c2 | HQ841214 | - |
| 8019 |  | 8019s | 8019_139c2 | HQ841215 | HQ842231 |
| 8019 |  | 8019s | 8019_140c2 | HQ841216 | - |
| 8019 |  | 8019t | 8019_141c2 | HQ841217 | HQ842232 |
| 8019 |  | 8019t | 8019_142c2 | HQ841218 | - |
| 8019 |  | 8019u | 8019_143c2 | HQ841219 | HQ842233 |
| 8019 |  | 8019u | 8019_144c2 | HQ841220 | - |
| 8019 |  | 8019v | 8019_145c2 | HQ841221 | HQ842234 |
| 8019 |  | 8019v | 8019_146c2 | HQ841222 | - |
| 8019 |  | 8019w | 8019_147c2 | HQ841223 | HQ842235 |
| 8019 |  | 8019w | 8019_148c2 | HQ841224 | - |
| 8019 |  | 8019x | 8019_149c2 | HQ841225 | HQ842236 |
| 8019 |  | 8019x | 8019_150c2 | HQ841226 | - |
| 8019 |  | 8019y | 8019_151c2 | HQ841227 | HQ842237 |
| 8019 |  | 8019y | 8019_152c2 | HQ841228 | - |
| 8020 |  | 8020b | 8020_153c2 | HQ841277 | HQ842238 |
| 8020 |  | 8020b | 8020_154c2 | HQ841278 | - |
| 8020 |  | 8020c | 8020_155c2 | HQ841279 | HQ842239 |
| 8020 |  | 8020c | 8020_156c2 | HQ841280 | - |
| 8020 |  | 8020e | 8020_157c2 | HQ841281 | HQ842240 |
| 8020 |  | 8020e | 8020_158c2 | HQ841282 | - |
| 8020 |  | 8020f | 8020_159c | HQ841283 | HQ842241 |
| 8020 |  | 8020f | 8020_160c | HQ841284 | - |
| 8020 |  | 8020g | 8020_161c2 | HQ841285 | HQ842242 |
| 8020 |  | 8020g | 8020_162c2 | HQ841286 | - |
| 8020 |  | 8020h | 8020_163c2 | HQ841287 | HQ842243 |
| 8020 |  | 8020h | 8020_164c2 | HQ841288 | - |
| 8020 |  | 8020i | 8020_165c2 | HQ841289 | HQ842244 |
| 8020 |  | 8020i | 8020_166c2 | HQ841290 | - |
| 8020 |  | 8020j | 8020_167c6 | HQ841291 | HQ842245 |
| 8020 |  | 8020j | 8020_168c6 | HQ841292 | - |
| 8020 |  | 8020k | 8020_169c2 | HQ841293 | HQ842246 |
| 8020 |  | 8020k | 8020_170c2 | HQ841294 | - |
| 8020 |  | 8020l | 8020_171c2 | HQ841295 | HQ842247 |
| 8020 |  | 8020l | 8020_172c2 | HQ841296 | - |
| 8020 |  | 8020m | 8020_173c2 | HQ841297 | HQ842248 |
| 8020 |  | 8020m | 8020_174c2 | HQ841298 | - |
| 8020 |  | 8020n | 8020_175c2 | HQ841299 | HQ842249 |
| 8020 |  | 8020n | 8020_176c2 | HQ841300 | - |
| 8020 |  | 8020o | 8020_177c2 | HQ841301 | HQ842250 |
| 8020 |  | 8020o | 8020_178c2 | HQ841302 | - |
| 8020 |  | 8020p | 8020_179c2 | HQ841303 | HQ842251 |
| 8020 |  | 8020p | 8020_180c2 | HQ841304 | - |
| 8020 |  | 8020q | 8020_181c2 | HQ841305 | HQ842252 |
| 8020 |  | 8020q | 8020_182c2 | HQ841306 | - |
| 8020 |  | 8020s | 8020_183c2 | HQ841307 | HQ842253 |
| 8020 |  | 8020s | 8020_184c2 | HQ841308 | - |
| 8020 |  | 8020t | 8020_185c2 | HQ841309 | HQ842254 |
| 8020 |  | 8020t | 8020_186c2 | HQ841310 | - |
| 8020 |  | 8020u | 8020_187c2 | HQ841311 | HQ842255 |
| 8020 |  | 8020u | 8020_188c2 | HQ841312 | - |
| 8021 | sampling point n°2 | 8021_M2g | 8021_100c3 | HQ841534 | HQ842195 |
| 8021 | sampling point n°2 | 8021_M2h | 8021_101c3 | HQ841535 | - |
| 8021 | sampling point n°2 | 8021_M2h | 8021_102c9 | HQ841536 | HQ842196 |
| 8021 | sampling point n°2 | 8021_M2i | 8021_103c3 | HQ841537 | - |
| 8021 | sampling point n°2 | 8021_M2i | 8021_104c7 | HQ841538 | HQ842197 |
| 8021 | sampling point n°2 | 8021_M2j | 8021_105c3 | HQ841539 | - |
| 8021 | sampling point n°2 | 8021_M2j | 8021_106c7 | HQ841540 | HQ842198 |
| 8021 | sampling point n°2 | 8021_M2k | 8021_107c6 | HQ841541 | - |
| 8021 | sampling point n°2 | 8021_M2k | 8021_108c6 | HQ841542 | HQ842199 |
| 8021 | sampling point n°2 | 8021_M2l | 8021_109c3 | HQ841543 | - |
| 8021 | sampling point n°2 | 8021_M2l | 8021_110c3 | HQ841544 | HQ842200 |
| 8021 | sampling point n°6 | 8021_6a | 8021_67c3 | HQ841501 | - |
| 8021 | sampling point n°6 | 8021_6a | 8021_68c3 | HQ841502 | HQ842201 |
| 8021 | sampling point n°6 | 8021_6b | 8021_69c3 | HQ841503 | - |
| 8021 | sampling point n°6 | 8021_6b | 8021_70c9 | HQ841504 | HQ842202 |
| 8021 | sampling point n°6 | 8021_6c | 8021_71c3 | HQ841505 | - |
| 8021 | sampling point n°6 | 8021_6c | 8021_72c9 | HQ841506 | HQ842203 |
| 8021 | sampling point n°6 | 8021_6d | 8021_73c3 | HQ841507 | - |
| 8021 | sampling point n°6 | 8021_6d | 8021_74c9 | HQ841508 | HQ842204 |
| 8021 | sampling point n°6 | 8021_6e | 8021_75c3 | HQ841509 | - |
| 8021 | sampling point n°6 | 8021_6e | 8021_76c7 | HQ841510 | HQ842205 |
| 8021 | sampling point n°6 | 8021_6f | 8021_77c3 | HQ841511 | - |
| 8021 | sampling point n°6 | 8021_6f | 8021_78c7 | HQ841512 | HQ842206 |
| 8021 | sampling point n°6 | 8021_6g | 8021_79c3 | HQ841513 | - |
| 8021 | sampling point n°6 | 8021_6g | 8021_80c9 | HQ841514 | HQ842207 |
| 8021 | sampling point n°6 | 8021_6h | 8021_81c7 | HQ841515 | - |
| 8021 | sampling point n°6 | 8021_6h | 8021_82c9 | HQ841516 | HQ842208 |
| 8021 | sampling point n°6 | 8021_6j | 8021_83c3 | HQ841517 | - |
| 8021 | sampling point n°6 | 8021_6j | 8021_84c9 | HQ841518 | HQ842209 |
| 8021 | sampling point n°6 | 8021_6k | 8021_85c3 | HQ841519 | - |
| 8021 | sampling point n°6 | 8021_6k | 8021_86c7 | HQ841520 | HQ842210 |
| 8021 | sampling point n°6 | 8021_6l | 8021_87c5 | HQ841521 | - |
| 8021 | sampling point n°6 | 8021_6l | 8021_88c3 | HQ841522 | HQ842211 |
| 8021 | sampling point n°2 | 8021_M2a | 8021_89c5 | HQ841523 | - |
| 8021 | sampling point n°2 | 8021_M2a | 8021_90c7 | HQ841524 | HQ842212 |
| 8021 | sampling point n°2 | 8021_M2c | 8021_91c2 | HQ841525 | - |
| 8021 | sampling point n°2 | 8021_M2c | 8021_92c | HQ841526 | HQ842213 |
| 8021 | sampling point n°2 | 8021_M2d | 8021_93c3 | HQ841527 | - |
| 8021 | sampling point n°2 | 8021_M2d | 8021_94c7 | HQ841528 | HQ842214 |
| 8021 | sampling point n°2 | 8021_M2e | 8021_95c3 | HQ841529 | - |
| 8021 | sampling point n°2 | 8021_M2e | 8021_96c9 | HQ841530 | HQ842215 |
| 8021 | sampling point n°2 | 8021_M2f | 8021_97c3 | HQ841531 | - |
| 8021 | sampling point n°2 | 8021_M2f | 8021_98c9 | HQ841532 | HQ842216 |
| 8021 | sampling point n°2 | 8021_M2g | 8021_99c5 | HQ841533 | - |
| 8022 |  | 8022a | 8022_189c2 | HQ841313 | HQ842256 |
| 8022 |  | 8022a | 8022_190c2 | HQ841314 | - |
| 8022 |  | 8022c | 8022_191c2 | HQ841315 | HQ842257 |
| 8022 |  | 8022c | 8022_192c2 | HQ841316 | - |
| 8022 |  | 8022e | 8022_193c2 | HQ841317 | HQ842258 |
| 8022 |  | 8022e | 8022_194c2 | HQ841318 | - |
| 8022 |  | 8022f | 8022_195c2 | HQ841319 | HQ842259 |
| 8022 |  | 8022f | 8022_196c2 | HQ841320 | - |
| 8022 |  | 8022g | 8022_197c2 | HQ841321 | HQ842260 |
| 8022 |  | 8022g | 8022_198c2 | HQ841322 | - |
| 8022 |  | 8022h | 8022_199c2 | HQ841323 | HQ842261 |
| 8022 |  | 8022h | 8022_200c2 | HQ841324 | - |
| 8022 |  | 8022i | 8022_201c2 | HQ841325 | HQ842262 |
| 8022 |  | 8022i | 8022_202c2 | HQ841326 | - |
| 8022 |  | 8022j | 8022_203c2 | HQ841327 | HQ842263 |
| 8022 |  | 8022j | 8022_204c2 | HQ841328 | - |
| 8022 |  | 8022k | 8022_205c2 | HQ841329 | HQ842264 |
| 8022 |  | 8022k | 8022_206c2 | HQ841330 | - |
| 8022 |  | 8022l | 8022_207c2 | HQ841331 | HQ842265 |
| 8022 |  | 8022l | 8022_208c2 | HQ841332 | - |
| 8022 |  | 8022m | 8022_209c2 | HQ841333 | HQ842266 |
| 8022 |  | 8022m | 8022_210c2 | HQ841334 | - |
| 8022 |  | 8022n | 8022_211c2 | HQ841335 | HQ842267 |
| 8022 |  | 8022n | 8022_212c2 | HQ841336 | - |
| 8022 |  | 8022o | 8022_213c2 | HQ841337 | HQ842268 |
| 8022 |  | 8022o | 8022_214c2 | HQ841338 | - |
| 8022 |  | 8022p | 8022_215c2 | HQ841339 | HQ842269 |
| 8022 |  | 8022p | 8022_216c2 | HQ841340 | - |
| 8022 |  | 8022q | 8022_217c2 | HQ841341 | HQ842270 |
| 8022 |  | 8022q | 8022_218c2 | HQ841342 | - |
| 8022 |  | 8022t | 8022_219c2 | HQ841343 | HQ842271 |
| 8022 |  | 8022t | 8022_220c2 | HQ841344 | - |
| 8022 |  | 8022w | 8022_221c2 | HQ841345 | HQ842272 |
| 8022 |  | 8022w | 8022_222c2 | HQ841346 | - |
| 8022 |  | 8022x | 8022_223c2 | HQ841347 | HQ842273 |
| 8022 |  | 8022x | 8022_224c2 | HQ841348 | - |
| 8022 |  | 8022y | 8022_225c2 | HQ841349 | HQ842274 |
| 8022 |  | 8022y | 8022_226c2 | HQ841350 | - |
| 8028 |  | 8028d | 8028_227c6 | HQ841351 | HQ842275 |
| 8028 |  | 8028d | 8028_228c6 | HQ841352 | - |
| 8028 |  | 8028f | 8028_229c2 | HQ841353 | HQ842276 |
| 8028 |  | 8028f | 8028_230c2 | HQ841354 | - |
| 8028 |  | 8028g | 8028_231c2 | HQ841355 | HQ842277 |
| 8028 |  | 8028g | 8028_232c2 | HQ841356 | - |
| 8028 |  | 8028h | 8028_233c2 | HQ841357 | HQ842278 |
| 8028 |  | 8028h | 8028_234c2 | HQ841358 | - |
| 8028 |  | 8028i | 8028_235c | HQ841359 | HQ842279 |
| 8028 |  | 8028i | 8028_236c2 | HQ841360 | - |
| 8028 |  | 8028j | 8028_237c2 | HQ841361 | HQ842280 |
| 8028 |  | 8028j | 8028_238c2 | HQ841362 | - |
| 8028 |  | 8028k | 8028_239c6 | HQ841363 | HQ842281 |
| 8028 |  | 8028k | 8028_240c6 | HQ841364 | - |
| 8028 |  | 8028l | 8028_241c6 | HQ841365 | HQ842282 |
| 8028 |  | 8028l | 8028_242c6 | HQ841366 | - |
| 8028 |  | 8028m | 8028_243c | HQ841367 | HQ842283 |
| 8028 |  | 8028m | 8028_244c6 | HQ841368 | - |
| 8028 |  | 8028n | 8028_245c6 | HQ841369 | HQ842284 |
| 8028 |  | 8028n | 8028_246c6 | HQ841370 | - |
| 8028 |  | 8028o | 8028_247c6 | HQ841371 | HQ842285 |
| 8028 |  | 8028o | 8028_248c6 | HQ841372 | - |
| 8028 |  | 8028p | 8028_249c2 | HQ841373 | HQ842286 |
| 8028 |  | 8028p | 8028_250c2 | HQ841374 | - |
| 8028 |  | 8028q | 8028_251c2 | HQ841375 | HQ842287 |
| 8028 |  | 8028q | 8028_252c2 | HQ841376 | - |
| 8028 |  | 8028r | 8028_253c6 | HQ841377 | HQ842288 |
| 8028 |  | 8028r | 8028_254c6 | HQ841378 | - |
| 8028 |  | 8028s | 8028_255c6 | HQ841379 | HQ842289 |
| 8028 |  | 8028s | 8028_256c6 | HQ841380 | - |
| 8028 |  | 8028t | 8028_257c6 | HQ841381 | HQ842290 |
| 8028 |  | 8028t | 8028_258c6 | HQ841382 | - |
| 8028 |  | 8028u | 8028_259c6 | HQ841383 | HQ842291 |
| 8028 |  | 8028u | 8028_260c6 | HQ841384 | - |
| 8028 |  | 8028v | 8028_261c6 | HQ841385 | HQ842292 |
| 8028 |  | 8028v | 8028_262c6 | HQ841386 | - |
| 8028 |  | 8028w | 8028_263c6 | HQ841387 | HQ842293 |
| 8028 |  | 8028w | 8028_264c6 | HQ841388 | - |
| 8028 |  | 8028x | 8028_265c2 | HQ841389 | HQ842294 |
| 8028 |  | 8028x | 8028_266c2 | HQ841390 | - |
| 8029 |  | 8029a | 8029_267c2 | HQ841793 | HQ842295 |
| 8029 |  | 8029a | 8029_268c2 | HQ841794 | - |
| 8029 |  | 8029b | 8029_269c | HQ841795 | HQ842296 |
| 8029 |  | 8029b | 8029_270c6 | HQ841796 | - |
| 8029 |  | 8029c | 8029_271c6 | HQ841797 | HQ842297 |
| 8029 |  | 8029c | 8029_272c6 | HQ841798 | - |
| 8029 |  | 8029d | 8029_273c6 | HQ841799 | HQ842298 |
| 8029 |  | 8029d | 8029_274c6 | HQ841800 | - |
| 8029 |  | 8029e | 8029_275c6 | HQ841801 | HQ842299 |
| 8029 |  | 8029e | 8029_276c6 | HQ841802 | - |
| 8029 |  | 8029f | 8029_277c2 | HQ841803 | HQ842300 |
| 8029 |  | 8029f | 8029_278c2 | HQ841804 | - |
| 8029 |  | 8029g | 8029_279c2 | HQ841805 | HQ842301 |
| 8029 |  | 8029g | 8029_280c2 | HQ841806 | - |
| 8029 |  | 8029h | 8029_281c2 | HQ841807 | HQ842302 |
| 8029 |  | 8029h | 8029_282c2 | HQ841808 | - |
| 8029 |  | 8029i | 8029_283c2 | HQ841809 | HQ842303 |
| 8029 |  | 8029i | 8029_284c2 | HQ841810 | - |
| 8029 |  | 8029j | 8029_285c6 | HQ841811 | HQ842304 |
| 8029 |  | 8029j | 8029_286c6 | HQ841812 | - |
| 8029 |  | 8029k | 8029_287c6 | HQ841813 | HQ842305 |
| 8029 |  | 8029k | 8029_288c6 | HQ841814 | - |
| 8029 |  | 8029l | 8029_289c2 | HQ841815 | HQ842306 |
| 8029 |  | 8029l | 8029_290c2 | HQ841816 | - |
| 8029 |  | 8029m | 8029_291c2 | HQ841817 | HQ842307 |
| 8029 |  | 8029m | 8029_292c2 | HQ841818 | - |
| 8029 |  | 8029n | 8029_293c2 | HQ841819 | HQ842308 |
| 8029 |  | 8029n | 8029_294c2 | HQ841820 | - |
| 8029 |  | 8029o | 8029_295c2 | HQ841821 | HQ842309 |
| 8029 |  | 8029o | 8029_296c2 | HQ841822 | - |
| 8029 |  | 8029p | 8029_297c2 | HQ841823 | HQ842310 |
| 8029 |  | 8029p | 8029_298c2 | HQ841824 | - |
| 8029 |  | 8029q | 8029_299c6 | HQ841825 | HQ842311 |
| 8029 |  | 8029q | 8029_300c6 | HQ841826 | - |
| 8029 |  | 8029s | 8029_301c6 | HQ841827 | HQ842312 |
| 8029 |  | 8029s | 8029_302c6 | HQ841828 | - |
| 8029 |  | 8029t | 8029_303c6 | HQ841829 | HQ842313 |
| 8029 |  | 8029t | 8029_304c6 | HQ841830 | - |
| 8029 |  | 8029u | 8029_305c6 | HQ841831 | HQ842314 |
| 8029 |  | 8029u | 8029_306c6 | HQ841832 | - |
| 8029 |  | 8029v | 8029_307c6 | HQ841833 | HQ842315 |
| 8029 |  | 8029v | 8029_308c6 | HQ841834 | - |
| 9001 |  | 9001a | 9001_309c4 | HQ841955 | HQ842316 |
| 9001 |  | 9001a | 9001_310c4 | HQ841956 | - |
| 9001 |  | 9001b | 9001_311c4 | HQ841957 | HQ842317 |
| 9001 |  | 9001b | 9001_312c4 | HQ841958 | - |
| 9001 |  | 9001c | 9001_313c4 | HQ841959 | HQ842318 |
| 9001 |  | 9001c | 9001_314c4 | HQ841960 | - |
| 9001 |  | 9001d | 9001_315c4 | HQ841961 | HQ842319 |
| 9001 |  | 9001d | 9001_316c4 | HQ841962 | - |
| 9001 |  | 9001e | 9001_317c4 | HQ841963 | HQ842320 |
| 9001 |  | 9001e | 9001_318c4 | HQ841964 | - |
| 9001 |  | 9001f | 9001_319c4 | HQ841965 | HQ842321 |
| 9001 |  | 9001f | 9001_320c4 | HQ841966 | - |
| 9001 |  | 9001g | 9001_321c4 | HQ841967 | HQ842322 |
| 9001 |  | 9001g | 9001_322c4 | HQ841968 | - |
| 9001 |  | 9001h | 9001_323c4 | HQ841969 | HQ842323 |
| 9001 |  | 9001h | 9001_324c4 | HQ841970 | - |
| 9001 |  | 9001i | 9001_325c4 | HQ841971 | HQ842324 |
| 9001 |  | 9001i | 9001_326c4 | HQ841972 | - |
| 9001 |  | 9001j | 9001_327c4 | HQ841973 | HQ842325 |
| 9001 |  | 9001j | 9001_328c4 | HQ841974 | - |
| 9001 |  | 9001k | 9001_329c4 | HQ841975 | HQ842326 |
| 9001 |  | 9001k | 9001_330c4 | HQ841976 | - |
| 9001 |  | 9001l | 9001_331c4 | HQ841977 | HQ842327 |
| 9001 |  | 9001l | 9001_332c4 | HQ841978 | - |
| 9001 |  | 9001m | 9001_333c4 | HQ841979 | HQ842328 |
| 9001 |  | 9001m | 9001_334c4 | HQ841980 | - |
| 9001 |  | 9001n | 9001_335c4 | HQ841981 | HQ842329 |
| 9001 |  | 9001n | 9001_336c4 | HQ841982 | - |
| 9001 |  | 9001o | 9001_337c4 | HQ841983 | HQ842330 |
| 9001 |  | 9001o | 9001_338c4 | HQ841984 | - |
| 9001 |  | 9001p | 9001_339c4 | HQ841985 | HQ842331 |
| 9001 |  | 9001p | 9001_340c4 | HQ841986 | - |
| 9001 |  | 9001q | 9001_341c4 | HQ841987 | HQ842332 |
| 9001 |  | 9001q | 9001_342c4 | HQ841988 | - |
| 9001 |  | 9001r | 9001_343c4 | HQ841989 | HQ842333 |
| 9001 |  | 9001r | 9001_344c4 | HQ841990 | - |
| 9001 |  | 9001s | 9001_345c4 | HQ841991 | HQ842334 |
| 9001 |  | 9001s | 9001_346c4 | HQ841992 | - |
| 9001 |  | 9001t | 9001_347c4 | HQ841993 | HQ842335 |
| 9001 |  | 9001t | 9001_348c4 | HQ841994 | - |
| 9001 |  | 9001u | 9001_349c4 | HQ841995 | HQ842336 |
| 9001 |  | 9001u | 9001_350c4 | HQ841996 | - |
| 9001 |  | 9001v | 9001_351c4 | HQ841997 | HQ842337 |
| 9001 |  | 9001v | 9001_352c4 | HQ841998 | - |
| 9001 |  | 9001w | 9001_353c4 | HQ841999 | HQ842338 |
| 9001 |  | 9001w | 9001_354c4 | HQ842000 | - |
| 9001 |  | 9001x | 9001_355c4 | HQ842001 | HQ842339 |
| 9001 |  | 9001x | 9001_356c4 | HQ842002 | - |
| 9003 |  | 9003_1 | 9003_675c3 | HQ841685 | HQ842419 |
| 9003 |  | 9003_1 | 9003_696c3 | HQ841706 | - |
| 9003 |  | 9003_10 | 9003_676c8 | HQ841686 | HQ842420 |
| 9003 |  | 9003_10 | 9003_677c7 | HQ841687 | - |
| 9003 |  | 9003_11 | 9003_678c5 | HQ841688 | HQ842421 |
| 9003 |  | 9003_11 | 9003_679c3 | HQ841689 | - |
| 9003 |  | 9003_12 | 9003_680c8 | HQ841690 | HQ842422 |
| 9003 |  | 9003_12 | 9003_681c7 | HQ841691 | - |
| 9003 |  | 9003_13 | 9003_682c3 | HQ841692 | HQ842423 |
| 9003 |  | 9003_13 | 9003_683c3 | HQ841693 | - |
| 9003 |  | 9003_14 | 9003_684c7 | HQ841694 | HQ842424 |
| 9003 |  | 9003_14 | 9003_685c7 | HQ841695 | - |
| 9003 |  | 9003_15 | 9003_686c8 | HQ841696 | HQ842425 |
| 9003 |  | 9003_15 | 9003_687c7 | HQ841697 | - |
| 9003 |  | 9003_16 | 9003_688c | HQ841698 | HQ842426 |
| 9003 |  | 9003_16 | 9003_689c8 | HQ841699 | - |
| 9003 |  | 9003_17 | 9003_690c | HQ841700 | HQ842427 |
| 9003 |  | 9003_17 | 9003_691c8 | HQ841701 | - |
| 9003 |  | 9003_18 | 9003_692c3 | HQ841702 | HQ842428 |
| 9003 |  | 9003_18 | 9003_693c3 | HQ841703 | - |
| 9003 |  | 9003_19 | 9003_694c8 | HQ841704 | HQ842429 |
| 9003 |  | 9003_19 | 9003_695c7 | HQ841705 | - |
| 9003 |  | 9003_2 | 9003_697c8 | HQ841707 | HQ842430 |
| 9003 |  | 9003_2 | 9003_704c7 | HQ841714 | - |
| 9003 |  | 9003_20 | 9003_698c3 | HQ841708 | HQ842431 |
| 9003 |  | 9003_20 | 9003_699c3 | HQ841709 | - |
| 9003 |  | 9003_21 | 9003_700c5 | HQ841710 | HQ842432 |
| 9003 |  | 9003_21 | 9003_701c3 | HQ841711 | - |
| 9003 |  | 9003_22 | 9003_702c7 | HQ841712 | HQ842433 |
| 9003 |  | 9003_22 | 9003_703c7 | HQ841713 | - |
| 9003 |  | 9003_4 | 9003_705c8 | HQ841715 | HQ842434 |
| 9003 |  | 9003_4 | 9003_706c8 | HQ841716 | - |
| 9003 |  | 9003_5 | 9003_707c8 | HQ841717 | HQ842435 |
| 9003 |  | 9003_5 | 9003_708c8 | HQ841718 | - |
| 9003 |  | 9003_6 | 9003_709c8 | HQ841719 | HQ842436 |
| 9003 |  | 9003_6 | 9003_710c7 | HQ841720 | - |
| 9003 |  | 9003_7 | 9003_711c7 | HQ841721 | HQ842437 |
| 9003 |  | 9003_7 | 9003_712c7 | HQ841722 | - |
| 9003 |  | 9003_8 | 9003_713c8 | HQ841723 | HQ842438 |
| 9003 |  | 9003_8 | 9003_714c8 | HQ841724 | - |
| 9003 |  | 9003_9 | 9003_715c8 | HQ841725 | HQ842439 |
| 9003 |  | 9003_9 | 9003_716c7 | HQ841726 | - |
| 9004 |  | 9004_1 | 9004_1008c | HQ842045 | HQ842571 |
| 9004 |  | 9004_1 | 9004_1029c8 | HQ842066 | - |
| 9004 |  | 9004_10 | 9004_1009c | HQ842046 | HQ842572 |
| 9004 |  | 9004_10 | 9004_1010c8 | HQ842047 | - |
| 9004 |  | 9004_11 | 9004_1011c3 | HQ842048 | HQ842573 |
| 9004 |  | 9004_11 | 9004_1012c3 | HQ842049 | - |
| 9004 |  | 9004_12 | 9004_1013c5 | HQ842050 | HQ842574 |
| 9004 |  | 9004_12 | 9004_1014c3 | HQ842051 | - |
| 9004 |  | 9004_13 | 9004_1015c5 | HQ842052 | HQ842575 |
| 9004 |  | 9004_13 | 9004_1016c3 | HQ842053 | - |
| 9004 |  | 9004_14 | 9004_1017c5 | HQ842054 | HQ842576 |
| 9004 |  | 9004_14 | 9004_1018c5 | HQ842055 | - |
| 9004 |  | 9004_15 | 9004_1019c5 | HQ842056 | HQ842577 |
| 9004 |  | 9004_15 | 9004_1020c3 | HQ842057 | - |
| 9004 |  | 9004_16 | 9004_1021c3 | HQ842058 | HQ842578 |
| 9004 |  | 9004_16 | 9004_1022c3 | HQ842059 | - |
| 9004 |  | 9004_17 | 9004_1023c8 | HQ842060 | HQ842579 |
| 9004 |  | 9004_17 | 9004_1024c8 | HQ842061 | - |
| 9004 |  | 9004_18 | 9004_1025c5 | HQ842062 | HQ842580 |
| 9004 |  | 9004_18 | 9004_1026c5 | HQ842063 | - |
| 9004 |  | 9004_19 | 9004_1027c5 | HQ842064 | HQ842581 |
| 9004 |  | 9004_19 | 9004_1028c3 | HQ842065 | - |
| 9004 |  | 9004_2 | 9004_1041c8 | HQ842078 | HQ842582 |
| 9004 |  | 9004_2 | 9004_1030c | HQ842067 | - |
| 9004 |  | 9004_20 | 9004_1031c3 | HQ842068 | HQ842583 |
| 9004 |  | 9004_20 | 9004_1032c7 | HQ842069 | - |
| 9004 |  | 9004_21 | 9004_1033c5 | HQ842070 | HQ842584 |
| 9004 |  | 9004_21 | 9004_1034c3 | HQ842071 | - |
| 9004 |  | 9004_22 | 9004_1035c3 | HQ842072 | HQ842585 |
| 9004 |  | 9004_22 | 9004_1036c3 | HQ842073 | - |
| 9004 |  | 9004_23 | 9004_1037c | HQ842074 | HQ842586 |
| 9004 |  | 9004_23 | 9004_1038c8 | HQ842075 | - |
| 9004 |  | 9004_24 | 9004_1039c | HQ842076 | HQ842587 |
| 9004 |  | 9004_24 | 9004_1040c8 | HQ842077 | - |
| 9004 |  | 9004_4 | 9004_1042c5 | HQ842079 | HQ842588 |
| 9004 |  | 9004_4 | 9004_1043c7 | HQ842080 | - |
| 9004 |  | 9004_5 | 9004_1044c5 | HQ842081 | HQ842589 |
| 9004 |  | 9004_5 | 9004_1045c5 | HQ842082 | - |
| 9004 |  | 9004_6 | 9004_1046c5 | HQ842083 | HQ842590 |
| 9004 |  | 9004_6 | 9004_1047c5 | HQ842084 | - |
| 9004 |  | 9004_7 | 9004_1048c5 | HQ842085 | HQ842591 |
| 9004 |  | 9004_7 | 9004_1049c5 | HQ842086 | - |
| 9004 |  | 9004_8 | 9004_1050c5 | HQ842087 | HQ842592 |
| 9004 |  | 9004_8 | 9004_1051c3 | HQ842088 | - |
| 9004 |  | 9004_9 | 9004_1052c | HQ842089 | HQ842593 |
| 9004 |  | 9004_9 | 9004_1053c8 | HQ842090 | - |
| 9005 |  | 9005_1 | 9005_872c2 | HQ841875 | HQ842493 |
| 9005 |  | 9005_1 | 9005_891c2 | HQ841894 | - |
| 9005 |  | 9005_10 | 9005_873c2 | HQ841876 | HQ842494 |
| 9005 |  | 9005_10 | 9005_874c | HQ841877 | - |
| 9005 |  | 9005_11 | 9005_875c2 | HQ841878 | HQ842495 |
| 9005 |  | 9005_11 | 9005_876c2 | HQ841879 | - |
| 9005 |  | 9005_12 | 9005_877c2 | HQ841880 | HQ842496 |
| 9005 |  | 9005_12 | 9005_878c2 | HQ841881 | - |
| 9005 |  | 9005_13 | 9005_879c2 | HQ841882 | HQ842497 |
| 9005 |  | 9005_13 | 9005_880c2 | HQ841883 | - |
| 9005 |  | 9005_14 | 9005_881c2 | HQ841884 | HQ842498 |
| 9005 |  | 9005_14 | 9005_882c2 | HQ841885 | - |
| 9005 |  | 9005_15 | 9005_883c2 | HQ841886 | HQ842499 |
| 9005 |  | 9005_15 | 9005_884c2 | HQ841887 | - |
| 9005 |  | 9005_17 | 9005_885c2 | HQ841888 | HQ842500 |
| 9005 |  | 9005_17 | 9005_886c2 | HQ841889 | - |
| 9005 |  | 9005_18 | 9005_887c2 | HQ841890 | HQ842501 |
| 9005 |  | 9005_18 | 9005_888c2 | HQ841891 | - |
| 9005 |  | 9005_19 | 9005_889c2 | HQ841892 | HQ842502 |
| 9005 |  | 9005_19 | 9005_890c2 | HQ841893 | - |
| 9005 |  | 9005_2 | 9005_901c | HQ841904 | HQ842503 |
| 9005 |  | 9005_2 | 9005_892c2 | HQ841895 | - |
| 9005 |  | 9005_20 | 9005_893c2 | HQ841896 | HQ842504 |
| 9005 |  | 9005_20 | 9005_894c2 | HQ841897 | - |
| 9005 |  | 9005_21 | 9005_895c2 | HQ841898 | HQ842505 |
| 9005 |  | 9005_21 | 9005_896c2 | HQ841899 | - |
| 9005 |  | 9005_22 | 9005_897c2 | HQ841900 | HQ842506 |
| 9005 |  | 9005_22 | 9005_898c2 | HQ841901 | - |
| 9005 |  | 9005_23 | 9005_899c2 | HQ841902 | HQ842507 |
| 9005 |  | 9005_23 | 9005_900c2 | HQ841903 | - |
| 9005 |  | 9005_3 | 9005_902c2 | HQ841905 | HQ842508 |
| 9005 |  | 9005_3 | 9005_903c2 | HQ841906 | - |
| 9005 |  | 9005_4 | 9005_904c2 | HQ841907 | HQ842509 |
| 9005 |  | 9005_4 | 9005_905c2 | HQ841908 | - |
| 9005 |  | 9005_5 | 9005_906c2 | HQ841909 | HQ842510 |
| 9005 |  | 9005_5 | 9005_907c2 | HQ841910 | - |
| 9005 |  | 9005_6 | 9005_908c2 | HQ841911 | HQ842511 |
| 9005 |  | 9005_6 | 9005_909c2 | HQ841912 | - |
| 9005 |  | 9005_7 | 9005_910c2 | HQ841913 | HQ842512 |
| 9005 |  | 9005_7 | 9005_911c2 | HQ841914 | - |
| 9005 |  | 9005_8 | 9005_912c2 | HQ841915 | HQ842513 |
| 9005 |  | 9005_8 | 9005_913c2 | HQ841916 | - |
| 9005 |  | 9005_9 | 9005_914c2 | HQ841917 | HQ842514 |
| 9005 |  | 9005_9 | 9005_915c | HQ841918 | - |
| 9007 | sampling point n°2 | 9007_2_1 | 9007_389c5 | HQ841643 | HQ842141 |
| 9007 | sampling point n°2 | 9007_2_1 | 9007_408c9 | HQ841662 | - |
| 9007 | sampling point n°2 | 9007_2_10 | 9007_390c2 | HQ841644 | HQ842142 |
| 9007 | sampling point n°2 | 9007_2_10 | 9007_391c | HQ841645 | - |
| 9007 | sampling point n°2 | 9007_2_11 | 9007_392c5 | HQ841646 | HQ842143 |
| 9007 | sampling point n°2 | 9007_2_11 | 9007_393c3 | HQ841647 | - |
| 9007 | sampling point n°2 | 9007_2_12 | 9007_394c2 | HQ841648 | HQ842144 |
| 9007 | sampling point n°2 | 9007_2_12 | 9007_395c2 | HQ841649 | - |
| 9007 | sampling point n°2 | 9007_2_14 | 9007_396c2 | HQ841650 | HQ842145 |
| 9007 | sampling point n°2 | 9007_2_14 | 9007_397c2 | HQ841651 | - |
| 9007 | sampling point n°2 | 9007_2_15 | 9007_398c | HQ841652 | HQ842146 |
| 9007 | sampling point n°2 | 9007_2_15 | 9007_399c | HQ841653 | - |
| 9007 | sampling point n°2 | 9007_2_16 | 9007_400c2 | HQ841654 | HQ842147 |
| 9007 | sampling point n°2 | 9007_2_16 | 9007_401c2 | HQ841655 | - |
| 9007 | sampling point n°2 | 9007_2_17 | 9007_402c2 | HQ841656 | HQ842148 |
| 9007 | sampling point n°2 | 9007_2_17 | 9007_403c | HQ841657 | - |
| 9007 | sampling point n°2 | 9007_2_18 | 9007_404c5 | HQ841658 | HQ842149 |
| 9007 | sampling point n°2 | 9007_2_18 | 9007_405c3 | HQ841659 | - |
| 9007 | sampling point n°2 | 9007_2_19 | 9007_406c7 | HQ841660 | HQ842150 |
| 9007 | sampling point n°2 | 9007_2_19 | 9007_407c9 | HQ841661 | - |
| 9007 | sampling point n°2 | 9007_2_2 | 9007_409c3 | HQ841663 | HQ842151 |
| 9007 | sampling point n°2 | 9007_2_2 | 9007_418c3 | HQ841672 | - |
| 9007 | sampling point n°2 | 9007_2_21 | 9007_410c2 | HQ841664 | HQ842152 |
| 9007 | sampling point n°2 | 9007_2_21 | 9007_411c2 | HQ841665 | - |
| 9007 | sampling point n°2 | 9007_2_22 | 9007_412c2 | HQ841666 | HQ842153 |
| 9007 | sampling point n°2 | 9007_2_22 | 9007_413c2 | HQ841667 | - |
| 9007 | sampling point n°2 | 9007_2_23 | 9007_414c3 | HQ841668 | HQ842154 |
| 9007 | sampling point n°2 | 9007_2_23 | 9007_415c3 | HQ841669 | - |
| 9007 | sampling point n°2 | 9007_2_24 | 9007_416c3 | HQ841670 | HQ842155 |
| 9007 | sampling point n°2 | 9007_2_24 | 9007_417c7 | HQ841671 | - |
| 9007 | sampling point n°2 | 9007_2_3 | 9007_419c2 | HQ841673 | HQ842156 |
| 9007 | sampling point n°2 | 9007_2_3 | 9007_420c | HQ841674 | - |
| 9007 | sampling point n°2 | 9007_2_5 | 9007_421c3 | HQ841675 | HQ842157 |
| 9007 | sampling point n°2 | 9007_2_5 | 9007_422c | HQ841676 | - |
| 9007 | sampling point n°2 | 9007_2_6 | 9007_423c3 | HQ841677 | HQ842158 |
| 9007 | sampling point n°2 | 9007_2_6 | 9007_424c9 | HQ841678 | - |
| 9007 | sampling point n°2 | 9007_2_7 | 9007_425c2 | HQ841679 | HQ842159 |
| 9007 | sampling point n°2 | 9007_2_7 | 9007_426c2 | HQ841680 | - |
| 9007 | sampling point n°2 | 9007_2_8 | 9007_427c2 | HQ841681 | HQ842160 |
| 9007 | sampling point n°2 | 9007_2_8 | 9007_428c2 | HQ841682 | - |
| 9007 | sampling point n°2 | 9007_2_9 | 9007_429c | HQ841683 | HQ842161 |
| 9007 | sampling point n°2 | 9007_2_9 | 9007_430c7 | HQ841684 | - |
| 9007 | sampling point n°6 | 9007_6_10 | 9007_357c2 | HQ841614 | HQ842128 |
| 9007 | sampling point n°6 | 9007_6_10 | 9007_358c2 | HQ841627 | - |
| 9007 | sampling point n°6 | 9007_6_11 | 9007_359c7 | HQ841615 | HQ842129 |
| 9007 | sampling point n°6 | 9007_6_11 | 9007_360c9 | HQ841628 | - |
| 9007 | sampling point n°6 | 9007_6_12 | 9007_361c3 | HQ841616 | HQ842130 |
| 9007 | sampling point n°6 | 9007_6_12 | 9007_362c3 | HQ841629 | - |
| 9007 | sampling point n°6 | 9007_6_13 | 9007_363c3 | HQ841617 | HQ842131 |
| 9007 | sampling point n°6 | 9007_6_13 | 9007_364c7 | HQ841630 | - |
| 9007 | sampling point n°6 | 9007_6_14 | 9007_365c2 | HQ841618 | HQ842132 |
| 9007 | sampling point n°6 | 9007_6_14 | 9007_366c | HQ841631 | - |
| 9007 | sampling point n°6 | 9007_6_15 | 9007_367c2 | HQ841619 | HQ842133 |
| 9007 | sampling point n°6 | 9007_6_15 | 9007_368c2 | HQ841632 | - |
| 9007 | sampling point n°6 | 9007_6_16 | 9007_369c2 | HQ841620 | HQ842134 |
| 9007 | sampling point n°6 | 9007_6_16 | 9007_370c2 | HQ841633 | - |
| 9007 | sampling point n°6 | 9007_6_17 | 9007_371c5 | HQ841621 | HQ842135 |
| 9007 | sampling point n°6 | 9007_6_17 | 9007_372c3 | HQ841634 | - |
| 9007 | sampling point n°6 | 9007_6_20 | 9007_373c2 | HQ841622 | HQ842136 |
| 9007 | sampling point n°6 | 9007_6_20 | 9007_374c2 | HQ841635 | - |
| 9007 | sampling point n°6 | 9007_6_21 | 9007_375c2 | HQ841623 | HQ842137 |
| 9007 | sampling point n°6 | 9007_6_21 | 9007_376c2 | HQ841636 | - |
| 9007 | sampling point n°6 | 9007_6_22 | 9007_377c2 | HQ841624 | HQ842138 |
| 9007 | sampling point n°6 | 9007_6_22 | 9007_378c2 | HQ841637 | - |
| 9007 | sampling point n°6 | 9007_6_23 | 9007_379c2 | HQ841625 | HQ842139 |
| 9007 | sampling point n°6 | 9007_6_23 | 9007_380c | HQ841638 | - |
| 9007 | sampling point n°6 | 9007_6_24 | 9007_381c2 | HQ841626 | HQ842140 |
| 9007 | sampling point n°6 | 9007_6_24 | 9007_382c2 | HQ841639 | - |
| 9007 | sampling point n°6 | 9007_6_7 | 9007_383c2 | HQ841611 | HQ842125 |
| 9007 | sampling point n°6 | 9007_6_7 | 9007_384c2 | HQ841640 | - |
| 9007 | sampling point n°6 | 9007_6_8 | 9007_385c2 | HQ841612 | HQ842126 |
| 9007 | sampling point n°6 | 9007_6_8 | 9007_386c2 | HQ841641 | - |
| 9007 | sampling point n°6 | 9007_6_9 | 9007_387c2 | HQ841613 | HQ842127 |
| 9007 | sampling point n°6 | 9007_6_9 | 9007_388c2 | HQ841642 | - |
| 9016 | sampling point n°2 | 9016_2_1 | 9016_16c9 | HQ841560 | HQ842162 |
| 9016 | sampling point n°2 | 9016_2_1 | 9016_1c9 | HQ841545 | - |
| 9016 | sampling point n°2 | 9016_2_10 | 9016_2c2 | HQ841546 | HQ842163 |
| 9016 | sampling point n°2 | 9016_2_10 | 9016_3c | HQ841547 | - |
| 9016 | sampling point n°2 | 9016_2_11 | 9016_4c5 | HQ841548 | HQ842164 |
| 9016 | sampling point n°2 | 9016_2_11 | 9016_5c3 | HQ841549 | - |
| 9016 | sampling point n°2 | 9016_2_14 | 9016_6c3 | HQ841550 | HQ842165 |
| 9016 | sampling point n°2 | 9016_2_14 | 9016_7c7 | HQ841551 | - |
| 9016 | sampling point n°2 | 9016_2_16 | 9016_8c5 | HQ841552 | HQ842166 |
| 9016 | sampling point n°2 | 9016_2_16 | 9016_9c3 | HQ841553 | - |
| 9016 | sampling point n°2 | 9016_2_17 | 9016_10c3 | HQ841554 | HQ842167 |
| 9016 | sampling point n°2 | 9016_2_17 | 9016_11c3 | HQ841555 | - |
| 9016 | sampling point n°2 | 9016_2_18 | 9016_12c5 | HQ841556 | HQ842168 |
| 9016 | sampling point n°2 | 9016_2_18 | 9016_13c3 | HQ841557 | - |
| 9016 | sampling point n°2 | 9016_2_19 | 9016_14c3 | HQ841558 | HQ842169 |
| 9016 | sampling point n°2 | 9016_2_19 | 9016_15c9 | HQ841559 | - |
| 9016 | sampling point n°2 | 9016_2_2 | 9016_17c3 | HQ841561 | HQ842170 |
| 9016 | sampling point n°2 | 9016_2_2 | 9016_24c3 | HQ841568 | - |
| 9016 | sampling point n°2 | 9016_2_20 | 9016_18c3 | HQ841562 | HQ842171 |
| 9016 | sampling point n°2 | 9016_2_20 | 9016_19c9 | HQ841563 | - |
| 9016 | sampling point n°2 | 9016_2_21 | 9016_20c5 | HQ841564 | HQ842172 |
| 9016 | sampling point n°2 | 9016_2_21 | 9016_21c3 | HQ841565 | - |
| 9016 | sampling point n°2 | 9016_2_22 | 9016_22c3 | HQ841566 | HQ842173 |
| 9016 | sampling point n°2 | 9016_2_22 | 9016_23c7 | HQ841567 | - |
| 9016 | sampling point n°2 | 9016_2_5 | 9016_25c7 | HQ841569 | HQ842174 |
| 9016 | sampling point n°2 | 9016_2_5 | 9016_26c9 | HQ841570 | - |
| 9016 | sampling point n°2 | 9016_2_7 | 9016_27c3 | HQ841571 | HQ842175 |
| 9016 | sampling point n°2 | 9016_2_7 | 9016_28c7 | HQ841572 | - |
| 9016 | sampling point n°2 | 9016_2_8 | 9016_29c7 | HQ841573 | HQ842176 |
| 9016 | sampling point n°2 | 9016_2_8 | 9016_30c9 | HQ841574 | - |
| 9016 | sampling point n°2 | 9016_2_9 | 9016_31c3 | HQ841575 | HQ842177 |
| 9016 | sampling point n°2 | 9016_2_9 | 9016_32c9 | HQ841576 | - |
| 9016 | sampling point n°6 | 9016_6_1 | 9016_33c3 | HQ841577 | HQ842178 |
| 9016 | sampling point n°6 | 9016_6_1 | 9016_50c9 | HQ841594 | - |
| 9016 | sampling point n°6 | 9016_6_10 | 9016_34c3 | HQ841578 | HQ842179 |
| 9016 | sampling point n°6 | 9016_6_10 | 9016_35c9 | HQ841579 | - |
| 9016 | sampling point n°6 | 9016_6_11 | 9016_36c3 | HQ841580 | HQ842180 |
| 9016 | sampling point n°6 | 9016_6_11 | 9016_37c | HQ841581 | - |
| 9016 | sampling point n°6 | 9016_6_12 | 9016_38c5 | HQ841582 | HQ842181 |
| 9016 | sampling point n°6 | 9016_6_12 | 9016_39c7 | HQ841583 | - |
| 9016 | sampling point n°6 | 9016_6_14 | 9016_40c3 | HQ841584 | HQ842182 |
| 9016 | sampling point n°6 | 9016_6_14 | 9016_41c7 | HQ841585 | - |
| 9016 | sampling point n°6 | 9016_6_15 | 9016_42c3 | HQ841586 | HQ842183 |
| 9016 | sampling point n°6 | 9016_6_15 | 9016_43c9 | HQ841587 | - |
| 9016 | sampling point n°6 | 9016_6_16 | 9016_44c | HQ841588 | HQ842184 |
| 9016 | sampling point n°6 | 9016_6_16 | 9016_45c | HQ841589 | - |
| 9016 | sampling point n°6 | 9016_6_17 | 9016_46c3 | HQ841590 | HQ842185 |
| 9016 | sampling point n°6 | 9016_6_17 | 9016_47c3 | HQ841591 | - |
| 9016 | sampling point n°6 | 9016_6_19 | 9016_48c3 | HQ841592 | HQ842186 |
| 9016 | sampling point n°6 | 9016_6_19 | 9016_49c7 | HQ841593 | - |
| 9016 | sampling point n°6 | 9016_6_2 | 9016_51c5 | HQ841595 | HQ842187 |
| 9016 | sampling point n°6 | 9016_6_2 | 9016_54c9 | HQ841598 | - |
| 9016 | sampling point n°6 | 9016_6_20 | 9016_52c2 | HQ841596 | HQ842188 |
| 9016 | sampling point n°6 | 9016_6_20 | 9016_53c2 | HQ841597 | - |
| 9016 | sampling point n°6 | 9016_6_3 | 9016_55c3 | HQ841599 | HQ842189 |
| 9016 | sampling point n°6 | 9016_6_3 | 9016_56c7 | HQ841600 | - |
| 9016 | sampling point n°6 | 9016_6_5 | 9016_57c7 | HQ841601 | HQ842190 |
| 9016 | sampling point n°6 | 9016_6_5 | 9016_58c9 | HQ841602 | - |
| 9016 | sampling point n°6 | 9016_6_6 | 9016_59c3 | HQ841603 | HQ842191 |
| 9016 | sampling point n°6 | 9016_6_6 | 9016_60c7 | HQ841604 | - |
| 9016 | sampling point n°6 | 9016_6_7 | 9016_61c3 | HQ841605 | HQ842192 |
| 9016 | sampling point n°6 | 9016_6_7 | 9016_62c3 | HQ841606 | - |
| 9016 | sampling point n°6 | 9016_6_8 | 9016_63c5 | HQ841607 | HQ842193 |
| 9016 | sampling point n°6 | 9016_6_8 | 9016_64c7 | HQ841608 | - |
| 9016 | sampling point n°6 | 9016_6_9 | 9016_65c3 | HQ841609 | HQ842194 |
| 9016 | sampling point n°6 | 9016_6_9 | 9016_66c3 | HQ841610 | - |
| AUS |  | AUS1 | AUS_431c5 | HQ841229 | HQ842340 |
| AUS |  | AUS1 | AUS_452c5 | HQ841250 | - |
| AUS |  | AUS10 | AUS_432c5 | HQ841230 | HQ842341 |
| AUS |  | AUS10 | AUS_433c7 | HQ841231 | - |
| AUS |  | AUS11 | AUS_434c5 | HQ841232 | HQ842342 |
| AUS |  | AUS11 | AUS_435c5 | HQ841233 | - |
| AUS |  | AUS12 | AUS_436c5 | HQ841234 | HQ842343 |
| AUS |  | AUS12 | AUS_437c5 | HQ841235 | - |
| AUS |  | AUS13 | AUS_438c5 | HQ841236 | HQ842344 |
| AUS |  | AUS13 | AUS_439c5 | HQ841237 | - |
| AUS |  | AUS14 | AUS_440c5 | HQ841238 | HQ842345 |
| AUS |  | AUS14 | AUS_441c5 | HQ841239 | - |
| AUS |  | AUS15 | AUS_442c5 | HQ841240 | HQ842346 |
| AUS |  | AUS15 | AUS_443c7 | HQ841241 | - |
| AUS |  | AUS16 | AUS_444c5 | HQ841242 | HQ842347 |
| AUS |  | AUS16 | AUS_445c5 | HQ841243 | - |
| AUS |  | AUS17 | AUS_446c5 | HQ841244 | HQ842348 |
| AUS |  | AUS17 | AUS_447c5 | HQ841245 | - |
| AUS |  | AUS18 | AUS_448c5 | HQ841246 | HQ842349 |
| AUS |  | AUS18 | AUS_449c5 | HQ841247 | - |
| AUS |  | AUS19 | AUS_450c5 | HQ841248 | HQ842350 |
| AUS |  | AUS19 | AUS_451c5 | HQ841249 | - |
| AUS |  | AUS2 | AUS_464c5 | HQ841262 | HQ842351 |
| AUS |  | AUS2 | AUS_453c5 | HQ841251 | - |
| AUS |  | AUS20 | AUS_454c5 | HQ841252 | HQ842352 |
| AUS |  | AUS20 | AUS_455c5 | HQ841253 | - |
| AUS |  | AUS21 | AUS_456c5 | HQ841254 | HQ842353 |
| AUS |  | AUS21 | AUS_457c5 | HQ841255 | - |
| AUS |  | AUS22 | AUS_458c5 | HQ841256 | HQ842354 |
| AUS |  | AUS22 | AUS_459c5 | HQ841257 | - |
| AUS |  | AUS23 | AUS_460c5 | HQ841258 | HQ842355 |
| AUS |  | AUS23 | AUS_461c5 | HQ841259 | - |
| AUS |  | AUS24 | AUS_462c5 | HQ841260 | HQ842356 |
| AUS |  | AUS24 | AUS_463c5 | HQ841261 | - |
| AUS |  | AUS3 | AUS_465c5 | HQ841263 | HQ842357 |
| AUS |  | AUS3 | AUS_466c5 | HQ841264 | - |
| AUS |  | AUS4 | AUS_467c5 | HQ841265 | HQ842358 |
| AUS |  | AUS4 | AUS_468c5 | HQ841266 | - |
| AUS |  | AUS5 | AUS_469c5 | HQ841267 | HQ842359 |
| AUS |  | AUS5 | AUS_470c5 | HQ841268 | - |
| AUS |  | AUS6 | AUS_471c5 | HQ841269 | HQ842360 |
| AUS |  | AUS6 | AUS_472c5 | HQ841270 | - |
| AUS |  | AUS7 | AUS_473c5 | HQ841271 | HQ842361 |
| AUS |  | AUS7 | AUS_474c5 | HQ841272 | - |
| AUS |  | AUS8 | AUS_475c5 | HQ841273 | HQ842362 |
| AUS |  | AUS8 | AUS_476c5 | HQ841274 | - |
| AUS |  | AUS9 | AUS_477c5 | HQ841275 | HQ842363 |
| AUS |  | AUS9 | AUS_478c5 | HQ841276 | - |
| BOUY |  | BOUY14 | BOUY_521c5 | HQ841391 | HQ842364 |
| BOUY |  | BOUY14 | BOUY_522c5 | HQ841392 | - |
| BOUY |  | BOUY15 | BOUY_523c8 | HQ841393 | HQ842365 |
| BOUY |  | BOUY15 | BOUY_524c8 | HQ841394 | - |
| BOUY |  | BOUY17 | BOUY_525c8 | HQ841395 | HQ842366 |
| BOUY |  | BOUY17 | BOUY_526c8 | HQ841396 | - |
| BOUY |  | BOUY18 | BOUY_527c5 | HQ841397 | HQ842367 |
| BOUY |  | BOUY18 | BOUY_528c8 | HQ841398 | - |
| BOUY |  | BOUY20 | BOUY_529c8 | HQ841399 | HQ842368 |
| BOUY |  | BOUY20 | BOUY_530c8 | HQ841400 | - |
| BOUY |  | BOUY21 | BOUY_531c5 | HQ841401 | HQ842369 |
| BOUY |  | BOUY21 | BOUY_532c5 | HQ841402 | - |
| BOUY |  | BOUY22 | BOUY_533c5 | HQ841403 | HQ842370 |
| BOUY |  | BOUY22 | BOUY_534c8 | HQ841404 | - |
| BOUY |  | BOUY23 | BOUY_535c8 | HQ841405 | HQ842371 |
| BOUY |  | BOUY23 | BOUY_536c7 | HQ841406 | - |
| BOUY |  | BOUY24 | BOUY_537c8 | HQ841407 | HQ842372 |
| BOUY |  | BOUY24 | BOUY_538c7 | HQ841408 | - |
| BOUY |  | BOUY25 | BOUY_539c5 | HQ841409 | HQ842373 |
| BOUY |  | BOUY25 | BOUY_540c5 | HQ841410 | - |
| BOUY |  | BOUY26 | BOUY_541c5 | HQ841411 | HQ842374 |
| BOUY |  | BOUY26 | BOUY_542c7 | HQ841412 | - |
| BOUY |  | BOUY27 | BOUY_543c7 | HQ841413 | HQ842375 |
| BOUY |  | BOUY27 | BOUY_544c7 | HQ841414 | - |
| BOUY |  | BOUY28 | BOUY_545c5 | HQ841415 | HQ842376 |
| BOUY |  | BOUY28 | BOUY_546c8 | HQ841416 | - |
| BOUY |  | BOUY30 | BOUY_547c3 | HQ841417 | HQ842377 |
| BOUY |  | BOUY30 | BOUY_548c7 | HQ841418 | - |
| BOUY |  | BOUY31 | BOUY_549c8 | HQ841419 | HQ842378 |
| BOUY |  | BOUY31 | BOUY_550c8 | HQ841420 | - |
| BOUY |  | BOUY32 | BOUY_551c | HQ841421 | HQ842379 |
| BOUY |  | BOUY32 | BOUY_552c | HQ841422 | - |
| BOUY |  | BOUY33 | BOUY_553c5 | HQ841423 | HQ842380 |
| BOUY |  | BOUY33 | BOUY_554c8 | HQ841424 | - |
| BREa |  | BRE1 | BREa_555c7 | HQ841425 | HQ842381 |
| BREa |  | BRE1 | BREa_566c7 | HQ841436 | - |
| BREa |  | BRE11 | BREa_556c5 | HQ841426 | HQ842382 |
| BREa |  | BRE11 | BREa_557c | HQ841427 | - |
| BREa |  | BRE13 | BREa_558c7 | HQ841428 | HQ842383 |
| BREa |  | BRE13 | BREa_559c7 | HQ841429 | - |
| BREa |  | BRE16 | BREa_560c7 | HQ841430 | HQ842384 |
| BREa |  | BRE16 | BREa_561c | HQ841431 | - |
| BREa |  | BRE18 | BREa_562c7 | HQ841432 | HQ842385 |
| BREa |  | BRE18 | BREa_563c7 | HQ841433 | - |
| BREa |  | BRE19 | BREa_564c7 | HQ841434 | HQ842386 |
| BREa |  | BRE19 | BREa_565c7 | HQ841435 | - |
| BREa |  | BRE2 | BREa_578c | HQ841448 | HQ842387 |
| BREa |  | BRE2 | BREa_567c7 | HQ841437 | - |
| BREa |  | BRE21 | BREa_568c7 | HQ841438 | HQ842388 |
| BREa |  | BRE21 | BREa_569c7 | HQ841439 | - |
| BREa |  | BRE22 | BREa_570c7 | HQ841440 | HQ842389 |
| BREa |  | BRE22 | BREa_571c | HQ841441 | - |
| BREa |  | BRE23 | BREa_572c5 | HQ841442 | HQ842390 |
| BREa |  | BRE23 | BREa_573c7 | HQ841443 | - |
| BREa |  | BRE25 | BREa_574c7 | HQ841444 | HQ842391 |
| BREa |  | BRE25 | BREa_575c | HQ841445 | - |
| BREa |  | BRE26 | BREa_576c5 | HQ841446 | HQ842392 |
| BREa |  | BRE26 | BREa_577c7 | HQ841447 | - |
| BREa |  | BRE3 | BREa_582c7 | HQ841452 | HQ842393 |
| BREa |  | BRE3 | BREa_579c7 | HQ841449 | - |
| BREa |  | BRE30 | BREa_580c5 | HQ841450 | HQ842394 |
| BREa |  | BRE30 | BREa_581c7 | HQ841451 | - |
| BREa |  | BRE4 | BREa_583c5 | HQ841453 | HQ842395 |
| BREa |  | BRE4 | BREa_584c7 | HQ841454 | - |
| BREa |  | BRE5 | BREa_585c7 | HQ841455 | HQ842396 |
| BREa |  | BRE5 | BREa_586c7 | HQ841456 | - |
| BREa |  | BRE6 | BREa_587c7 | HQ841457 | HQ842397 |
| BREa |  | BRE6 | BREa_588c7 | HQ841458 | - |
| BREa |  | BRE7 | BREa_589c7 | HQ841459 | HQ842398 |
| BREa |  | BRE7 | BREa_590c7 | HQ841460 | - |
| BREa |  | BRE8 | BREa_591c7 | HQ841461 | HQ842399 |
| BREa |  | BRE8 | BREa_592c7 | HQ841462 | - |
| BREa |  | BRE9 | BREa_593c7 | HQ841463 | HQ842400 |
| BREa |  | BRE9 | BREa_594c | HQ841464 | - |
| BREb |  | BREb1 | BREb_595c | HQ841465 | HQ842401 |
| BREb |  | BREb1 | BREb_612c | HQ841482 | - |
| BREb |  | BREb10 | BREb_596c7 | HQ841466 | HQ842402 |
| BREb |  | BREb10 | BREb_597c7 | HQ841467 | - |
| BREb |  | BREb11 | BREb_598c7 | HQ841468 | HQ842403 |
| BREb |  | BREb11 | BREb_599c7 | HQ841469 | - |
| BREb |  | BREb12 | BREb_600c7 | HQ841470 | HQ842404 |
| BREb |  | BREb12 | BREb_601c7 | HQ841471 | - |
| BREb |  | BREb14 | BREb_602c7 | HQ841472 | HQ842405 |
| BREb |  | BREb14 | BREb_603c7 | HQ841473 | - |
| BREb |  | BREb15 | BREb_604c7 | HQ841474 | HQ842406 |
| BREb |  | BREb15 | BREb_605c7 | HQ841475 | - |
| BREb |  | BREb17 | BREb_606c7 | HQ841476 | HQ842407 |
| BREb |  | BREb17 | BREb_607c7 | HQ841477 | - |
| BREb |  | BREb18 | BREb_608c7 | HQ841478 | HQ842408 |
| BREb |  | BREb18 | BREb_609c7 | HQ841479 | - |
| BREb |  | BREb19 | BREb_610c7 | HQ841480 | HQ842409 |
| BREb |  | BREb19 | BREb_611c7 | HQ841481 | - |
| BREb |  | BREb2 | BREb_618c7 | HQ841488 | HQ842410 |
| BREb |  | BREb2 | BREb_613c7 | HQ841483 | - |
| BREb |  | BREb21 | BREb_614c7 | HQ841484 | HQ842411 |
| BREb |  | BREb21 | BREb_615c7 | HQ841485 | - |
| BREb |  | BREb22 | BREb_616c7 | HQ841486 | HQ842412 |
| BREb |  | BREb22 | BREb_617c7 | HQ841487 | - |
| BREb |  | BREb3 | BREb_619c7 | HQ841489 | HQ842413 |
| BREb |  | BREb3 | BREb_620c7 | HQ841490 | - |
| BREb |  | BREb4 | BREb_621c7 | HQ841491 | HQ842414 |
| BREb |  | BREb4 | BREb_622c7 | HQ841492 | - |
| BREb |  | BREb5 | BREb_623c7 | HQ841493 | HQ842415 |
| BREb |  | BREb5 | BREb_624c7 | HQ841494 | - |
| BREb |  | BREb6 | BREb_625c7 | HQ841495 | HQ842416 |
| BREb |  | BREb6 | BREb_626c7 | HQ841496 | - |
| BREb |  | BREb7 | BREb_627c7 | HQ841497 | HQ842417 |
| BREb |  | BREb7 | BREb_628c7 | HQ841498 | - |
| BREb |  | BREb8 | BREb_629c7 | HQ841499 | HQ842418 |
| BREb |  | BREb8 | BREb_630c7 | HQ841500 | - |
| IL |  | IL202A | IL_734c | HQ841727 | HQ842440 |
| IL |  | IL202A | IL_735c1 | HQ841728 | - |
| IL |  | IL202C | IL_736c | HQ841729 | HQ842441 |
| IL |  | IL202C | IL_737c | HQ841730 | - |
| IL |  | IL202o | IL_738c | HQ841731 | HQ842442 |
| IL |  | IL202o | IL_739c1 | HQ841732 | - |
| IL |  | IL202p | IL_740c1 | HQ841733 | HQ842443 |
| IL |  | IL202p | IL_741c1 | HQ841734 | - |
| IL |  | IL202q | IL_742c | HQ841735 | HQ842444 |
| IL |  | IL202q | IL_743c | HQ841736 | - |
| IL |  | IL2132 | IL_744c1 | HQ841737 | HQ842445 |
| IL |  | IL2132 | IL_745c1 | HQ841738 | - |
| IL |  | IL2133 | IL_746c1 | HQ841739 | HQ842446 |
| IL |  | IL2133 | IL_747c1 | HQ841740 | - |
| IL |  | IL2272 | IL_748c1 | HQ841741 | HQ842447 |
| IL |  | IL2272 | IL_749c1 | HQ841742 | - |
| IL |  | IL2273 | IL_750c1 | HQ841743 | HQ842448 |
| IL |  | IL2273 | IL_751c | HQ841744 | - |
| IL |  | IL302f | IL_752c1 | HQ841745 | HQ842449 |
| IL |  | IL302f | IL_753c | HQ841746 | - |
| IL |  | IL302h | IL_754c | HQ841747 | HQ842450 |
| IL |  | IL302h | IL_755c1 | HQ841748 | - |
| IL |  | IL302m | IL_756c1 | HQ841749 | HQ842451 |
| IL |  | IL302m | IL_757c | HQ841750 | - |
| IL |  | IL302n | IL_758c | HQ841751 | HQ842452 |
| IL |  | IL302n | IL_759c1 | HQ841752 | - |
| IL |  | IL302o | IL_760c | HQ841753 | HQ842453 |
| IL |  | IL302o | IL_761c1 | HQ841754 | - |
| IL |  | IL302p | IL_762c | HQ841755 | HQ842454 |
| IL |  | IL302p | IL_763c | HQ841756 | - |
| IL |  | IL302q | IL_764c | HQ841757 | HQ842455 |
| IL |  | IL302q | IL_765c | HQ841758 | - |
| L1FR |  | L1FR_CANIM | L1FR_631c4 | HQ842099 | HQ842568 |
| L1FR |  | L1FR_CANIM | L1FR_632c4 | HQ842100 | - |
| L1FR |  | L1FR_COL1 | L1FR_633c4 | HQ842101 | HQ842569 |
| L1FR |  | L1FR_COL1 | L1FR_634c4 | HQ842102 | - |
| L1FR |  | L1FR_COL3 | L1FR_635c4 | HQ842103 | HQ842570 |
| L1FR |  | L1FR_COL3 | L1FR_636c4 | HQ842104 | - |
| L1FR |  | L1FR_LC1 | L1FR_779c4 | HQ842091 | HQ842566 |
| L1FR |  | L1FR_LC1 | L1FR_780c4 | HQ842092 | - |
| L1FR |  | L1FR_LC4 | L1FR_781c4 | HQ842093 | HQ842567 |
| L1FR |  | L1FR_LC4 | L1FR_782c4 | HQ842094 | - |
| L1FR |  | L1FR_Pl1 | L1FR_794c4 | HQ842095 | HQ842564 |
| L1FR |  | L1FR_Pl1 | L1FR_795c4 | HQ842096 | - |
| L1FR |  | L1FR_Pl2 | L1FR_796c4 | HQ842097 | HQ842565 |
| L1FR |  | L1FR_Pl2 | L1FR_797c4 | HQ842098 | - |
| L1US |  | L1US_1231 | L1US_998c4 | HQ842115 | HQ842554 |
| L1US |  | L1US_1231 | L1US_999c4 | HQ842116 | - |
| L1US |  | L1US_1232 | L1US_1000c4 | HQ842117 | HQ842555 |
| L1US |  | L1US_1232 | L1US_1001c4 | HQ842118 | - |
| L1US |  | L1US_1233 | L1US_1002c4 | HQ842119 | HQ842556 |
| L1US |  | L1US_1233 | L1US_1003c4 | HQ842120 | - |
| L1US |  | L1US_1234 | L1US_1004c4 | HQ842121 | HQ842557 |
| L1US |  | L1US_1234 | L1US_1005c4 | HQ842122 | - |
| L1US |  | L1US_1235 | L1US_1006c4 | HQ842123 | HQ842558 |
| L1US |  | L1US_1235 | L1US_1007c4 | HQ842124 | - |
| L1FR |  | L1US_N331 | L1US_784c4 | HQ842105 | HQ842563 |
| L1FR |  | L1US_N331 | L1US_785c4 | HQ842106 | - |
| L1FR |  | L1US_N332 | L1US_786c4 | HQ842107 | HQ842559 |
| L1FR |  | L1US_N332 | L1US_787c4 | HQ842108 | - |
| L1FR |  | L1US_N333 | L1US_788c4 | HQ842109 | HQ842560 |
| L1FR |  | L1US_N333 | L1US_789c4 | HQ842110 | - |
| L1FR |  | L1US_N334 | L1US_790c4 | HQ842111 | HQ842561 |
| L1FR |  | L1US_N334 | L1US_791c4 | HQ842112 | - |
| L1FR |  | L1US_N335 | L1US_792c4 | HQ842113 | HQ842562 |
| L1FR |  | L1US_N335 | L1US_793c4 | HQ842114 | - |
| PO2 |  | PO2A | PO2_798c6 | HQ841759 | HQ842456 |
| PO2 |  | PO2A | PO2_799c6 | HQ841760 | - |
| PO2 |  | PO2B | PO2_800c | HQ841761 | HQ842457 |
| PO2 |  | PO2B | PO2_801c6 | HQ841762 | - |
| PO2 |  | PO2g | PO2_802c | HQ841763 | HQ842458 |
| PO2 |  | PO2g | PO2_803c6 | HQ841764 | - |
| PO2 |  | PO2h | PO2_804c6 | HQ841765 | HQ842459 |
| PO2 |  | PO2h | PO2_805c6 | HQ841766 | - |
| PO2 |  | PO2j | PO2_806c6 | HQ841767 | HQ842460 |
| PO2 |  | PO2j | PO2_807c6 | HQ841768 | - |
| PO2 |  | PO2k | PO2_808c | HQ841769 | HQ842461 |
| PO2 |  | PO2k | PO2_809c6 | HQ841770 | - |
| PO2 |  | PO2n | PO2_810c6 | HQ841771 | HQ842462 |
| PO2 |  | PO2n | PO2_811c6 | HQ841772 | - |
| PO2 |  | PO2o | PO2_812c6 | HQ841773 | HQ842463 |
| PO2 |  | PO2o | PO2_813c6 | HQ841774 | - |
| PO2 |  | PO2p | PO2_814c6 | HQ841775 | HQ842464 |
| PO2 |  | PO2p | PO2_815c6 | HQ841776 | - |
| PO2 |  | PO2q | PO2_816c | HQ841777 | HQ842465 |
| PO2 |  | PO2q | PO2_817c6 | HQ841778 | - |
| PO2 |  | PO2r | PO2_818c6 | HQ841779 | HQ842466 |
| PO2 |  | PO2r | PO2_819c6 | HQ841780 | - |
| PO2 |  | PO2s | PO2_820c | HQ841781 | HQ842467 |
| PO2 |  | PO2s | PO2_821c6 | HQ841782 | - |
| PO2 |  | PO2t | PO2_822c6 | HQ841783 | HQ842468 |
| PO2 |  | PO2t | PO2_823c6 | HQ841784 | - |
| PO2 |  | PO2u | PO2_824c6 | HQ841785 | HQ842469 |
| PO2 |  | PO2u | PO2_825c6 | HQ841786 | - |
| PO2 |  | PO2w | PO2_826c6 | HQ841787 | HQ842470 |
| PO2 |  | PO2w | PO2_827c6 | HQ841788 | - |
| PO2 |  | PO2x | PO2_828c6 | HQ841789 | HQ842471 |
| PO2 |  | PO2x | PO2_829c6 | HQ841790 | - |
| PO2 |  | PO2y | PO2_830c6 | HQ841791 | HQ842472 |
| PO2 |  | PO2y | PO2_831c6 | HQ841792 | - |
| REN |  | REN1 | REN_832c2 | HQ841835 | HQ842473 |
| REN |  | REN1 | REN_853c2 | HQ841856 | - |
| REN |  | REN10 | REN_833c2 | HQ841836 | HQ842474 |
| REN |  | REN10 | REN_834c2 | HQ841837 | - |
| REN |  | REN11 | REN_835c2 | HQ841838 | HQ842475 |
| REN |  | REN11 | REN_836c2 | HQ841839 | - |
| REN |  | REN12 | REN_837c2 | HQ841840 | HQ842476 |
| REN |  | REN12 | REN_838c2 | HQ841841 | - |
| REN |  | REN13 | REN_839c2 | HQ841842 | HQ842477 |
| REN |  | REN13 | REN_840c2 | HQ841843 | - |
| REN |  | REN14 | REN_841c2 | HQ841844 | HQ842478 |
| REN |  | REN14 | REN_842c2 | HQ841845 | - |
| REN |  | REN15 | REN_843c2 | HQ841846 | HQ842479 |
| REN |  | REN15 | REN_844c2 | HQ841847 | - |
| REN |  | REN16 | REN_845c2 | HQ841848 | HQ842480 |
| REN |  | REN16 | REN_846c2 | HQ841849 | - |
| REN |  | REN17 | REN_847c2 | HQ841850 | HQ842481 |
| REN |  | REN17 | REN_848c2 | HQ841851 | - |
| REN |  | REN18 | REN_849c2 | HQ841852 | HQ842482 |
| REN |  | REN18 | REN_850c2 | HQ841853 | - |
| REN |  | REN19 | REN_851c2 | HQ841854 | HQ842483 |
| REN |  | REN19 | REN_852c2 | HQ841855 | - |
| REN |  | REN2 | REN_863c2 | HQ841866 | HQ842484 |
| REN |  | REN2 | REN_854c2 | HQ841857 | - |
| REN |  | REN20 | REN_855c2 | HQ841858 | HQ842485 |
| REN |  | REN20 | REN_856c2 | HQ841859 | - |
| REN |  | REN21 | REN_857c2 | HQ841860 | HQ842486 |
| REN |  | REN21 | REN_858c2 | HQ841861 | - |
| REN |  | REN23 | REN_859c2 | HQ841862 | HQ842487 |
| REN |  | REN23 | REN_860c2 | HQ841863 | - |
| REN |  | REN24 | REN_861c2 | HQ841864 | HQ842488 |
| REN |  | REN24 | REN_862c2 | HQ841865 | - |
| REN |  | REN3 | REN_864c2 | HQ841867 | HQ842489 |
| REN |  | REN3 | REN_865c2 | HQ841868 | - |
| REN |  | REN4 | REN_866c2 | HQ841869 | HQ842490 |
| REN |  | REN4 | REN_867c2 | HQ841870 | - |
| REN |  | REN6 | REN_868c2 | HQ841871 | HQ842491 |
| REN |  | REN6 | REN_869c2 | HQ841872 | - |
| REN |  | REN7 | REN_870c2 | HQ841873 | HQ842492 |
| REN |  | REN7 | REN_871c2 | HQ841874 | - |
| ROL |  | ROL091 | ROL_916c1 | HQ841919 | HQ842515 |
| ROL |  | ROL091 | ROL_933c1 | HQ841936 | - |
| ROL |  | ROL0911 | ROL_917c1 | HQ841920 | HQ842516 |
| ROL |  | ROL0911 | ROL_918c | HQ841921 | - |
| ROL |  | ROL0912 | ROL_919c1 | HQ841922 | HQ842517 |
| ROL |  | ROL0912 | ROL_920c1 | HQ841923 | - |
| ROL |  | ROL0913 | ROL_921c1 | HQ841924 | HQ842518 |
| ROL |  | ROL0913 | ROL_922c1 | HQ841925 | - |
| ROL |  | ROL0914 | ROL_923c1 | HQ841926 | HQ842519 |
| ROL |  | ROL0914 | ROL_924c | HQ841927 | - |
| ROL |  | ROL0915 | ROL_925c1 | HQ841928 | HQ842520 |
| ROL |  | ROL0915 | ROL_926c1 | HQ841929 | - |
| ROL |  | ROL0916 | ROL_927c1 | HQ841930 | HQ842521 |
| ROL |  | ROL0916 | ROL_928c1 | HQ841931 | - |
| ROL |  | ROL0918 | ROL_929c1 | HQ841932 | HQ842522 |
| ROL |  | ROL0918 | ROL_930c | HQ841933 | - |
| ROL |  | ROL0919 | ROL_931c1 | HQ841934 | HQ842523 |
| ROL |  | ROL0919 | ROL_932c1 | HQ841935 | - |
| ROL |  | ROL092 | ROL_939c1 | HQ841942 | HQ842524 |
| ROL |  | ROL092 | ROL_934c1 | HQ841937 | - |
| ROL |  | ROL0920 | ROL_935c | HQ841938 | HQ842525 |
| ROL |  | ROL0920 | ROL_936c1 | HQ841939 | - |
| ROL |  | ROL0921 | ROL_937c | HQ841940 | HQ842526 |
| ROL |  | ROL0921 | ROL_938c1 | HQ841941 | - |
| ROL |  | ROL093 | ROL_940c1 | HQ841943 | HQ842527 |
| ROL |  | ROL093 | ROL_941c | HQ841944 | - |
| ROL |  | ROL094 | ROL_942c1 | HQ841945 | HQ842528 |
| ROL |  | ROL094 | ROL_943c | HQ841946 | - |
| ROL |  | ROL095 | ROL_944c1 | HQ841947 | HQ842529 |
| ROL |  | ROL095 | ROL_945c1 | HQ841948 | - |
| ROL |  | ROL097 | ROL_946c1 | HQ841949 | HQ842530 |
| ROL |  | ROL097 | ROL_947c1 | HQ841950 | - |
| ROL |  | ROL098 | ROL_948c | HQ841951 | HQ842531 |
| ROL |  | ROL098 | ROL_949c1 | HQ841952 | - |
| ROL |  | ROL099 | ROL_950c1 | HQ841953 | HQ842532 |
| ROL |  | ROL099 | ROL_951c1 | HQ841954 | - |
| SK |  | SK081 | SK_956c | HQ842003 | HQ842533 |
| SK |  | SK081 | SK_977c3 | HQ842024 | - |
| SK |  | SK0810 | SK_957c | HQ842004 | HQ842534 |
| SK |  | SK0810 | SK_958c3 | HQ842005 | - |
| SK |  | SK0811 | SK_959c3 | HQ842006 | HQ842535 |
| SK |  | SK0811 | SK_960c3 | HQ842007 | - |
| SK |  | SK0812 | SK_961c | HQ842008 | HQ842536 |
| SK |  | SK0812 | SK_962c3 | HQ842009 | - |
| SK |  | SK0813 | SK_963c | HQ842010 | HQ842537 |
| SK |  | SK0813 | SK_964c | HQ842011 | - |
| SK |  | SK0814 | SK_965c | HQ842012 | HQ842538 |
| SK |  | SK0814 | SK_966c | HQ842013 | - |
| SK |  | SK0815 | SK_967c3 | HQ842014 | HQ842539 |
| SK |  | SK0815 | SK_968c3 | HQ842015 | - |
| SK |  | SK0816 | SK_969c | HQ842016 | HQ842540 |
| SK |  | SK0816 | SK_970c3 | HQ842017 | - |
| SK |  | SK0817 | SK_971c3 | HQ842018 | HQ842541 |
| SK |  | SK0817 | SK_972c3 | HQ842019 | - |
| SK |  | SK0818 | SK_973c3 | HQ842020 | HQ842542 |
| SK |  | SK0818 | SK_974c3 | HQ842021 | - |
| SK |  | SK0819 | SK_975c3 | HQ842022 | HQ842543 |
| SK |  | SK0819 | SK_976c3 | HQ842023 | - |
| SK |  | SK082 | SK_987c3 | HQ842034 | HQ842544 |
| SK |  | SK082 | SK_978c | HQ842025 | - |
| SK |  | SK0820 | SK_979c3 | HQ842026 | HQ842545 |
| SK |  | SK0820 | SK_980c3 | HQ842027 | - |
| SK |  | SK0821 | SK_981c3 | HQ842028 | HQ842546 |
| SK |  | SK0821 | SK_982c3 | HQ842029 | - |
| SK |  | SK0822 | SK_983c3 | HQ842030 | HQ842547 |
| SK |  | SK0822 | SK_984c3 | HQ842031 | - |
| SK |  | SK0824 | SK_985c | HQ842032 | HQ842548 |
| SK |  | SK0824 | SK_986c | HQ842033 | - |
| SK |  | SK083 | SK_988c | HQ842035 | HQ842549 |
| SK |  | SK083 | SK_989c3 | HQ842036 | - |
| SK |  | SK084 | SK_990c3 | HQ842037 | HQ842550 |
| SK |  | SK084 | SK_991c3 | HQ842038 | - |
| SK |  | SK087 | SK_992c | HQ842039 | HQ842551 |
| SK |  | SK087 | SK_993c3 | HQ842040 | - |
| SK |  | SK088 | SK_994c | HQ842041 | HQ842552 |
| SK |  | SK088 | SK_995c3 | HQ842042 | - |
| SK |  | SK089 | SK_996c | HQ842043 | HQ842553 |
| SK |  | SK089 | SK_997c3 | HQ842044 | - |
