# Supplementary material for: Using Mitochondrial and Nuclear Sequence Data for Disentangling Population Structure in Complex Pest Species: A Case Study with Dermanyssus gallinae
Source: PLoS One. 2011 Jul 25;6(7):e22305. doi: 10.1371/journal.pone.0022305 (PMC3143145; doi:10.1371/journal.pone.0022305)
Supplement: Methods S1 — Hypotheses under test and population genetics analyses. (DOC) [file pone.0022305.s008.doc]

## Methods S1

**Hypotheses under test and population genetics analyses**

To investigate the congruence of obtained data, we have explored the most probable causes for revealed patterns by testing different scenarios for interspecific hybridization, gene flows and demographic history. For such a purpose, we have confronted results obtained for different groups of isolates to each another in population genetics analyses, in relation with available information (see sampling place and context Appendix 1).

*A. Ancient hybridization and reversal of speciation*

Regarding a rather ancient time scale, the inclusion of the cryptic and sibling species L1 in hierarchical analyses is expected to serve as a level reference in order to detect species-level differentiation.

While methods exist to handle discordance among phylogenetic trees and estimate the degree of congruence and incongruence between different trees in order to estimate their mutual history (e.g. reconciliation of species tree with the gene trees to infer duplication / speciation nodes, [1], cophylogenetic studies in order to infer host-parasite coevolution scenarios, [2]), similar methods are lacking for population genetic data. Nevertheless, the model-based Bayesian software program Structure 2.3.3 allows visualizing individual clustering assignment and comparing it using different genes. Moreover, it allows detecting different levels of population structure and estimating the degree of admixture within individuals and intermingling within isolates.

If the present population structure is partly the result of past but relatively recent hybridization between incipient species (nearly interspecific hybridization), separation between some *D. gallinae* lineages would be expected to occur almost at the same level as L1 using a Bayesian assignment and clustering method following a hierarchical approach, as well as some Analyses of MOlecular VAriance (AMOVAs).

Specifically, a matrix of individual Tpm genotypes and a matrix of COI haplotypes, as well as a combined matrix of both genes were translated into the format eXtended Multi-Fasta Alignment (XMFA) by the mean of xmfa2struct [3]. NCBI accession numbers available for consultation as popsets are provided in Appendix 1a and correspondences between individuals and sequences in Appendix 1b).The XMFA matrices contain map distances between linked loci (here sites within each single gene fragment), which allows usage of the linkage model in Structure 2.3.3 [4]. In order to estimate whether different evolutionary rules affect indels and substitutions in this gene fragment, a fourth matrix was built based on the 8 following Tpm indels, as defined in [5]: A, B1, B2, D, F, G, H, J. Admixture model was tested in all analyses. Both admixture model and linkage model were tested on the combined matrix, since the map distance allows considering both genes independent. The log-likelihood of Tpm and COI sequence data was computed under an admixture model with correlated allele frequencies using Structure. We ran 10 replicates of each run. Each run consisted of a burn-in period of 120 000 MCMC (Markov chain Monte Carlo) steps followed by 600 000 iterations.

To infer the number of genetic groups in our data set, we used the ΔK method of [6]. It consists in finding the breakpoint in the slope of the distribution of lnP(D) for the different K values tested, where lnP(D) is an estimate of the posterior probability of the data for a given K. Calculation of ΔK was performed using Structure Harvester [7]. We then used a hierarchical approach, as described in [8], to detect all layers of structure, based on the fact that that this ΔK method detects the uppermost level of population structure [6]. As in [8], we established a >0.6 threshold as the minimal value for considering an individual assigned to a cluster. The number of genetic groups tested here was from K = 1 to K = 8 for each level of the analyses. Once first-level analyses in both genes was carried out, some additional analyses of the Tpm matrix were performed including mtDNA lineage affiliation as a population ID, and reciprocally, to identify admixed individuals whose admixture is not yet visible in single gene analyses.

Additionally, we evaluated the potential effect of linkage disequilibrium (LD) between loci within sequence. Indeed, a high r_LD_, a correlation coefficient estimated between pairs of loci [9], between closely linked loci in a dataset has been shown responsible in some cases for the overestimation of clusters using Structure [10]. As a result, we tested the combined data matrix using both the admixture model and the linkage model using map distance. Although the admixture model is very likely to be the most adequate in present case, the linkage model is expected to reveal eventual excess in the number of inferred clusters which may arise from LD between dependent loci (here between pairs of sites in each sequenced gene fragment). However, because of the sensitiveness of LD to demographic events, the distance between loci is not always a good indicator of the strength of LD. Consequently, data were also checked for linkage equilibrium (DnaSP 5.0) and, following recommendations of [10], some tests were performed with datasets excluding all pairs of loci with a r_LD_ significantly higher than 0.5. Significance was assessed using the two-tailed exact test of Fisher (P>0.05). Then obtained population structures in each hierarchical level were compared with structures obtained using the whole datasets.

*B. Demographic history*

The number of generations per year is expected to strongly differ between farm and wild isolates. Indeed, farms typically harbour highest host densities (several thousands of hens per on ground buildings and up to several hundreds of thousands of hens in a single cage building), provide relatively regular temperatures and humidity as well as hosts during most of the year (layer hen houses are usually bird-free for less than 2 months per year, during the “empty periods”). On the contrary, in wild bird environments, even in cases of bird colonies, the number of bird individuals relative to the size of the area they occupy is much smaller than in farms, temperature and humidity are much more variable than in farms during the year and even during the day and the host is available only during a limited period, *i.e.* the breeding period in spring-summer (and in a few cases some winter nights), which implies the total absence of bird host for several months in autumn and summer.

And yet, not only the presence of host is necessary for development completion in *D. gallinae,* since a blood meal is needed for ecdysis triggering at each stage and egg maturation at each laying, but also [11] have proved that mite development time is strongly dependent on temperature and hygrometry (low temperatures increase the development time). Tucci et al. [12] have shown that mite populations sampled from farms may complete one generation within one week in optimal temperature and hygrometry conditions, which are close to farm’s. As a result, one may expect farm populations to be able to complete up to 52 generations per year in such conditions. Since it is unlikely that optimal development time is maintained throughout the year, we consider 20-40 generations per year to be more reasonably expectable in layer farms (flock duration of 12 months), much less in broiler farms (flock duration of several weeks), since the duration of a broiler flock is much shorter than of a layer’s (several weeks in the formers, one year for the latter). In the wild avifauna, no experimental study has been carried out to our knowledge. Nevertheless, given host unavailability during non reproductive periods and much higher temperature variations, one may expect mite not to be able to complete more than 15 generations per year if considering a 1-week life cycle during the maximum breeding period (up to 3 months in case of multi-brood birds) and a few additional generations in case of birds staying in nest overnight in winter (not so common and accompanied by low temperatures). However, such estimates are likely to represent the upper bound of the true range, since only maximum duration for host availability and minimum life cycle are taken into account. Not to mention the fact that intensive farming conditions are likely to strongly affect parasite lifespan and evolution by selecting for faster parasite development [13]. This seems to be a consequence of the increased host density (which makes current reproduction more valuable in relation to future survival) and repeated anti-parasite treatments which is also likely to exacerbate selection for faster growth and transmission of parasites [14]. As a result, the mean number of generations per year in the wild avifauna may be much less than 15 and closer to 5-10.

*Bottlenecks and founder events*

The congruence between data and available knowledge concerning population demographic history will be tested by confronting known causes for bottlenecks and DNA-inferred demographic information.

Analyses

Old populations of constant size are characterized by multimodal mismatch distributions whereas expanding populations are unimodal [15]. The observed mismatch distribution between pairs of haplotypes within isolates, as well as among individuals grouped into some categories was tested for significant deviation from the sudden expansion model using Arlequin 3.11 [16], which also calculates the raggedness index of the mismatch distribution. The raggedness index takes high values when genealogies are diverse and few individuals share haplotypes, typical of multimodal mismatch distributions expected in constant populations.

Using DNASP 5.0, we also tested neutral nucleotide evolution by conducting Tajima’s D tests. Approximately equal values for measurements of genetic variation based on the number of segregating sites (S) or average pairwise nucleotide diversity (π) are expected at mutation-drift equilibrium under a standard neutral model. Tajima’s D statistics quantifies under an infinite site model departures from this neutral expectation, and values different from zero suggest the action of nonneutral selective, population expansion, bottleneck, or heterogeneity of mutation rates [17,18,19]. Considering demographic expansions or contractions, the number of segregating sites is influenced by the size of current population more strongly than is the average number of nucleotide differences, while the average number of nucleotide differences is affected by the size of original population more severely than is the number of segregating sites [20]. When an expansion of population is in process, the number of nucleotide differences is therefore high relatively to the number of segregating sites, which translates into a positive D statistic, and conversely.

Known causes for bottlenecks and founder events

Not only a variety of causes for repeated bottlenecks and founder events are found in both farms and wild avifauna, but also *Dermanyssus* species seem to be intrinsically well equipped for tolerating repeated successive bottlenecks and then efficiently expand. Indeed, discrepancies in the host availability, hard winter conditions and swarming behaviour are expected to be responsible for cyclically repeated bottlenecks in mites parasitizing wild birds, while pesticides’ applications, long distance transportations for slaughtering, and importation/exportation processes involving quarantine and other sanitary practices do so in fowl environments. And yet, some empirical studies have shown in *D. hirundinis* and in *D. americanus* that populations become seasonally the smallest in wild conditions, during winter and in the absence of host [21,22]. Then, starting with extremely small founder population, a rapid and remarkable increase of population has been observed during breeding period by the same authors. And in farms, for instance, [23] have reported an increase of 400% of *D. gallinae* population within 42 days during a farm trial. All the more, recurrently bottlenecked populations have been recently revealed by some population genetics analyses in a close dermanyssoid family composed of bat parasites [24].

As for farm isolates, pesticides composed of pyrethroids, carbamates, organophosphates and/or amitraz have been very commonly and recurrently used in most of sampled farms during flocks regardless of regulations and without any standards from 2001 to 2007. This occurred mainly because no allowed synthetic molecule were available for the flock period in layers, consecutively to the codification exercise provided by directive 2001/82/EC1 prescribing that veterinary medicinal products can only be authorised or used in food producing animals if pharmacologically active substances contained therein have been assessed as safe according to Regulation (EEC) No 2377/90 (‘Regulation on Maximum Residue Levels’). The consecutive lack of allowed molecule generated anarchical control practices, till 2007 in France. Up to 18 pesticide applications per flock (*ie* per year) have been reported in French farms during the years before sampling (2006-2009, farmers, pers. comm.). In 2007, one organophosphate-based product, has proven not exceeding MRLs in eggs and became allowed during layer flocks. And before MLRs have been imposed in Europe, various pesticide families were allowed during flock, not to mention the frequent use of fuel oil against the poultry red mite (a farmer, pers. comm.).

*C. Gene flows*

At a much more recent time scale, some population intermingling should be expected due to current expansion of commercial networks. In order to discriminate between the role of wild birds and the role of trade flows involving poultry transportation (young chicks at the beginning of any flock, spent hens at the end of a flock) in mite dispersal, two main questions are to be answered. First, are mites able to enter farms by the mean of wild birds? Any gene flows between mite populations in sampled wild bird nests and in farms will be searched for (*habitat effect*). In such bird parasites, it is likely that geographical isolation is reduced in the wild avifauna, due to the mobility of their host. Indeed, it is very unlikely that migration of birds could play any role in the long-distance dissemination of *D. gallinae*, since it is a nidicolous micropredator [25]. Although it may punctually transit from one place to another by the mean of the bird host, it is very unlikely that it can stay on the host during one or two seasons. Arkle et al. [26] have shown that the percentage of death in individuals constrained to stay on host is much more elevated than in individuals allowed to stay off host. And yet, during the migration period, no off-host hiding-place is available for mite to survive. As a result, overall, gene flows on a large scale distances are not expected to be explainable by bird intervention.

Second, are mites able to transit by the mean of man, hen, human motorized vehicles to move from one place to another within poultry industries? In order to answer this question, farm isolates originating from farms separated by distances of various scales will be confronted to each another, taking into account available knowledge on trade routes (*trade network effect*): (1) transcontinental scale (Europe, Australia, Brazil), (2) country scale (within Europe: France, Denmark, Poland), (3) regional scale (within France, 2 regions) and (4) even farm scale (one focused farm labelled CON). Concerning scales 1 and 3, several known trade routes are to be considered: at the world level, a consolidation occurred during the last 50 years at all phases of the poultry industry [27], leading to strongly increase the size and intensity of inter-continent exchanges. Among others, standard layer and broiler strains were recurrently exported from Europe to Australia [28] and to Brazil [29]. Within France (3), whereas many small slaughterhouses were available around 10-50 km apart in the Bresse region (France, Rhône-Alpes), suddenly, ten years ago, their merging to larger – and distant - slaughter processing plants have forced layer farmers to send spent hens from Bresse to Brittany (North-Western France, >800km apart) or Spain (S. Lubac, ITAVI, pers. comm.). At the farm scale (4), isolates from farm CON were sampled in two different buildings (78 m apart, 150 m by feet), at two points (ca. 25 m apart from each other; see Appendix 2) within each building. Additionally, despite above evoked consolidation in all phases of the poultry industry, including breeders of layer strains and of broiler strains, some smaller farms producing pure lines keep economically separated for speciality markets [27] such as “Poulets de Bresse” in France. Since only rarely same trucks may be used to move birds of such two separated industries from young chick producer to farm and from farm to slaughter processing plant, any presence of gene flows between two different industries would be interpreted as an insight of the capacity of mites to transit by switching from cage to truck then truck to cage and in small numbers. Lastly, as numerous farmers witness of recurrent infestations in many farms, from one hen flock to another despite the empty period (period of 2-8 weeks during which drastic disinfectant and/or acaricide treatments are processed), the question of the efficiency will be explored by comparing isolates sampled before and after the empty period in a single building (farm CON, *cleaning effect*). If persistence of mite populations between such drastic cleaning actions as those performed during the empty period is confirmed, then the ability of mite to also survive into cleaned trucks, as well as during quarantine periods in the framework of importations would be the most probable.

Datasets were checked for departures from Hardy–Weinberg by the mean of F_IS_ estimates based on our diploid Tpm sequence dataset (Arlequin version 3.11). To visualize mitochondrial and nuclear sequence variation and haplotype geographical and ecological distribution for the sampled mites, we constructed median-joining haplotype networks (MJN) using Network 4.510 with post-processing parsimony analyses ([30], www.fluxus-engineering.com).

In order to determine the most important source of structuration, four types of groups were confronted to each another, three of which directly originating from ecological information associated with samples (see Appendix 1) and the fourth one resulting from conducted clustering analyses (Structure), were used to conduct analyses of molecular variance (AMOVA) [16] in Arlequin: geographical criterion, industry effect (fowl only, two separated industries: Bresse AOC broiler, layer), habitat (wild avifauna, farms), clusters indicated in first-level Structure combined analysis. Analyses were performed with genetic variation being partitioned into three levels when using COI dataset (among groups, among isolates within groups, within isolates) and four levels when using Tpm dataset (among groups, among isolates within groups, among individuals within isolates, within individuals). Additionally, in order to estimate inter-isolates gene flows, pairwise F_ST_ values were calculated in Arlequin 3.11. Their significance was tested with the permutation test (1000 permutations).

### *Recent introgressive hybridization and the distribution of some farm mt haplotypes*

Both mitochondrial and nuclear genetic diversity are expected to be proportional to a population’s effective population size. However, at least initially, introgression of a divergent allele could lead to an increase in mitochondrial genetic diversity relative to a population’s nuclear genetic diversity. Thus, if some populations possess some possibly foreign COI haplotypes due to recent introgression, as opposed to ancestral polymorphism, they should have a higher ratio of mitochondrial genetic diversity to nuclear genetic diversity than other populations. To test this hypothesis we compared the ratio of mitochondrial diversity to nuclear diversity for all sampled isolates^+^. As a measure of COI and Tpm nucleotide diversity, π [18,31] was calculated for each isolate using Arlequin 3.11 [16].

**References**

1. Page RDM, M.A. C (1997) From Gene to Organismal Phylogeny: Reconciled Trees and the Gene Tree/Species Tree Problem. Molecular Phylogenetics and Evolution 7: 231-240.

2. Charleston MA (2006) Traversing the tangle: Algorithms and applications for cophylogenetic studies. Journal of Biomedical Informatics 39: 62-71.

3. Didelot X (2009) xmfa2struct

4. Pritchard JK, Wen W (2004) Documentation for structure software: version 2.

5. Roy L, Dowling APG, Chauve CM, Buronfosse T (2010) Diversity of Phylogenetic Information According to the Locus and the Taxonomic Level: An Example From a Parasitic Mesostigmatid Mite Genus. International Journal of Molecular Sciences 11: 1704-1734.

6. Evanno G, Regnaut S, Goudet J (2005) Detecting the number of clusters of individuals using the software structure: a simulation study. Molecular Ecology 14: 2611-2620.

7. Earl DA (2009) Structure Harvester v0.3.

8. Coulon A, Fitzpatrick JW, Bowman R, STITH BM, MAKAREWICH CA, et al. (2008) Congruent population structure inferred from dispersal behaviour and intensive genetic surveys of the threatened Florida scrub-jay (*Aphelocoma coerulescens*). Molecular Ecology 17: 1685-1701.

9. Hill WG, Robertson A (1968) Linkage disequilibrium in finite populations. Theoretical and Applied Genetics 38: 226-231.

10. Kaeuffer R, Réale D, Coltman DW, Pontier D (2007) Detecting population structure using STRUCTURE software: effect of background linkage disequilibrium. Heredity 99: 374-380.

11. Nordenfors H, Höglund J, Uggla A (1999) Effects of Temperature and humidity on oviposition, molting, and longevity of *Dermanyssus gallinae* (Acari : Dermanyssidae). Journal of Medical Entomology 36: 68-72.

12. Tucci EC, Prado AP, Araujo RP (2008) Development of *Dermanyssus gallinae* (Acari: Dermanyssidae) at different temperatures Veterinary parasitology 155: 127-132.

13. Mennerat A, Nilsen F, Ebert D, Skorping A (2010) 2010. Intensive farming: evolutionary implications for parasites and pathogens. . Evolutionary Biology 2-3: 59-67.

14. Webster JP, Gower CM, Norton AJ (2008) Evolutionary concepts in predicting and evaluating the impact of mass chemotherapy schistosomiasis control programmes on parasites and their hosts. . Evolutionary Applications 1: 66-83.

15. Harpending HC, Batzer MA, Gurven M, Jorde LB, Rogers AR, et al. (1998) Genetic traces of ancient demography. Proceedings of the National Academy of Sciences 95: 1961-1967.

16. Excoffier LG, Laval L, Schneider S (2005) Arlequin ver. 3.0: An integrated software package for population genetics data analysis. Evolutionary Bioinformatics Online 1: 47-50.

17. Tajima F (1989) Statistical method for testing the neutral mutation hypothesis by DNA polymorphism. Genetics 123: 585-595.

18. Tajima F (1993) Measurement of DNA polymorphism. In: Takahata N, Clark AG, editors. Mechanisms of Molecular Evolution Introduction to Molecular Paleopopulation Biology. Tokyo: Japan Scientific Societies Press, Sinauer Associates, Inc. pp. 37-59.

19. Tajima F (1996 ) The amount of DNA polymorphism maintained in a finite population when the neutral mutation rate varies among sites. Genetics 143: 1457-1465.

20. Tajima F (1989) The Effect of Change in Population Size on DNA Polymorphism. Genetics 123: 597-601.

21. Burtt EHJ, Chow W, Babitt GA (1991) Occurrence and demography of mites of tree swallow, house wren, and eastern bluebird boxes. In: Loye JE, Zuk M, editors. Bird-parasite interactions: ecology, evolution and behaviour. Oxford: Oxford University Press. pp. 104-122

22. Phillis W (1972) Seasonal abundance of *Dermanyssus hirundinis* and *D. americanus* (Mesostigmata: Dermanyssidae) in nests of the House Sparrow. Journal of Medical Entomology 9: 111-112.

23. Meyer-Kühling B, Pfister K, Müller-Lindloff J, Heine J (2007) Field efficacy of phoxim 50% (ByeMite®) against the poultry red mite *Dermanyssus gallinae* in battery cages stocked with laying hensField efficacy of phoxim 50% (ByeMite®) against the poultry red mite Dermanyssus gallinae in battery cages stocked with laying hens. Veterinary Parasitology 147: 289-296.

24. Bruyndonckx N, Henry I, Christe P, Kerth G (2009) Spatio-temporal population genetic structure of the parasitic mite *Spinturnix bechsteini* is shaped by its own demography and the social system of its bat host. Molecular ecology 18: 3581-3592.

25. Roy L (2009) Evolutionary ecology of a hematophagous mite genus: Phylogeny-based approach for interspecific delineations and comparative characterization of populations in five species of the genus *Dermanyssus* (Acari: Mesostigmata). Paris: Institut des Sciences et Industries du Vivant et de l’Environnement (Agro Paris Tech). 307 p.

26. Arkle S, George DR, Guy JH, Sparagano OAE (2010) Comparison of *in vivo* and *in vitro* survival and fecundity rates of the poultry red mite, *Dermanyssus gallinae*. Research in Veterinary Science 88: 279-280.

27. Agriculture and Agri-Food Canada (2010) Overview of the Primary Poultry Breeder Industry.

28. Scott P, Turner A, Bibby S, Chamings A (2005 ) Structure and Dynamics of Australia’s Commercial Poultry and Ratite Industries. Victoria: The Department of Agriculture, Fisheries and Forestry of Australia.

29. Dalla Costa AJ (1997) Agroindústria Brasileira contemporânea : inovações organizacionais e transformações tecnológicas na avicultura. Paris: Université de la Sorbonne Nouvelle Paris III

30. Bandelt H-J, Forster P, Röhl A (1999) Median-joining networks for inferring intraspecific phylogenies. Molecular Biology and Evolution 16: 37-48.

31. Tajima F (1983 ) Evolutionary relationship of DNA sequences in finite populations. Genetics 105 437.
